# Accurate fusion transcript identification from long- and short-read isoform sequencing at bulk or single-cell resolution

**DOI:** 10.1101/gr.279200.124

**Published:** 2025-04

**Authors:** Qian Qin, Victoria Popic, Kirsty Wienand, Houlin Yu, Emily White, Akanksha Khorgade, Asa Shin, Christophe Georgescu, Catarina D. Campbell, Arthur Dondi, Niko Beerenwinkel, Francisca Vazquez, Aziz M. Al'Khafaji, Brian J. Haas

**Affiliations:** 1 Broad Institute of MIT and Harvard, Cambridge, Massachusetts 02142, USA;; 2 Department of Biosystems Science and Engineering, ETH Zurich, 4056 Basel, Switzerland;; 3 SIB Swiss Institute of Bioinformatics, 4056 Basel, Switzerland

## Abstract

Gene fusions are found as cancer drivers in diverse adult and pediatric cancers. Accurate detection of fusion transcripts is essential in cancer clinical diagnostics and prognostics and for guiding therapeutic development. Most currently available methods for fusion transcript detection are compatible with Illumina RNA-seq involving highly accurate short-read sequences. Recent advances in long-read isoform sequencing enable the detection of fusion transcripts at unprecedented resolution in bulk and single-cell samples. Here, we developed a new computational tool, CTAT-LR-Fusion, to detect fusion transcripts from long-read RNA-seq with or without companion short reads, with applications to bulk or single-cell transcriptomes. We demonstrate that CTAT-LR-Fusion exceeds the fusion detection accuracy of alternative methods as benchmarked with simulated and genuine long-read RNA-seq. Using short- and long-read RNA-seq, we further apply CTAT-LR-Fusion to bulk transcriptomes of nine tumor cell lines and to tumor single cells derived from a melanoma sample and three metastatic high-grade serous ovarian carcinoma samples. In both bulk and single-cell RNA-seq, long isoform reads yield higher sensitivity for fusion detection than short reads with notable exceptions. By combining short and long reads in CTAT-LR-Fusion, we are able to further maximize the detection of fusion splicing isoforms and fusion-expressing tumor cells.

Genomic rearrangements involving chromosomal translocations or deletions can yield fusion genes, in some cases activating oncogenes or disabling tumor suppressors and contributing to cancer. While most cancer-relevant fusion genes are found at low levels of recurrence in surveys of diverse tumor types, certain fusions represent hallmark drivers of cancer found at high levels of recurrence, such as *BCR::ABL1* in chronic myelogenous leukemia (CML) (Kurzrock et al. 1988), *SS18::SSX1 or SS18::SSX2* (Ren et al. 2013) in synovial sarcoma, and *TMPRSS2::ERG* (Wang et al. 2017) in prostate cancer. Several gene fusions serve as diagnostic markers for certain pediatric cancers, including *EWSR1::FLI1* for Ewing's sarcoma (May et al. 1993), *ETV6::RUNX1* in acute lymphoblastic leukemia (Sundaresh and Williams 2017), *PVT1::MYC* in medulloblastoma (Northcott et al. 2012), and *PAX3::FOXO1* in rhabdomyosarcoma (Linardic 2008). The molecular mechanisms by which gene fusions contribute to cancer can widely vary from positioning the 3′ fused gene under the promoter and gene expression regulatory elements of the 5′ gene, or encoding fusion proteins with altered molecular functions, all leading to alterations in the cellular circuitry that ultimately drive uncontrolled cellular proliferation.

Identification of gene fusions has been an essential part of charting the landscape of cancer genomic variations, deriving biomarkers for molecular diagnostics of cancer patients, and targeting therapies such as tyrosine kinase inhibitors for the treatment of kinase gene fusions such as *BCR::ABL1* in CML patients (Cuellar et al. 2018) and *EML4::ALK* (Christopoulos et al. 2018) in lung cancer. Transcribed and translated gene fusions are of particular interest toward discovering neoantigens in targeted immunotherapies (Yang et al. 2019; Wang et al. 2021b; Bauer et al. 2022) and yield additional opportunities for targeting immunotherapies toward cancers with low mutational burdens. During the past decade, RNA-seq has been the preferred assay for comprehensive gene fusion detection due to its lower cost than whole-genome sequencing (WGS) and directly measuring the transcripts arising from the gene fusions. Illumina short-read RNA-seq has become routine for such studies, and numerous computational methods have been developed to predict fusions from Illumina RNA-seq (Kim and Salzberg 2011; Li et al. 2011; McPherson et al. 2011; Benelli et al. 2012; Jia et al. 2013; Wang et al. 2013; Davidson et al. 2015; Latysheva and Babu 2016; Okonechnikov et al. 2016; Rodríguez-Martin et al. 2017; Akers et al. 2018; Haas et al. 2019; Uhrig et al. 2021). Primarily through studies of Illumina RNA-seq, large catalogs of fusions have been compiled across large collections of tumor and normal tissues (Klijn et al. 2015; Yoshihara et al. 2015; Babiceanu et al. 2016; Hu et al. 2018; Dehghannasiri et al. 2019; Haas et al. 2023). Fusion transcripts relevant to cancer tend to involve genome rearrangements, whereas fusion transcripts identified in normal tissues tend to derive from *cis*- or *trans*-splicing or otherwise derive from natural population structural variants yielding population-specific *cis*-spliced fusion transcripts (Nigro et al. 1991; Li et al. 2008, 2009; Chase et al. 2010; Boettger et al. 2012; Qin et al. 2015).

While short RNA-seq reads have been highly useful for identifying fusion gene candidates and resolving fusion transcript isoform breakpoints, the reads are not long enough to resolve the complete isoforms that are expressed, and additional transcript reconstruction methods are needed to infer potential full-length fusion transcripts. Short-read RNA-seq methods that involve targeted sequencing of the 3′ or 5′ terminus of RNA molecules, which are currently standard in high-throughput single-cell sequencing assays, pose further limitations for fusion detection as short reads are far less likely to span the breakpoint of the fusion transcript.

Long-read isoform sequencing is made possible by Pacific Biosciences (PacBio) and Oxford Nanopore Technologies (ONT), enabling full-length isoform sequences via their cDNA, or in the case of ONT, the option of direct RNA sequencing. Early applications of these technologies have been constrained due to low throughput and high error rates. Recent advances in both long-read platforms have enabled high-throughput long-read transcriptome sequencing at high sequencing accuracy (on par or exceeding that of conventional short-read sequencing) (Wenger et al. 2019; Marx 2023). Applications of long isoform reads have enabled deeper insights into transcriptome isoform diversity in whole tissues (Glinos et al. 2022; Reese et al. 2023), and most recently for single cells (Al'Khafaji et al. 2024). Applications of long-read RNA-seq is gaining traction in the cancer research community, particularly involving fusion isoform detection, with several computational methods now available that are specifically tailored toward characteristics of long reads (Liu et al. 2020; Davidson et al. 2022; Chen et al. 2023). However, as long-read isoform sequencing technology has been rapidly advancing and most computational tools for fusion detection have only recently been developed, there has been limited work thus far toward benchmarking their capabilities or applying them in new areas such as fusion detection in single cells.

To further advance fusion transcript detection using long-read isoform sequencing, we developed a new method as part of the Cancer Transcriptome Analysis Toolkit (CTAT) called CTAT-LR-Fusion. CTAT-LR-Fusion is specifically developed for long-read RNA-seq with or without short-read RNA-seq as a modularized software that contains chimeric read extraction, fusion transcripts identification, expression quantification, gene fusion annotation, and interactive visualization. To benchmark existing tools, we collected or generated comprehensive simulation data sets to reflect varied sequencing technologies and sequencing error rates. We also designed new experiments to profile a normal cell line transcriptome with spiked-in known oncogenic fusion transcripts and nine cancer cell lines using the same long-read sequencing protocol MAS-ISO-seq (Al'Khafaji et al. 2024). In both simulation and real data sets, we systematically benchmarked CTAT-LR-Fusion accuracy in comparison to available long-read fusion tools. We finally applied CTAT-LR-Fusion to long isoform read sequences derived from tumor single-cell transcriptomes including melanoma and high-grade serous ovarian carcinoma (HGSOC) metastases. In all experiments with real data, we used available sample-matched Illumina short reads, or generated companion Illumina RNA-seq for comparison to long isoform reads and to augment findings based on long reads.

## Results

### CTAT-LR-Fusion pipeline

**Figure 1.**
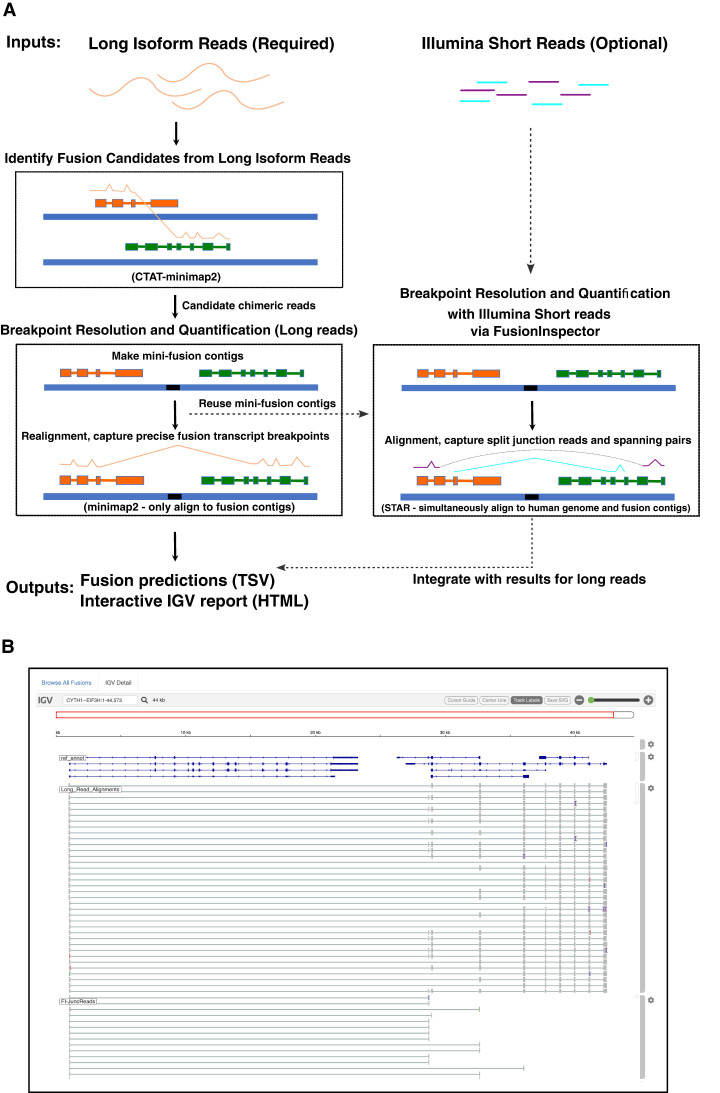
CTAT-LR-Fusion workflow and output. (*A*) CTAT-LR-Fusion workflow: Long isoform read sequences and optional matched short RNA-seq reads are provided as input. Long reads are aligned to the reference genome using ctat-minimap2, a slightly modified minimap2 tool that yields alignments only for reads that map to multiple genomic loci. Reads identified as candidate chimeric reads are further assessed as fusion evidence by modeling fusion contigs containing the candidate fusion genes in their proper order and orientation followed by realignment of candidate chimeric reads to the fusion contigs using regular minimap2. Fusion transcript breakpoints and fusion read support are quantified in the context of the fusion contigs. If matched short reads are available, FusionInspector (included with CTAT-LR-Fusion) uses STAR to simultaneously align the short reads to the fusion contigs and the human reference genome, yielding fusion evidence quantification based on short-read RNA-seq. The FusionInspector findings based on short-read RNA-seq are then integrated into the final fusion report generated by CTAT-LR-Fusion, specifying the fusion evidence support for fusion transcript isoforms by long and/or short reads according to breakpoint. Outputs include tab-delimited reports describing the fusion alignment evidence and quantifications and an interactive IGV-report in HTML format for navigating the fusion alignment evidence. (*B*) Example IGV-report highlighting evidence for fusion *CYTH1::EIF3H* in cell line SKBR3. The IGV-report visualization provides interactive analysis of reference annotation gene structures (*top*) and long isoform read alignment evidence for predicted fusion transcripts (*center*) and includes alignments for matched Illumina short reads where available (*bottom*). In this example, multiple alternatively spliced fusion isoforms are evident from both long and short reads, and most long reads appear to yield full-length fusion transcript isoforms, whereas the short reads primarily yield fusion isoform breakpoint positions.

Fusion transcript detection from long reads by CTAT-LR-Fusion involves two phases (Fig. 1A). In the first phase, candidate chimeric long reads are rapidly identified using a customized version of the minimap2 aligner (Li 2018) that only reports alignments for reads with preliminary mappings to multiple genomic loci. Candidate chimeric reads and corresponding fusion gene pairs are identified based on these preliminary alignments. In the second phase, candidate fusion gene pairs are modeled as collinear genes, and the candidate chimeric reads are realigned to the fusion contigs using minimap2 full alignment (an adaptation of methods in FusionInspector [Haas et al. 2023] for short-read RNA-seq that we integrated into CTAT-LR-Fusion for long-read isoform sequences). Fusion genes are identified based on high-quality read alignments and fusion transcript breakpoints are quantified based on the number of supporting long isoform fusion reads (see Methods for details). If sample-matched Illumina RNA-seq is available, our earlier-developed FusionInspector is further executed to capture short-read alignment evidence for these fusion candidates, and the FusionInspector results are integrated with the long-read results into the final CTAT-LR-Fusion report. Long reads (with short reads where applicable) alignment evidence for fusion transcripts are made available for further navigation via the included interactive web-based IGV-report (Fig. 1B) or separately via desktop Integrative Genomics Viewer (IGV) (Robinson et al. 2011).

### Fusion transcript detection accuracy using simulated long reads

Earlier benchmarking of fusion transcript detection by JAFFAL (Davidson et al. 2022) entailed the use of Badread (Wick 2019) to simulate long reads for fusion transcripts based on PacBio and ONT error models and spanning a wide range of sequence divergence from 25% error (75% alignment identity) to 5% error (95% alignment identity), targeting 500 simulated fusion gene pairs represented per data set with 1× to over 100× long-read coverage (detailed in Davidson et al. 2022; Supplemental Table S1). We leveraged these available test data to examine CTAT-LR-Fusion accuracy in comparison to available alternatives, including JAFFAL (Davidson et al. 2022), LongGF (Liu et al. 2020), FusionSeeker (Chen et al. 2023), and pbfusion (Volden et al. 2023).

**Figure 2.**
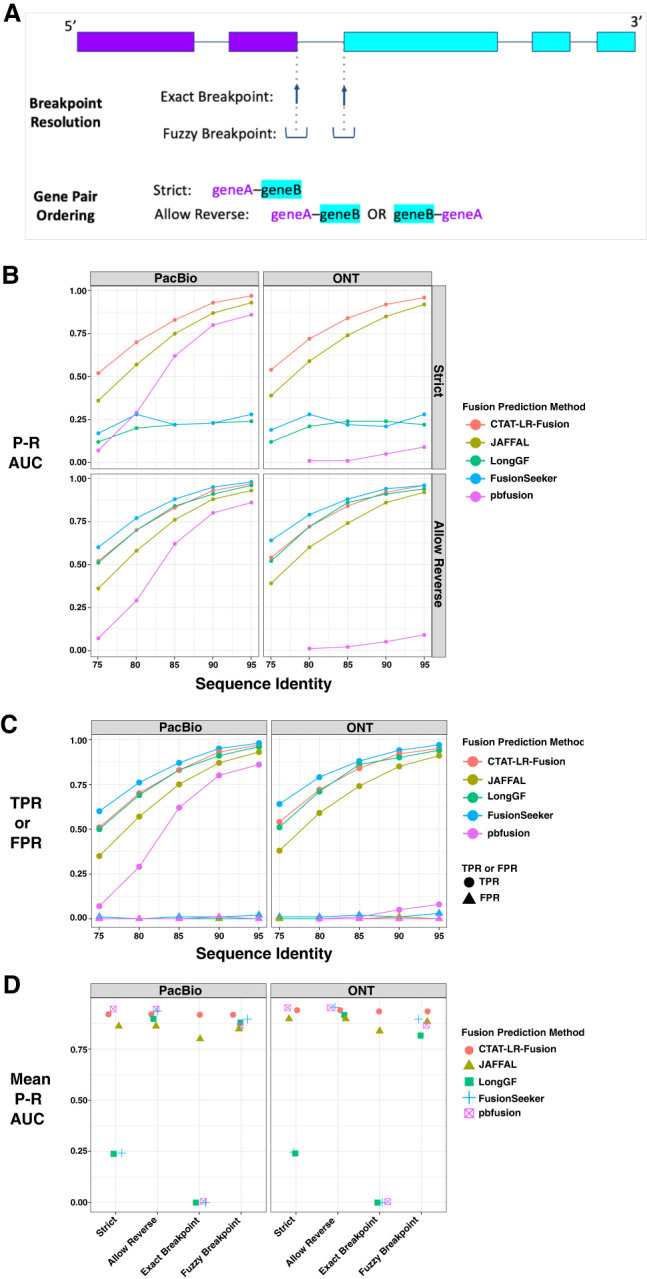
Accuracy for fusion transcript detection using simulated long reads. (*A*) Scheme for criteria in benchmarking fusion detection. (*B*) Accuracy is reported as P–R AUC using simulated PacBio and ONT long reads with moderate to high error rates (test data derived from Davidson et al. 2022), requiring proper fusion gene pair ordering (Strict) or allowing for either gene pair ordering (Allow Reverse). (*C*) True positive rate (TPR) and false positive rate (FPR) were observed for each method and data set at corresponding peak F1 scores, allowing for either fusion gene pair ordering (Allow Reverse). (*D*) Accuracy using PBSIM3 simulated PacBio HiFi or ONT R10.4.1 isoform reads at 5× coverage additionally focused on breakpoint resolution, with mean of P–R AUC values across five samples of 500 different target fusions each. Similar findings resulted at 50× coverage (Supplemental File 2).

For each long-read fusion transcript detection method, we computed precision, recall, corresponding F1 accuracy score according to minimum read support, and the area under the precision–recall curve (P–R AUC) to evaluate accuracy for each test data set representative of sequencing technology (PacBio or ONT) and error rate (75%–95% sequence identity) (Fig. 2A–C; Supplemental Table S2). Since fusion genes can sometimes be misidentified as closely related paralogs, we allowed paralogs of fusion genes to serve as proxies and scored them as equivalent to fusions in the truth set (as in Haas et al. 2019). Only CTAT-LR-Fusion, JAFFAL, and pbfusion (since version 0.4.0) properly report fusion gene pairs in the order in which they are fused together from 5′ to 3′ in the corresponding fusion transcript, and so only CTAT-LR-Fusion, JAFFAL, and pbfusion exhibit high accuracy when benchmarking fusion detection in a “strict” manner requiring ordered gene pairs. Relaxing this requirement and scoring fusion detection based solely on unordered gene pairings, all methods demonstrate moderate to high fusion detection accuracy at the lowest sequence divergence (95% identity) for both PacBio and ONT simulated reads. Fusion detection recall and overall accuracy improve with read sequence quality for all methods; all methods reported few to no false positives (FPs) at peak accuracy (F1 score) with these simulated data (Fig. 2C). In comparison to the other methods, pbfusion was most sensitive to sequence error rates, least capable of fusion detection with the highest error rates and largely incompatible with these ONT simulated reads—as could be expected given that pbfusion was developed by PacBio for highly accurate PacBio Iso-Seq data. Overall, CTAT-LR-Fusion and JAFFAL were found to be top-performing with these simulated test data when considering fusion gene order and orientation, with CTAT-LR-Fusion providing top performance across most combinations of error rates and sequencing technology. Notably, both LongGF and FusionSeeker leverage reads that are prealigned to the genome as input, and both methods are sensitive to the alignment parameters used when applied to reads with higher sequencing error rates (Supplemental Fig. S1). When ignoring fusion gene pair ordering and optimal read alignment parameters for divergent sequences, FusionSeeker and LongGF demonstrate high accuracy on these simulated reads.

While the above test data were useful to differentiate fusion detection recall according to sequence divergence, the sequence error rates do not reflect those of the currently available long-read sequencing technologies, which have rapidly improved to now routinely yield long-read sequences at 1% (Q20) to 0.1% error (Q30) or better (Marx 2023). To that end, we used PBSIM3 (Ono et al. 2022) to simulate PacBio HiFi and ONT R10.4.1 long reads and further investigated fusion transcript detection accuracy across methods. We simulated long reads for five samples with 500 fusion transcripts each and read coverage targeted at 5× sequence coverage. With these newly simulated reads, all methods demonstrated high fusion transcript detection accuracy when considering only the unordered pairs of genes. To further explore differences in the accuracy characteristics of these methods, we evaluated their fusion transcript breakpoint detection accuracy (Fig. 2D). In particular, we compared the known simulated fusion breakpoints to the chromosomal location of the estimated fusion transcript breakpoint at each gene for each method. Similar to the fusion gene ordering, only CTAT-LR-Fusion and JAFFAL demonstrated highly accurate fusion transcript breakpoint detection (ignoring gene ordering during breakpoint evaluation). While FusionSeeker, LongGF, and pbfusion demonstrated little capacity for detecting exact breakpoints, the vast majority of breakpoints they reported were within a short distance (±5 bases) from the ground truth breakpoints (Fig. 2D).

### Long-read fusion isoform detection with a reference fusion control RNA sample

To evaluate CTAT-LR-Fusion with real transcriptome sequencing data, we leveraged a commercial reference RNA sample from SeraCare (Seraseq Fusion RNA Mix v4) containing a set of 16 clinically relevant fusion transcripts mixed at a fixed concentration into a background of total RNA derived from a commonly used human cell line (GM24385). This reference RNA sample was sequenced for long reads using our newly developed MAS-ISO-seq method (Al'Khafaji et al. 2024) commercialized by PacBio as Kinnex for augmented sequencing throughput. Sequencing was performed in triplicate, with replicate-1 using MAS-ISO-seq in a monomeric format (similar to standard PacBio Iso-Seq) and replicates-2 and -3 using the standard MAS-ISO-seq 8-mer concatamer format (as in Kinnex). The higher sequencing depth (Supplemental Table S1) of the standard MAS-ISO-seq data sets yielded more long fusion reads than the monomer-based (Iso-Seq-like) library construction, but after normalization for sequencing depth, the rate of recovery of fusion reads was roughly equivalent, consistent with the sequencing libraries being derived from the single sample (Supplemental Fig. S2A). For comparison of fusion detection with PacBio long isoform reads versus Illumina short-read RNA-seq, we further sequenced this Seraseq fusion reference standard using Illumina TruSeq as triplicate libraries with paired-end 151 base length reads. Both MAS-ISO-seq and TruSeq generated ∼5–10 M reads (or paired-end sequences for TruSeq) per replicate (Supplemental Table S1).

**Figure 3.**
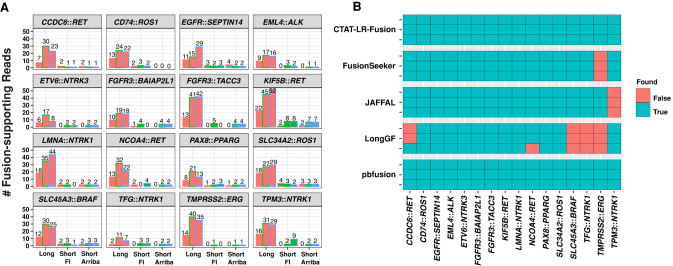
Fusion transcript detection applied to SeraCare v4 Fusion Reference Control sample. (*A*) Quantities of PacBio long reads and TruSeq Illumina short reads identified as evidence for each of the 16 control fusions as ascertained by CTAT-LR-Fusion (long reads), FusionInspector (short reads), and Arriba (short reads) across each sample replicate. PacBio replicate reads were down-sampled to match the number of sequenced bases from the respective Illumina replicate samples. (*B*) Binary heatmap for the identification of the 16 control fusion pairs by different fusion detection software according to each of the three replicates of long-read sequences, using all (not down-sampled) sequenced reads. PacBio replicates are ordered (*A*) *left* to *right* or (*B*) *top* to *bottom* as MAS-ISO-seq monomer (replicate 1), and MAS-ISO-seq 8mer-concatamer sequenced replicates 2 and 3. Counts of sequenced reads are provided in Supplemental Table S1.

Before comparing fusion detection between long and short reads with the Seraseq fusion sequencing data, we first down-sampled the PacBio MAS-ISO-seq reads to match total sequenced bases from the Illumina sequenced sample replicates, respectively. All 16 control fusions were detected by CTAT-LR-Fusion across three down-sampled replicates with a range of 2–52 long PacBio isoform reads per sample (Fig. 3A). Although matched Illumina TruSeq RNA-seq was performed for each of three replicates and overall gene expression was significantly positively correlated between long- and short-read sequencing (Supplemental Fig. S2B,C), few to zero control fusion-supporting reads were detected across three replicates based on the Illumina short reads; all fusions were detected in at least one TruSeq replicate across all samples, but short fusion reads were missing in at least one replicate for 6/16 control fusions with neither FusionInspector or Arriba reporting Illumina-based fusion reads (Fig. 3A). Given the matched sequencing depth (sequenced bases) and based on additional comparisons with cancer cell line transcriptomes (see below), we would expect similar numbers of long and short reads to be reported as fusion evidence, but here MAS-ISO-seq proved far more effective for Seraseq fusion isoform sequencing and detection than via Illumina RNA-seq.

We examined the alternative long-read fusion transcript detection methods for identification of the 16 control fusions using all PacBio sequenced long isoform reads with no down-sampling (Fig. 3B). Only CTAT-LR-Fusion and pbfusion (as of v0.4.0) were found to identify each of the 16 control fusions across each of the three long-read sequencing libraries. FusionSeeker and JAFFAL each failed to report one of the 16 fusions, each a different fusion and consistent across all replicates. LongGF, while having high accuracy for the detection of fusions with simulated data, was found least effective here with each replicate missing 4/16 control fusions, and reporting either *CCDC6::RET* or *NCOA4::RET* fusions but not both together in any single replicate. *TMPRSS2::ERG*, the hallmark fusion of prostate cancer, failed to be reported by both LongGF and FusionSeeker, while CTAT-LR-Fusion detects 45, 98, and 104 long isoform reads supporting *TMPRSS2::ERG* across the three sequenced libraries.

### Long-read fusion isoform detection from MAS-ISO-seq of nine cancer cell lines

**Figure 4.**
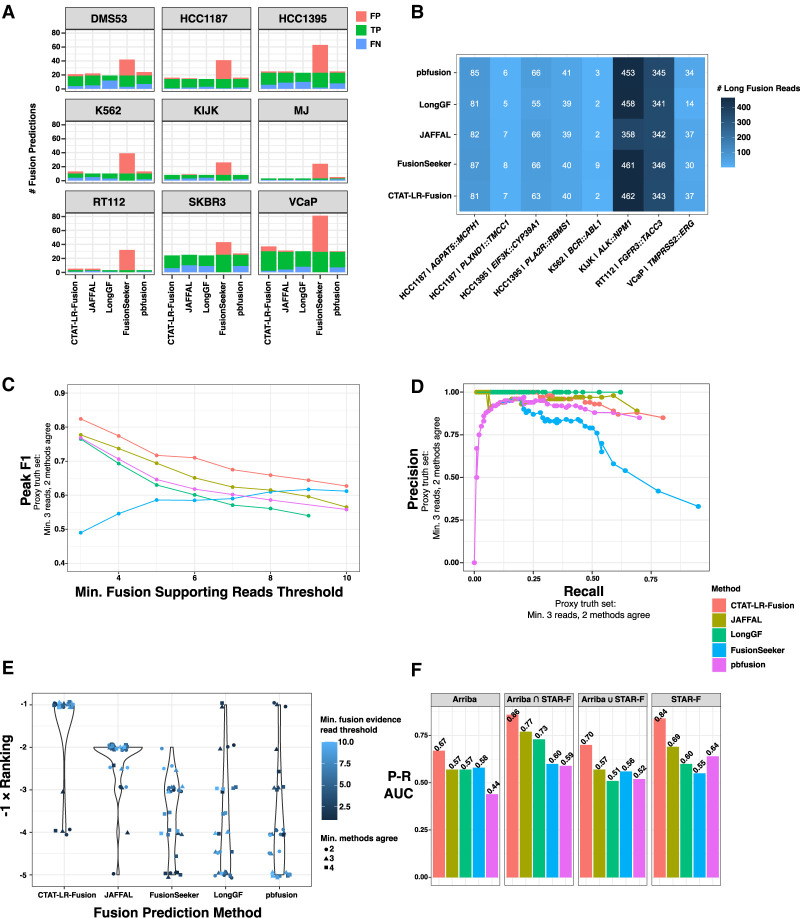
Detection of fusion transcripts from MAS-ISO-seq of nine cancer cell lines. (*A*) Counts of fusion predictions according to cell line, prediction method, and benchmarking class assignment requiring a minimum of three long reads as supporting evidence and a minimum of two different methods agreeing on fusion predictions (referred to as the *example proxy truth set*). Counts of true positives (TPs), FPs, and false negatives (FNs) are shown based on this *example proxy truth set*. Total predicted fusions with minimum three reads support = TP + FP. (*B*) Numbers of MAS-ISO-seq reads identified as evidence for COSMIC fusions according to method. (*C*) Fusion transcript detection accuracy according to minimum long reads supporting evidence, and (*D*) precision versus recall plotted for methods all according to the *example proxy truth set*. Related plots based on each of the proxy truth sets evaluated are available through Supplemental File 2. (*E*) Rankings according to top benchmarking AUC for each method according to differently defined truth sets based on minimum fusion evidence read support followed by a minimum number of agreeing methods. (*F*) Precision–recall AUC for benchmarking methods according to truth sets based on Illumina short-read-supported fusion transcripts.

We further explored long-read-based fusion transcript detection using transcriptomes from nine cancer cell lines derived from diverse cancer types including breast cancer (SKBR3, HCC1187, HCC1395), prostate cancer (VCaP), CML (K562), ALK^+^ anaplastic large cell lymphoma (KIJK), T cell lymphoma (MJ), small cell lung cancer (DMS53), and urothelial bladder cancer (RT112). Several of these cell lines are known to harbor oncogenic fusions including *BCR::ABL1* in K562, *TMPRSS2::ERG* in VCaP, *NPM1::ALK* in KIJK, and *FGFR3::TACC1* in RT112. We sequenced the transcriptomes of each cell line using PacBio MAS-ISO-seq (∼3–6 M reads equating to ∼3–6 G of sequenced bases per sample) (Supplemental Table S1) and called fusions using each long-read fusion transcript prediction method (Supplemental Table S3). Counts of fusions predicted by each method vary greatly by cell line and by method; for example, with a minimum threshold of three reads per reported fusion, cell lines MJ and RT112 have the fewest fusion predictions, VCaP has the most, and the FusionSeeker method produced the greatest numbers of fusion predictions across all cell lines (Fig. 4A).

Eight COSMIC fusions with known relevance to cancer biology including the hallmark fusions mentioned above were identified among most (6/9) of the cell lines and identified by at least two prediction methods with similar quantities of reads for each fusion, spanning two orders of magnitude (two reads for K562|*BCR::ABL1* to 462 reads for KIJK|*ALK::NPM1*) (Fig. 4B). In addition to the read counts being similar, there was excellent agreement in the identity of the individual reads reported as evidence by different methods. Certain differences such as the larger number (nine reads) of *BCR::ABL1* fusion reads reported by FusionSeeker as compared to most other methods (two reads) resulted from FusionSeeker allowing as evidence read alignments that fully localized to intronic regions of fusion partners, whereas CTAT-LR-Fusion requires that read alignments at least partially overlap exons of reference genes.

Benchmarking fusion detection accuracy using these cell lines is challenging due to the lack of absolute truth sets, and experimental validations of fusions from these cell lines are not yet comprehensive. To assess accuracy, we employed proxy truth sets using the “wisdom of the crowds” approach (as in Haas et al. 2019), which operates under the assumption that agreed-upon predictions are more likely to be correct than those predicted uniquely. Supporting this assumption is evidence from orthogonal sets of fusion predictions from our sample-matched Illumina RNA-seq (TruSeq with ∼30–50 M paired-end 151 base length reads [∼10–15 Gb] per sample) (Supplemental Table S1). From Illumina-based Arriba (Uhrig et al. 2021) or STAR-Fusion (Haas et al. 2019) predictions, we find enrichment of Illumina read-supported fusion predictions according to minimum long-read support thresholds (Supplemental Fig. S3A,B) and according to the number of different long-read methods agreeing among the long-read fusion predictions (Supplemental Fig. S3C). We operationally defined alternative sets of truth fusions according to agreement across multiple prediction methods after first filtering predictions according to minimum read support thresholds. Specifically, we defined alternative proxy truth sets as those predicted by a variable minimum number of different methods (2, 3, or 4 different methods) at variable minimum read evidence thresholds (range of 1–10 fusion evidence reads), totaling 3 × 10 = 30 different truth sets, each independently evaluated with the fusion detection methods ranked according to relative overall accuracy (via AUC metric). Evaluations were performed under the most lenient constraints: fusion gene ordering and breakpoints were disregarded, paralogous genes were allowed as proxies for each operationally defined truth set of fusion gene pairs, and only uniquely predicted fusions were scored as FPs (see Methods). For example, shown in Figure 4A, C–E results from one of the 30 trials employing a proxy truth set defined by first filtering fusions at a minimum of three long-read evidence threshold and then requiring at least two different methods to agree, with CTAT-LR-Fusion demonstrating the best performance according to F1 score (Fig. 4C) and precision–recall (Fig. 4D). Distributions of accuracy rankings of methods across all 30 trials are shown in Figure 4E, with CTAT-LR-Fusion identified as top-ranked across most proxy truth sets evaluated, particularly those involving proxy truth sets having at least three fusion reads as evidence—and corresponding to those proxy truth sets that are enriched for orthogonal Illumina support (Supplemental Fig. S3B).

We further explored benchmarking the long-read fusion predictors leveraging truth sets based solely on the Illumina-supported fusion predictions and scoring unique fusion predictions without Illumina support as FPs. From the shorter Illumina read-based fusion predictions derived from Arriba (282 fusions) and STAR-Fusion (266 fusions), 92 fusions were predicted in common, with these common fusion predictions having significantly higher fusion read supporting evidence than those uniquely predicted by either method (*P* < 1 × 10^−7^, Wilcoxon rank sum test, one-sided). We explored alternative definitions of orthogonal evidence-based truth sets corresponding to Arriba or STAR-Fusion support separately, their union (456 fusions), or their intersection (92 fusions) (Fig. 4F; Supplemental Fig. S3D–H). CTAT-LR-Fusion demonstrated the highest accuracy in each evaluation (AUC) (Fig. 4F), with JAFFAL showing the second-highest accuracy. While LongGF was not found as a top-performing method in either of these PacBio MAS-ISO-seq fusion isoform benchmarking efforts, it notably has the highest precision of all methods with no FPs detected at a minimum two read evidence thresholds.

### Harnessing the strengths of long- and short-read fusion identification

**Figure 5.**
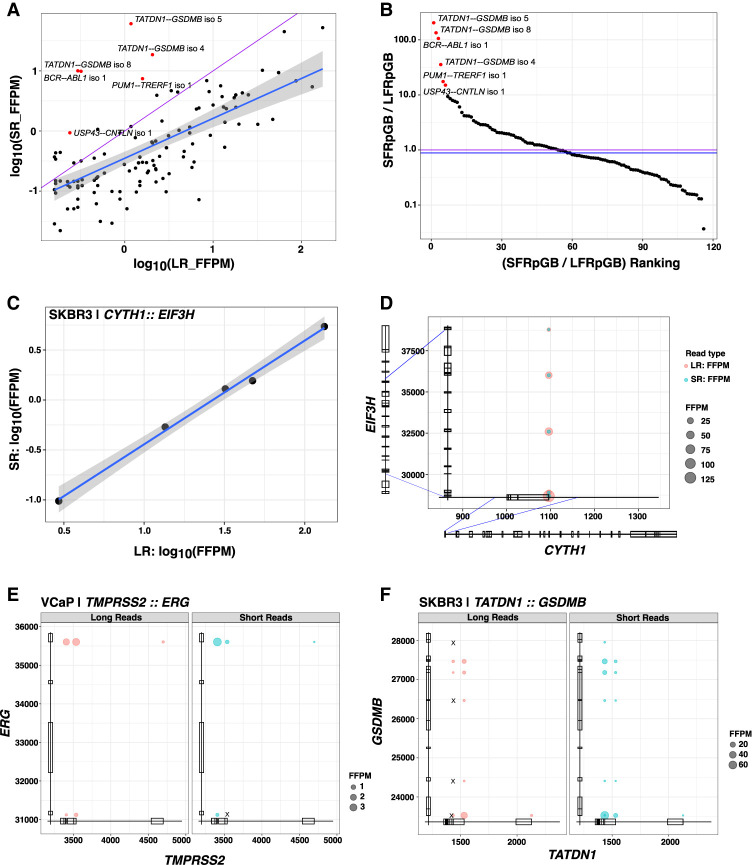
Comparison of long (MAS-ISO-seq) versus short read (TruSeq Illumina) support for fusion isoforms. (*A*) Comparison of fusion isoform read evidence quantification according to CTAT-LR-Fusion (MAS-ISO-seq long reads) and FusionInspector (TruSeq Illumina short reads), with read support normalized for sequencing depth as fusion fragments or reads per total million reads (FFPM). (*B*) Ranking of fusions according to the ratio of Illumina short fusion reads per gigabases sequenced (SFRpGB) to PacBio long fusion reads per gigabases sequenced (LFRpGB). Identity line is shown in purple, and the median ratio is shown in blue. (*C*,*D*) Five fusion isoforms observed for the fusion gene *CYTH1::EIF3H* of cell line SKBR3 are (*C*) observed with highly correlated expression measurements as estimated from long and short RNA-seq reads and (*D*) shown according to fusion transcript breakpoints. (*E*,*F*) Read alignment evidence quantified for fusion isoform breakpoints according to long-read (*left*, red) or short-read (*right*, blue) RNA-seq: (*E*) *TMPRSS2–ERG* fusion in VCaP, (*F*) *TATDN1–GSDMB* fusion in SKBR3. “X” indicates missing read support for the corresponding isoform in the alternative sequencing type.

In exploring the fusion isoforms identified by CTAT-LR-Fusion using combined long and short reads we found 213 fusion genes with 288 fusion splicing isoforms having both short- and long-read alignments together supporting each of the fusion transcript breakpoints. Fusion expression evidence is significantly but moderately correlated between short and long reads (*R* = 0.70, *P* < 2.2 × 10^−16^), and the fraction of fusion-supporting long reads tends to exceed fractions of fusion-supporting short reads (Fig. 5A). However, fusion reads identified according to number of sequenced bases is typically similar, with notable exceptions particularly enriched for short-read fusion evidence support (Fig. 5B; Supplemental Fig. S4A). For example, oncogenic driver fusion *BCR::ABL1* is one notable outlier with >100-fold enrichment of short reads detecting the fusion breakpoint than long reads per Gb sequenced, apparently due to the long length of the fusion transcript with the fusion breakpoint up to 5 kb from the very 3′ end of the fusion transcript and from where PacBio long-read isoform sequencing initiates. Short-read enrichment for fusion detection was observed as weakly but significantly correlated (*R* = 0.27, *P* < 1 × 10^−7^, Pearson's product-moment correlation) with distance from the 3′ end of the fusion transcript (Supplemental Fig. S4B).

Nine fusion genes were found with at least three fusion splicing isoforms each (Supplemental Fig. S5), including *CYTH1::EIF3H* in cell line SKBR3 with five alternatively spliced fusion isoforms with near perfectly positively correlated fusion expression as measured from long or short reads (*R* = 0.997, *P* = 1.9 × 10^−4^) (Fig. 5C,D). The remaining examples mostly involved lowly expressed fusions with weakly- or un-correlated expression as measured according to short- and long-read support (Supplemental Fig. S5). Among these multi-isoform fusions, having access to both long and short reads yielded evidence for fusion isoforms uniquely supported by each read type. For example, *TMPRSS2::ERG* in VCaP has evidence for five fusion splicing isoforms where one is solely supported by long reads (Fig. 5E). In contrast, fusion *TATDN1::GSDMB* in SKBR3 has evidence for 13 fusion splicing isoforms, including four fusion isoforms exclusively supported by short reads (Fig. 5F).

### Fusion detection using ONT isoform sequences

**Figure 6.**
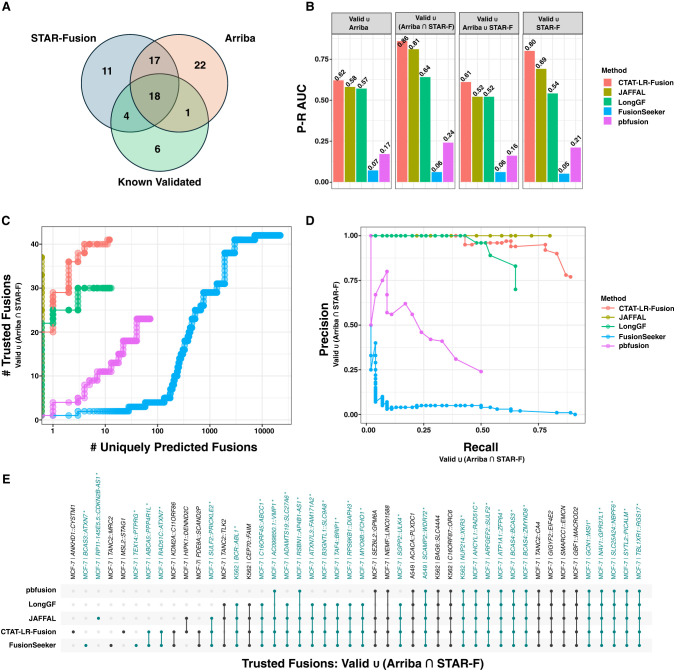
Fusion transcript detection using ONT transcriptome sequences derived from cancer cell lines. (*A*) Counts of trusted fusions identified from long-read isoform sequences according to known validation status and orthogonal Illumina read support as reported by STAR-Fusion or Arriba. (*B*) Fusion detection accuracy for methods according to alternatively defined sets of trusted fusion transcripts as truth sets and scoring uniquely predicted fusions as FPs, with accuracy measured as the P–R AUC. For *C*–*E*, trusted fusions are defined as known validated fusions or those identified by both Arriba and STAR-Fusion as having Illumina read support (see Supplemental Table S6). (*C*) Numbers of trusted fusions detected versus uniquely predicted fusions according to method with fusions ranked from highest to lowest fusion read support. (*D*) Precision–recall plot for benchmarking fusion prediction accuracy using trusted fusions as TPs and uniquely predicted fusions as FPs. (*E*) UpSet plot indicating which trusted fusions were identified by which methods, with trusted fusions colored teal and fusion names with asterisks.

CTAT-LR-Fusion was primarily developed for use with highly accurate PacBio isoform sequencing data but demonstrated potential utility for ONT isoform sequencing data based on the earlier simulations. To further evaluate CTAT-LR-Fusion for fusion detection using ONT, we applied the long-read fusion detection methods to ONT-based cell line transcriptome sequencing derived from the SG-NEx project (Chen et al. 2021). We obtained the ONT direct RNA and cDNA sequencing reads for cell lines A549, K562, and MCF-7 totaling 96 M long reads (65 Gb) across 34 read sets, and corresponding matched Illumina RNA-seq totaling 440 M 151 base paired-end read sequences (133 Gb) across nine read sets (Supplemental Table S1). Fusions were predicted from the ONT sequences using the long-read fusion detection methods and those fusions reproducibly detected by each method across multiple sample replicates (irrespective of direct RNA or cDNA sequencing method) were retained as reproducibly predicted fusions for further evaluation. Illumina RNA-seq was evaluated for fusion transcripts using STAR-Fusion and Arriba, yielding 73 fusions detected with both long and short-read support (Supplemental Table S4). We evaluated identification of trusted fusions in these cell lines (the 73 Illumina-supported fusions combined with 29 known validated fusions, totaling 79 unique trusted fusion gene pairs) (Fig. 6A). In benchmarking fusion detection accuracy leveraging truth sets based on combinations of known validated fusions and those with Illumina support based on STAR-Fusion and/or Arriba predictions, CTAT-LR-Fusion consistently demonstrated highest accuracy (Fig. 6B). For example, when restricting trusted fusions to known validated fusions combined with those agreed-upon by STAR-Fusion and Arriba, CTAT-LR-Fusion maintained high recall on par with FusionSeeker but CTAT-LR-Fusion predicted fewer fusions by more than two orders of magnitude (Fig. 6C–E).

### Long-read fusion isoform detection from tumor single-cell transcriptomes

To examine CTAT-LR-Fusion and long-read isoform sequencing for fusion transcript detection in single cells, we leveraged earlier published PacBio single-cell isoform sequencing data from two recently published studies: a T cell infiltrated melanoma tumor sample from Al'Khafaji et al. (2024), and three different metastatic HGSOC omental samples from Dondi et al. (2023). In both studies, matching sample Illumina RNA-seq data were available, enabling us to further explore differences in the detection of fusion transcripts based on long- versus short-read sequencing. In these single-cell applications, the 10x Genomics single-cell sequencing libraries were based on 3′-end sequencing, inherently biasing sequencing coverage to the very 3′ ends of sequenced isoforms with Illumina RNA-seq.

**Figure 7.**
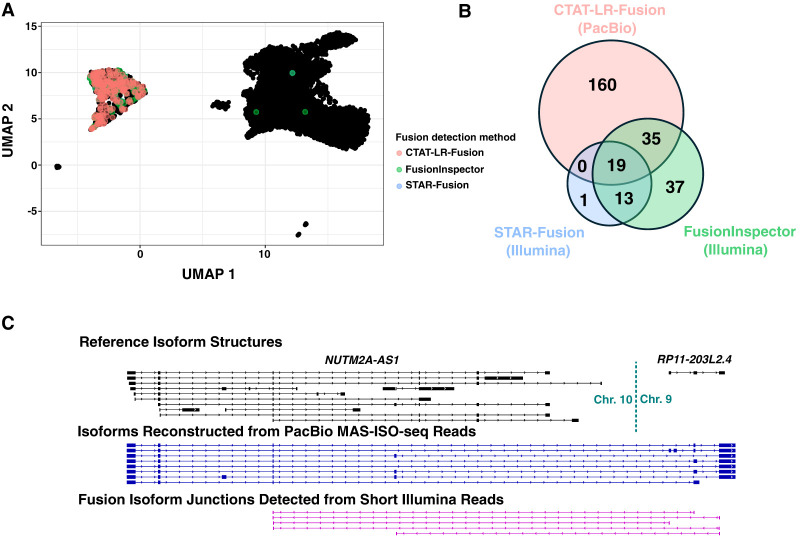
Detection of Fusion *NUTM2A-AS1::RP11-203L2.4* in a T cell infiltrated melanoma tumor sample. MAS-Iso-Seq and matched Illumina RNA-seq data from a melanoma tumor sample M132TS 10× single-cell library (published in Al'Khafaji et al. 2023) were examined for fusion transcripts using CTAT-LR-Fusion for PacBio long reads and STAR-Fusion, FusionInspector, and Arriba for Illumina short reads. (*A*) UMAP for melanoma sample M132TS single cells. Individual cells are shown as (mostly overlapping) black dots. Cells identified with the *NUTM2A-AS1::RP11-203L2.4* fusion transcript are colored according to the detection method, predominantly labeling the cluster of malignant cells. (*B*) Venn diagram indicating the numbers of fusion-containing cells according to detection methods. Fusion *NUTM2A-AS1::RP11-203L2.4* was not reported by Arriba. (*C*) Fusion-supporting transcript isoform structures based on long (*center*) or short (*bottom*) read sequences in the context of the FusionInspector modeled gene fusion contig. GENCODE v22 reference isoform transcript structures for *NUTM2A-AS1* and *RP11-203L2.4* genes are shown at *top*.

The sequenced T cell infiltrated melanoma tumor sample consisted of 6932 cells including 701 tumor cells (10%), sequenced with 21 M PacBio MAS-ISO-seq reads and 207 M single-end 55 base length reads (Supplemental Table S1). Fusion transcripts were examined using CTAT-LR-Fusion for PacBio long reads and STAR-Fusion, FusionInspector, and Arriba for Illumina short reads (Supplemental Table S5). Only one fusion was found in more than 1% of tumor or normal cells: *NUTM2A-AS1::RP11-203L2.4*; notably, fusion gene partner *NUTM2A-AS1* has recently been identified as an oncogene with roles in multiple cancer types (Wang et al. 2020, 2021a; Long et al. 2023). *NUTM2A-AS1::RP11-203L2.4* was found in 265 tumor cells (38%) and only three normal cells (0.05%) through a combination of long- and short-read fusion transcript analyses (Fig. 7A,B); only short-read fusion evidence was found corresponding to these three normal cells, all three detected by FusionInspector and one by STAR-Fusion, and such reads might have derived from ambient tumor RNA. A further search of the long-read sequences for each of the five fusion isoform breakpoint sequences using the text matching utility “grep” (as in Panagopoulos et al. 2014) only identified tumor cells and no normal cells, consistent with long-read fusion support for only tumor cells expressing the *NUTM2A-AS1::RP11-203L2.4*. Approximately 60% of the *NUTM2A-AS1::RP11-203L2.4* containing tumor cells were solely identified by long-read evidence, another 20% by short reads only, and the remaining 20% by both short and long reads (Fig. 7B). The long fusion reads appear to be largely full-length and yield evidence for eight different fusion splicing isoforms, mostly involving skipping of alternative exons and one isoform involving an alternative terminal exon (Fig. 7C). The short-read alignments provide evidence for five alternatively spliced isoform breakpoints but because of the short-read length only the partial isoform structures around the fusion transcript breakpoints were resolved as opposed to the complete isoform structures clearly evident from the long reads (Fig. 7C).

We explored the PacBio long isoform reads and Illumina short reads available for three HGSOC patient samples sequenced at single-cell resolution. Here, tumor samples were derived from omental metastases, and for patient-1 and patient-3, matched normal omentum samples were available and similarly processed and analyzed for comparison (all fusion predictions available as Supplemental Table S6). Numbers of PacBio long reads ranged from 22 to 54 M reads (18–48 Gb) (Supplemental Table S1) along with matched 35–102 M Illumina 56 base length single-end reads (2–6 Gb) (Supplemental Table S1). In addition to identifying previously described fusions for these samples, we identified additional fusion genes and fusion isoforms supported by long and/or short RNA-seq reads, with multiple different fusion gene products generated from the same genome restructuring events. For detecting somatic cancer-specific fusions in these samples, we required at least five tumor cells to exhibit long- or short-read RNA-seq alignment evidence and required identified fusions to be missing from matched normal samples where available.

**Figure 8.**
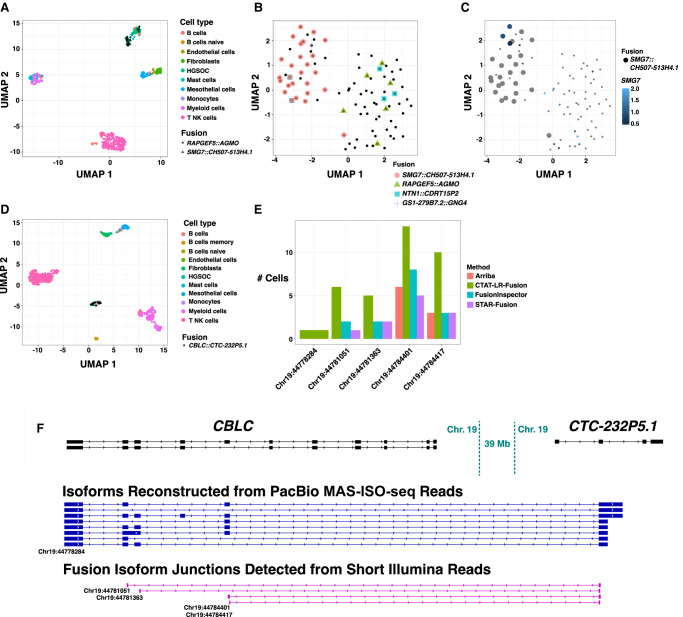
Fusion expression intratumor heterogeneity observed in high-grade serous ovarian cancer cells. (*A*) UMAP embedding of all cells from HGSOC patient-1, colored by cell type. Fusion *RAPGEF5::AGMO* and *SMG7::CH507-513H4.1* are expressed in two different HGSOC cell clusters. Fusion-containing cells are shown as black points with shape according to fusion gene pairs. (*B*) UMAP embedding of HGSOC cells from HGSOC patient-1, colored by fusions expressed. *RAPGEF5::AGMO* is expressed exclusively in the *right* cluster. *SMG7::CH507-513H4.1* and *GS1-279B7.2::GNG4* fusions coexpress and are expressed almost exclusively in the *left* cluster. The two *NTN1::CDRT15P2* fusion-expressing cells in the *left* cluster coexpress the *SMG7::CH507-513H4.1* fusion. (*C*) Tumor cells are colored according to *SMG7* expression and cells identified with fusion *SMG7::CH507-513H4.1* are shown as large dots. Several cells are found to robustly express *SMG7* that do not have evidence of expressing the fusion transcript. (*D*) UMAP embedding for all cells from HGSOC patient-3, colored by cell type. Cells expressing fusion *CBLC::CTC-232P5.1* are shown as black dots. (*E*) Numbers of cells found to express each of the five alternative splicing fusion breakpoints for fusion *CBLC::CTC-232P5.1* according to long- or short-read support and corresponding search method, with *CBLC* fusion isoform breakpoints labeled according to GRCh38 coordinates. (*F*) Fusion-supporting transcript isoform structures based on long-read (*center*) or short-read (*bottom*) sequences in the context of the FusionInspector modeled gene fusion contig. GENCODE v22 reference isoform transcript structures for *CBLC* and *CTC-232P5.1* genes are shown at *top*. *CBLC* fusion isoform breakpoints corresponding to (*E*) are labeled accordingly.

Sequencing of the patient-1 tumor sample yielded 497 total cells, with 92 cells (19%) identified as HGSOC cells, from which we identified only four somatic fusion transcripts: *SMG7::CH507-513H4.1* (26 cells), *RAPGEF5::AGMO* (6 cells), *NTN1::CDRT15P2* (five cells), and *GS1-279B7.2::GNG4* (five cells) (Fig. 8A,B; Supplemental Table S7). For *RAPGEF5::AGMO*, half (3/6) of the cells were detected only by long reads, and 1/6 exclusively by short reads. The other three fusions were found only by long reads. Expression-based clustering of cells for the patient-1 tumor sample resolved two HGSOC cell clusters, with fusion *RAPGEF5::AGMO* evident in tumor cells largely clustered separately from cells expressing *SMG7::CH507-513H4.1* and *GS1-279B7.2::GNG4*, potentially reflecting tumor heterogeneity (Fig. 8A,B). Notably, *SMG7* was detected as robustly expressed in cells across both cell clusters but the *SMG7::CH507-513H4.1* fusion was detected in one cluster (Fig. 8C), supporting heterogeneity in the genome rather than gene expression differences. More rigorous characterization of this tumor sample that further leveraged somatic mutations and copy number variations revealed tumor heterogeneity consisting of two subclones with the *SMG7::CH507-513H4.1* fusion as one of the defining subclonal characteristics (Dondi et al. 2025). Fusion *NTN1::CDRT15P2* was found expressed in both tumor cell clusters and more likely clonal (Fig. 8B).

The patient-2 tumor sample yielded 453 total cells, with 208 (46%) identified as HGSOC cells, from which we identified 17 different malignant cell enriched fusion transcripts (Supplemental Table S7), including the earlier-identified *IGF2BP2::TESPA1* fusion between Chr 3 and Chr 12 evident in 176/208 (85%) of the tumor cells. Another fusion is found with proximal breakpoints yielding fusion transcript *SPATS2::TRA2B* (21 tumor cells, 10%), and likely resulting from the same tumor genome rearrangements involving Chr 3 and Chr 12. Both of these fusions were detected via long and short RNA-seq reads. While a single fusion splicing isoform dominated *IGF2BP2::TESPA1* detection in cells by both long and short reads, four additional fusion splicing isoforms were detected with long- and/or short-read support (Supplemental Table S6). Nearly all (20/21) of the *SPATS2::TRA2B* expression cells are found to coexpress *IGF2BP2::TESPA1*. Other notable fusions in the patient-2 tumor sample involve known tumor oncogenes and include *CBL::KMT2A* (16 tumor cells) and *DEK::CASC17* (11 tumor cells), both identified solely by long reads. The previously reported *FNTA* fusion supported by long reads was missed here but manually verified, as the *FNTA* fusion partner transcribed region was lacking from the reference annotation and currently required for CTAT-LR-Fusion reporting. Another prevalent fusion *PSMB7::SCAI* (52 tumor cells) detected mostly by long reads and with four fusion splicing isoforms involves suppressor of cancer cell invasion gene *SCAI*. The reciprocal fusion *SCAI::PSMB7* was previously detected in serous ovarian cancer cell line COV504_OVARY of the Cancer Cell Line Encyclopedia (Barretina et al. 2012), further implicating this rearrangement as of particular interest to this cancer type.

The patient-3 tumor sample yielded 646 total cells with only 38 (6%) HGSOC cells. Here, only two fusions were identified as enriched in the tumor cells: the previously identified *CBLC::CTC-232P5.1* fusion in 16 cells (Fig. 8D) and our additionally found *SNRNP70::ZIK1* in eight cells (Supplemental Table S7). Each of these *SNRNP70::ZIK1*-expressing cells coexpressed the *CBLC::CTC-232P5.1* fusion. Both fusions involve genes localized to the bottom arm of Chr 19 (*CBLC* and *SNRNP70* transcriptional breakpoints are within 5 Mb), and potentially derive from the same genome restructuring events. There is evidence for five fusion transcript breakpoints for *CBLC::CTC-232P5.1* indicating at least five fusion splicing isoforms, and all but one has support from both short and long reads, with long reads further providing full-length structures for eight different fusion transcript splicing isoforms (Fig. 8E,F). Fusion *SNRNP70::ZIK1* was identified only by long reads.

While in all three of the three HGSOC tumor samples, we found fusions reported only by short reads (Supplemental Table S6), none were found in sufficient numbers of cells (minimum five cells) and enriched among the cancer cell population (at least 80% of occurrences in cancer cells). All candidate oncogenic fusions were observed in these single-cell transcriptomes with long reads or a combination of both long and short reads.

## Discussion

As sequencing technologies and experimental methods continue to advance, we are faced with new challenges and opportunities for the development of computational methods to extract deeper insights and further our understanding of biological systems. Rapid innovation in the long-read sequencing space has enabled full-length single-cell RNA isoform sequencing, pushing the boundaries of transcriptome research. This leap in resolution has transformed our ability to accurately identify, discover, and quantify isoforms from genes and gene fusions, further accelerating biomedical research including studies of cancer and clinical applications to support personalized medicine.

Here, we describe a new addition to our CTAT for the detection of fusion transcripts from long isoform read sequences called CTAT-LR-Fusion. This module complements our earlier-developed CTAT methods available for detecting fusions based on shorter Illumina reads (usually 50–150 bases in length, single-end or paired-end), including TrinityFusion (Haas et al. 2019) for fusion transcripts based on genome-free Trinity (Grabherr et al. 2011; Haas et al. 2013) de novo assembled fusion isoforms, STAR-Fusion (Haas et al. 2019) for fusion detection based on chimeric short-read alignments, and FusionInspector (Haas et al. 2023) for supervised in silico validation of targeted gene fusions. Our CTAT-LR-Fusion method for long isoform read fusion detection was motivated by TrinityFusion, using long isoform reads instead of Trinity-reconstructed transcripts for fusion detection, and by FusionInspector for modeling fusion gene contigs and quantification of fusion read support. FusionInspector is also further integrated into CTAT-LR-Fusion as a submodule for evaluation of Illumina short-read fusion evidence for candidates identified from long reads in case both long and short reads are provided as inputs.

We demonstrated superior accuracy of CTAT-LR-Fusion for fusion detection based on long isoform reads derived from simulated data and from real data as derived from our application of high-throughput PacBio long-read RNA-seq, MAS-ISO-seq, to the Seraseq Fusion RNA Mix v4 control sample containing 16 spiked-in oncogenic fusion transcripts and to nine cancer cell lines. CTAT-LR-Fusion was shown capable of robust identification of all 16 control fusions within the Seraseq fusion mix, and most accurate at identifying fusion transcripts based on simulated data across broad ranges of sequencing error. Furthermore, we demonstrate CTAT-LR-Fusion to be effective at fusion detection using ONT isoform sequencing data derived from cancer cell lines. While high error rates are relegated to the earliest implementations of long-read sequencing technologies, due to continued advancements in sequencing chemistries and computational methods for base-calling, contemporary sequencing accuracies of long reads no longer necessitate fusion detection methods to be compatible with high sequencing error rates. However, as newer long-read sequencing technologies are developed, the more extensive fusion detection capabilities of CTAT-LR-Fusion could prove useful.

The present CTAT-LR-Fusion does have certain limitations, most notably in that it relies on having a reference genome annotation and requires long-read alignments to overlap with known exons of genes, and so chimeric alignments limited to intronic regions of genes or within intergenic regions (novel transcribed regions) are not currently reported. Fusions between pairs of genes that represent paralogous gene pairs are by default filtered as likely artifacts; a feature that can be easily disabled, but based on simulations, none of the methods are particularly adept at identifying fusions between paralogous gene pairs (Supplemental File 2). Furthermore, while we sped up the initial fusion candidate search phase of CTAT-LR-Fusion severalfold through our customized version of minimap2, the entire CTAT-LR-Fusion workflow takes roughly 50% longer (∼90 min per DepMap cell line transcriptome here) than the other methods while using similar compute memory (∼15 GiB RAM) (Supplemental Fig. S6).

There are similar limitations to consider in benchmarking fusion accuracy, both for simulated and for real data. Our simulated fusions all involve reference annotation splice sites for fused exons and fail to account for situations where fusions involve intronic sequences or novel transcripts; the simulated fusions reflect general types of fusions most often encountered as relevant to cancer biology but are far from being inclusive and representing the full diversity of potential fusion transcripts or fusion isoforms. The benchmarks with simulated reads also did not account for background sequences that could contribute toward better evaluating FP rates, and hence almost entirely measured fusion detection accuracy as fusion recall without realistic assessment of precision; hence the especially high FP rate of FusionSeeker on real data was not adequately reflected from its application to simulated reads.

Benchmarking with real sequence data is particularly challenging given the lack of perfect truth sets, and so we resort to proxy truth sets based on criteria of multiple methods agreeing, leveraging minimum fusion read evidence thresholds, and relying on orthogonal sequencing data and algorithms for identifying trusted fusion transcripts as proxy truth sets. In the absence of ground truth, we derived several alternatively defined sets of consensus transcript fusions to serve as our proxy truth. Note, while consensus sets are useful in demonstrating the capture of high-confidence transcript fusions and reporting on the number of uniquely identified fusion transcripts, this approach cannot perfectly discriminate TPs and FPs.

In most of our benchmarking experiments involving simulated or real data, CTAT-LR-Fusion outperformed the alternative methods. However, CTAT-LR-Fusion is not an oracle for fusion transcript detection based on long reads and should be considered along with other top-performing methods such as JAFFAL. Furthermore, the high precision (albeit relative lack of recall) observed for LongGF with bulk PacBio Iso-Seq data warrants consideration. Similarly, if maximum sensitivity is of interest, FusionSeeker may be of interest, but the high rate of FPs could make subsequent validation efforts overly laborious.

Proper detection and reporting of fusion transcripts requires consideration of the order and orientation of the fused genes in the context of the expressed fusion transcripts and accurate reporting of the fusion transcript breakpoint, which most often involves standard transcript splicing that fuses an exon of one gene to an exon of the fusion partner. Of the evaluated long-read isoform fusion detection methods, only CTAT-LR-Fusion and JAFFAL reported fusion gene pairs in their proper order and orientations along with precisely defined fusion isoform breakpoints. Reporting of fusion gene order and orientation is essential, as the alternate fusions made possible between two fusion genes have different interpretations and ramifications regarding oncogenicity, particularly in relation to clinical applications. For example, genes *TACC3* and *FGFR3* neighbor each other within a 100 kb region on Chr 4. A fusion detected as *TACC3::FGFR3* could be considered an example of *cis*-splicing between neighboring genes, and potentially discarded as irrelevant. However, a genome rearrangement yielding the oncogenic fusion *FGFR3::TACC3* (Costa et al. 2016) would be imperative to report. Other scenarios where fusion order and orientation are important considerations include reciprocal translocations, such as frequently encountered for the oncogenic *BCR::ABL1* fusion among others (Haas et al. 2023). Finding *BCR::ABL1* and its reciprocal *ABL1::BCR* fusions in the same patient sample via their distinct fusion transcripts could be considered evidence for a reciprocal chromosome translocation event. Note that in this case the *BCR::ABL1* fusion transcript is the variant that yields the oncogenic fusion protein that drives tumorigenesis, and *ABL1::BCR* is likely collateral damage with questionable relevance to disease.

Accurate detection of fusion transcript breakpoints is essential for characterizing the splicing complexity of gene fusions. It is often the case that gene fusions produce multiple fusion transcript isoforms. For example, for fusion *TATDN1::GSDMB* in breast cancer cell line SKBR3, we find evidence of 13 distinct fusion transcript isoforms. Alternative splicing of fusion genes in cancer provides additional opportunities for neoantigen candidate discovery in applications of personalized immunotherapy, and their consideration could be especially useful when exploring cancers with low tumor mutation burden that otherwise have limited candidates for neoantigen discovery based on expressed and translated somatic variants. There is much interest in leveraging fusions as a source of neoantigens in the development of cancer immunotherapies and tumor vaccines (Yang et al. 2019; Wang et al. 2021b), and early studies are encouraging. For example, immunotherapeutic targeting of the hallmark *DNAJB1-PRKACA* fusion of fibrolamellar hepatocellular carcinoma appears particularly promising based on early findings of a patient with relapse-free survival (Bauer et al. 2022) coupled to related clinical trials underway.

In all our applications of CTAT-LR-Fusion to bulk and single-cell transcriptomes presented here, we examined the capabilities of both long and short RNA-seq reads with matched samples. With few exceptions, fusion detection from long isoform reads greatly outperformed short reads, with more fusion genes and fusion transcript splicing isoforms and greater numbers of tumor single cells expressing fusions detected via long isoform reads. Fusion evidence is more concentrated among the long reads due to the sheer length of each long read, often providing full-length isoform sequences for fused and normal isoforms of transcribed genes, as opposed to Illumina RNA-seq which entails fragmentation of long isoforms into shorter sequenceable fragments of transcripts, with fusion evidence restricted to the sequenced fragments of expressed transcripts. For single-cell transcriptomes, the disparity between long and short reads widens as both long and short reads tend to be biased toward transcript termini depending on 5′ or 3′ anchored chemistry. Detection of fusion isoforms based on short 3′ end sequences poses inherently strict limitations on short reads toward detecting breakpoints that occur proximal to the very 3′ end of the downstream fusion partner. In our survey of a melanoma tumor sample with single-cell transcriptome data, long reads greatly outperformed short reads for detecting potentially oncogenic and tumor-specific *NUTM2A-AS1::RP11-203L2.4* fusion-expressing cells. In our exploration of HGSOC tumor sample transcriptomes at single-cell resolution, we mostly detected tumor-relevant fusions with long isoform reads.

Through the combined use of short and long reads data, we increase the detection sensitivity of gene fusions and numbers of cells with evidence of expressed fusions, demonstrating the synergy of both data types in bulk and single-cell samples. In bulk isoform sequencing, fractions of reads corresponding to fusion isoforms by long and short reads were significantly positively correlated, with specific examples such as SKBR3|*CYTH1::EIF3H* demonstrating near-perfect correlation. Exceptions do exist where long or short reads were found to exclusively detect specific fusion isoforms or contrasting enrichments in the detection of isoforms such that the dominant fusion splicing isoform detected via short reads was not always the dominant fusion isoform detected via long reads. Some differences such as the high enrichment of K562|*BCR::ABL1* fusion detection from short reads can be partially attributed to transcript breakpoints distal from the 3′ end and requiring very long isoform read sequencing to be able to traverse the breakpoint with long reads. Other differences are not yet understood and may reflect sequencing biases between platforms or sequencing protocols. As long-read isoform sequencing becomes more routine, and as we explore increasing numbers of tumor cell lines and tumor single-cell samples, we will have more opportunities to explore these differences, further optimize long-read sequencing methods, and continue to evaluate our toolkit and capabilities for integrated long and short RNA-seq along the way.

## Methods

### CTAT-LR-Fusion long-read fusion isoform detection

The CTAT-LR-Fusion workflow has two phases: (1) initial rapid detection of fusion gene candidates and (2) fusion contig modeling with fusion candidate read alignment and breakpoint support quantification. These phases are described in detail below:

#### CTAT-LR-Fusion phase-1

Rapid detection of fusion gene candidates. Long isoform reads are aligned to the human reference genome using a customized version of minimap2 called ctat-minimap2 (https://github.com/TrinityCTAT/ctat-minimap2), which generates full read alignments only for reads that have preliminary mappings to multiple genomic regions. As most long reads are nonchimeric and mapped to single genomic regions, ctat-minimap2 avoids computational effort in generating alignments for reads that are unlikely to correspond to fusion genes, speeding up this initial read alignment stage fourfold (see Supplemental File 2). Chimeric read alignments derived from ctat-minimap2 are then assigned to reference gene annotations based on genomic coordinates. A preliminary list of fusion candidates is defined based on proximity to reference gene structures, requiring read alignments to have a default minimum of 70% alignment identity. Chimeric long reads are tallied according to candidate gene pairs and read alignment breakpoints are compared to the nearest neighboring exon boundaries. For all supporting reads, the minimum distance between exon boundaries and read alignment breakpoints is determined, and candidate fusion gene pairs are pursued if either of the following conditions are met:

- Chimeric alignment boundary minimum distances are within 50 bases of a reference transcript structure exon boundary.
- One chimeric boundary minimum distance is within 50 bases and the other is within 1 kb of a reference transcript structure exon boundary, and multiple reads support the fusion between candidate gene pairs.

#### Annotation and filtering of phase-1 fusion candidates

Candidate fusion gene pairs are by default annotated using FusionAnnotator (https://github.com/FusionAnnotator/FusionAnnotator/wiki) leveraging CTAT Human Fusion Lib (v0.3.0) (https://github.com/FusionAnnotator/CTAT_HumanFusionLib/wiki) and filtered similarly as in STAR-Fusion with fusions flagged as “red herrings” excluded; these filtered fusions correspond to fusion transcripts known to occur in normal samples (Greger et al. 2014; Babiceanu et al. 2016; Haas et al. 2019, 2023), or involving known conjoined genes (Prakash et al. 2010), duplicated genes (Ouedraogo et al. 2012), or members of gene families (Gray et al. 2016). Candidates involving pairs of genes with overlapping coordinates in the reference genome are further excluded; notably, these can derive from ONT duplex reads as detected in the SG-NEx direct cDNA sequences (see Supplemental File 2). Fusion gene pair candidates are further filtered according to minimum expression threshold criteria (default: minimum 0.1 FFPM = at least 1 fusion long read per 10 M total long reads). The remaining fusion candidates are pursued in CTAT-LR-Fusion phase-2 for further vetting and breakpoint quantification.

#### CTAT-LR-Fusion phase-2: fusion contig modeling, long-read realignment, and breakpoint quantification

Phase-2 leverages techniques and methods in FusionInspector with modifications for long-read alignment. Contig models for fusion genes are constructed using utilities in FusionInspector as previously described (Haas et al. 2023), positioning fusion gene structure candidates in the proposed order and orientation in single contigs with intronic regions shrunken to 1 kb. Candidate fusion-supporting long reads identified in phase-1 are realigned to these fusion contigs using minimap2 (Li 2018). Read alignments with segments that terminate within three bases of a reference transcript exon boundary are snapped to that exon boundary, found useful for highly divergent read alignments, and largely unnecessary for current HiFi reads. Fusion reads are identified as those that align across both genes of fusion contigs with cumulative exon alignment length of at least 25 bases for each paired gene and require that these exons lack sequence similarity between the two candidate fusion genes. Sequence similarities between reference exon annotations were derived from an all-vs-all BLASTN search with results precomputed in the CTAT genome lib as previously described (Haas et al. 2019). Breakpoints for fusion-supporting reads are tallied according to alignment ends that bridge the two genes.

By default, fusions are filtered again based on phase-2 quantification results requiring the minimum of 0.1 FFPM fusion expression evidence. Further, by default, a minimum of one read is required for reporting a fusion involving breakpoints at consensus dinucleotide splice sites, and a minimum of two reads are required as fusion evidence where nonconsensus splice dinucleotides exist at fusion breakpoints; If evidence exists for multiple fusion splicing isoforms for a given fusion gene, those isoforms with <5% of the dominant isoform expression are discarded as potential noise.

When long reads are supplemented with Illumina short reads, FusionInspector is executed with the short reads and the fusion contig gene models derived from CTAT-LR-Fusion phase-1. The FusionInspector results are then merged with the CTAT-LR-Fusion results based on long reads. In this case, the filtering of fusion candidates is modified to consider results based on the short reads such that all fusion isoforms with a minimum of 0.1 FFPM as computed separately from long reads or short reads are included in the final report.

Fusion results based on single-cell transcriptomes are further processed to evaluate per-cell fusion read support. Before running single-cell transcriptome long or short reads through CTAT-LR-Fusion, we encoded cell barcodes and read UMI data into the read name. The fusion reports from CTAT-LR-Fusion and other CTAT fusion modules include lists of reads that support each fusion transcript isoform. From the read names in the fusion reports, we then extract the cell barcodes and UMIs and provide the per-cell reporting of fusion content.

### Fusion isoform detection via long-read or short-read sequencing

For each of the long-read isoform sequencing-based fusion prediction methods, we created Docker images with the most recently available software versions installed. Workflows were built using WDL and data were processed using the Terra cloud computing framework. Software versions used are as follows: CTAT-LR-Fusion (v1.0.0) which we made available at GitHub (https://github.com/TrinityCTAT/CTAT-LR-Fusion), JAFFAL (v2.3) (https://github.com/Oshlack/JAFFA), pbfusion (v0.4.0) (https://github.com/PacificBiosciences/pbfusion/releases), FusionSeeker (v1.0.1 commit 5710dc4; https://github.com/Maggi-Chen/FusionSeeker), and LongGF (v0.1.2) (https://github.com/WGLab/LongGF). Docker files and WDL workflows are available at GitHub (https://github.com/broadinstitute/CTAT-LRF-Paper/tree/main/0.Workflows_and_Dockers); the workflow “0.Workflows_and_Dockers/wdl/long_comb_fusion.wdl” was used to run all long-read fusion methods with consistent alignment parameters, software versions, and genomic resources where applicable. LongGF and FusionSeeker were provided with minimap2 aligned BAM files as input, leveraging splicing parameters “-ax splice:hq -uf” for MAS-ISO-seq reads, and “-ax splice” for divergent (≥5% sequencing error) simulated reads. We prepared the reference data for each of the software based on its tutorial, consistently used GRCh38 as the reference genome, and used GENCODE (Frankish et al. 2019) annotation version 22 for the reference transcriptome annotation. Illumina RNA-seq was analyzed using STAR-Fusion v2.12.0 and FusionInspector v2.8.0 as previously described (Haas et al. 2023). Illumina RNA-seq was further analyzed using Arriba v2.4.0 using the provided Docker image according to the software guide as follows: “docker run ‐‐rm -it -v `pwd`/ArribaReferences:/references -v `pwd`/input_reads1.fastq.gz:/read1.fastq.gz `pwd`/input_reads2.fastq.gz:/read2.fastq.gz -v `pwd`/arriba_output:/output uhrigs/arriba:2.4.0 arriba.sh”.

### Simulated RNA-seq

Simulated fusion isoform reads were obtained from two sources: the JAFFAL published simulated data containing high error rates leveraging Badread (Wick 2019), and our own simulated high-fidelity reads using PBSIM3 (Ono et al. 2022).

#### Badread simulated fusion reads from the JAFFAL publication

We used the JAFFAL study (Davidson et al. 2022) simulated data for ONT and PacBio across the range of sequence divergences (75% identity to 95% identity), which were downloaded from https://ndownloader.figshare.com/files/27676470. These simulated reads were based on the set of 2500 simulated fusion transcripts sequences FASTA files generated in Haas et al. (2019) for five different tissues (https://zenodo.org/records/13354907/files/simulated_fusion_transcript_sequences.tar.gz?download=1): adipose, brain, colon, heart, testis.

#### PBSIM3 simulated fusion reads

To reflect the error profiles of the latest PacBio and ONT sequencing technologies, we also simulated new ONT and PacBio long reads from this same set of 2500 simulated fusion transcripts (500 fusions × 5 simulated fusion sets) using the long-read simulator PBSIM3 v3.0.1 (Ono et al. 2022) at 5× or 50× coverage as follows. To simulate PacBio HiFi reads, we first used PBSIM3 in full-length template-based mode (‐‐strategy templ) with the provided PacBio Sequel continuous long reads (CLR) error model (‐‐errhmm data/ERRHMM-SEQUEL.model) to generate multipass CLR sequencing data, producing 20 passes (‐‐pass-num 20) for each input template to approximate high-accuracy HiFi reads; and then ran the PacBio CCS program v6.4.0 (https://github.com/PacificBiosciences/ccs) to generate HiFi reads from the multipass sequencing data produced by PBSIM3. To simulate ONT R10.4.1 reads, we similarly used the PBSIM3 full-length template-based simulation mode (‐‐strategy templ) and the recently provided error model trained on R10.4 data (‐‐errhmm data/ERRHMM-ONT-HQ.model) with a mean accuracy of 98% (‐‐accuracy-mean 0.98), as recommended by PBSIM3 authors for ONT R10.4.1 reads (https://github.com/yukiteruono/pbsim3/issues/12). To obtain the desired coverage, we created multiple copies of the initial tissue templates and provided the resulting FASTA file as the “‐‐template” parameter to PBSIM3. To link the reads to the original templates from which they were simulated for benchmarking, we made a small update to the PBSIM3 code in a PBSIM3 fork (https://github.com/MethodsDev/pbsim3) to report the read to template name mapping. Summary accuracy statistics were computed across the five sample sets.

### Benchmarking of fusion transcript detection

#### Benchmarking procedures and accuracy metrics

We assessed the TP, FP, and FN for each fusion detection method by comparing their predictions against the, respectively, defined truth set. To quantify and compare the fusion detection performance, we applied four standard metrics for benchmarking fusion detection:

1. precision = TP/(TP + FP)
2. recall = TP/(TP + FN)
3. F1 = (2 × precision × recall)/(precision + recall)
4. area under the precision–recall curve (AUC)

For fusion genes, we have two modes of benchmarking by defining different levels of proper TPs: strict and “allow reverse.” In strict mode, we compared both of the gene pairs while strictly keeping their predicted gene order *geneA::geneB*, and assessed each fusion by matching both pairs of the genes with their official gene symbols, gene symbols for paralogs, and genes with overlapping coordinates along the genome. In “allow reverse” mode, we allowed the predicted gene order to be *geneA::geneB* or *geneB::geneA* when comparing with the corresponding truth set. For both *geneA* and *geneB*, gene symbols for genes with overlapping genomic coordinates or paralogs of respective genes were allowed as proxies and scored equivalently.

For breakpoint comparisons, we also implemented fuzzy or exact modes of performing the benchmarking. The two breakpoints were first sorted to ignore breakpoint ordering before comparison in either fuzzy or exact breakpoint evaluation modes. In exact mode, we strictly compared the sorted two breakpoint genomic coordinates for identity, and in fuzzy mode, we expanded the allowed breakpoints of a fusion event to a window encompassing five bases upstream and downstream from each breakpoint.

Precision–recall plots were computed by evaluating precision and recall metrics according to minimum fusion isoform evidence read count thresholds. The P–R AUC was computed and reported as an overall accuracy metric (Davis and Goadrich 2006).

#### Consistent filtering of fusions from cancer cell line transcriptomes

We excluded fusions that tend to be enriched for artifacts, commonly encountered from normal samples, or likely resulting from *cis*-splicing of neighboring transcripts; specifically, we filtered fusions including mitochondrial genes, *HLA* genes, gene pairs involving immunoglobulin gene rearrangements, fusions involving neighboring genes within 100 kb on a chromosome, or any fusions annotated as previously found in normal samples according to FusionAnnotator. Fusions passing these criteria were further filtered to retain fusions most relevant to individual cell lines by excluding fusions that involved promiscuous genes defined as those genes reported in fusion predictions by multiple different methods across the multiple different cell lines examined here. All filtered fusions are henceforth collectively operationally referred to as “nuisance fusions.”

#### “Wisdom of the crowds” benchmarking of cancer cell lines

When benchmarking using bulk cancer cell lines MAS-ISO-seq data and the “wisdom of crowds” approach toward defining proxy truth sets, we first filtered all the methods’ fusion calls based on a specified minimum long reads support (range of 1–10 minimum reads), and then identified sets of fusions agreed upon by 2, 3, or 4 different methods, yielding 30 different proxy truth sets. After first filtering fusions according to minimum read evidence thresholds and before defining proxy truth sets and performing benchmarking, we filtered out nuisance fusions as described above. We then defined proxy truth set (TPs) as those fusions predicted by the required minimum number of different methods, and defined proxy FPs as fusions uniquely predicted by the corresponding method. Precision, recall, F1 metrics, precision–recall plots, and AUC were computed using each corresponding truth set, applying lenient criteria ignoring gene pair ordering, and allowing for paralogous proxy gene representation.

#### Benchmarking using trusted fusions

Trusted fusions used for benchmarking long-read fusion detection were defined as known earlier-validated fusions or separately supported by orthogonal Illumina RNA-seq as indicated. Fusion predictions from all methods were first filtered from nuisance fusions as described above. Truth set fusions were defined as fusions predicted by long reads that are included in the set of trusted fusions. FP fusion predictions were limited to those long-read fusions uniquely predicted by a corresponding method.

#### Benchmarking of reproducibly predicted fusions from the ONT SG-NEx data set

ONT isoform sequences for tumor cell lines were obtained from SG-NEx: The Singapore Nanopore Expression Project https://github.com/GoekeLab/sg-nex-data (Chen et al. 2021). Fusion predictions for each method were first filtered to retain only those fusion gene pairs that were predicted in at least two sample replicates for each cell line separately according to each prediction method, irrespective of direct RNA or cDNA sequencing method used, read support, or breakpoint reported. After restricting fusions to these replicated gene pairings, fusion read support was then aggregated across all sample replicates and sequencing runs according to cell line, fusion gene pair, and fusion isoform breakpoints. The reproducibly detected fusions were then filtered of nuisance fusions as described above and then benchmarked using sets of trusted fusions (see section above “Benchmarking using trusted fusions”). The sample-matched Illumina SG-NEx RNA-seq samples (Supplemental Table 1) were evaluated for fusion content using STAR-Fusion and Arriba. Our K562 Illumina RNA-seq data generated here were leveraged to supplement the SG-NEx K562 sample-matched Illumina (results included in Supplemental Table 4 as “other_illumina”).

A small fraction of pbfusion v0.4.0 results (∼1%) involved complex fusions involving multiple partners that were not always clearly identified with breakpoint information. For benchmarking purposes, we ignored instances where there lacked a clear one-to-one mapping between breakpoint coordinates and fusion partners. In the evaluation of the SeraCare fusions, the pbfusion output was manually examined to confirm the capture of a reference fusion where breakpoint information was not clearly defined.

#### Fusion detection in single-cell transcriptomes

The human T cell infiltrating melanoma single-cell RNA-seq data examined here and previously published in Al'Khafaji et al. (2024) are available from the database of Genotypes and Phenotypes (dbGaP; https://www.ncbi.nlm.nih.gov/gap/) under accession number phs003200.v1.p1. The HGSOC single-cell data were obtained from the European Genome-phenome Archive (EGA; https://ega-archive.org) study EGAS00001006807 as dataset IDs EGAD00001009814 (PacBio) and EGAD00001009815 (Illumina). FASTQ files for long- and short-read RNA-seq were input to fusion transcript detection methods with read names formatted to incorporate cell barcodes and UMIs. The cell barcodes were subsequently extracted from fusion evidence read names to identify cells with evidence of fusion transcript expression. Single-cell data were further analyzed using Seurat (Supplemental File 2; Satija et al. 2015).

### Bulk 8-mer MAS-Iso-seq for nine DepMap cell lines and two SeraCare fusion mix v4 replicates

#### RNA QC of cancer cell lines and Seraseq fusion RNA mix

RNA samples were extracted from nine cancer cell lines (VCAP, MJ, K562, RT112, KIJK, HCC1187, HCC1395, DMS53, and SKBR3) using Qiagen's RNeasy Plus Kit (Qiagen 74134), and RNA from the Seraseq Fusion RNA mix v4 (SeraCare 0710-0497) were quality checked using a High Sensitivity RNA ScreenTape (Agilent 5067–5579 and 5067–5580) on an Agilent 4150 TapeStation system (Agilent G2992AA) to determine RNA Integrity Number (RIN) before first-strand synthesis (FSS).

#### cDNA synthesis from cancer cell lines and SeraCare fusion RNA mix

For both the cancer cell lines and the Seraseq Fusion RNA mix, cDNA was generated from RNA using components from a NEBNext Single Cell/Low Input cDNA Synthesis & Amplification Module (New England Biolabs E6421S). The RNA Samples were diluted, the cancer cell lines to 50 ng/µL, and the SeraSeq fusion RNA mix to 15 ng/μL. Per sample, the diluted RNA (200 ng/cancer cell line sample, 100 ng/SeraSeq fusion mix) was combined with 3 µL of water, and 2 µL of NEBNext Single cell RT primer (Sequence: AAG CAG TGG TAT CAA CGC AGA GTA CTT TTT TTT TTT TTT TTT TTT TTT TTT TTT TV), mixed via pipetting, and incubated at 70°C for 45 min before cooling to 20°C. Each reaction was then immediately combined with a second reaction mix consisting of 5 µL of NEBNext Single Cell buffer, 2 µL of NEBNext Single Cell RT Enzyme Mix, and 3 µL of Nuclease-free water. The reaction was then incubated at 42°C for 45 min before being removed from the thermal cycler, having 1 µL of 100 µM Template switch oligo (Sequence; GCA ATG AAG TCG CAG GGT TrGrG rG) mixed in via pipetting, returning the reaction mix to the thermal cycler and incubating at 42°C for 15 min, then 85°C for 5 min, holding at 4°C. Thirty microliters of elution buffer was added to each reaction for a total volume of 50 µL, each reaction was then cleaned using 40 µL (0.8× reaction volume) of SPRI beads (Beckman Coulter Inc. B23318) according to the manufacturer's recommendations. The reaction was eluted in 50 µL of elution buffer. Fifteen microliters of each cDNA was taken from the previous elution volume, and then combined with 25 µL of NEBNext Single Cell cDNA PCR Master Mix, 2.5 µL of 5 µM Forward Primer (Sequence: AAG CAG TGG TAT CAA CGC AGA G), 2.5 µL of an index reverse primer (sequence, variable, see Supplemental Table S8) and 5 µL of Nuclease-free Water for a total volume of 50 µL. The reaction was mixed and then incubated in the thermal cycler for one cycle of 3 min at 98°C, 12 cycles of 20 sec at 98°C—30 sec at 62°C—8 min at 72°C, then one cycle of 5 min at 72°C, holding at 4°C. Each reaction was then cleaned using 35 µL (0.7× reaction volume) of SPRI beads. The reaction was eluted off the beads in 50 µL of elution buffer. The samples were quantified using a Qubit Flex Fluorometer (Thermo Fisher Scientific Q33327) and Qubit dsDNA HS Assay kit (Thermo Fisher Scientific Q32854) and analyzed via High Sensitivity D5000 ScreenTape (Agilent 5067–5594, 5067–5593, and 5067–5592) on an Agilent 4150 TapeStation system. The resultant cDNA was diluted down to 5 ng/µL.

#### PacBio SMRTbell library preparation

The following section of the sequencing preparation was completed using kit components from the MAS-Seq for 10x Single Cell 3′ kit (PacBio 102-659-600), as well as individually created oligos. A PCR master mix for each sample was made using 100 µL of MAS PCR Mix, 20 ng of cDNA in 4 µL of volume, and 96 µL of nuclease-free water for a total volume of 200 µL. The master mix was mixed and 22.5 µL aliquots were distributed to each well of a 0.2 mL PCR tube strip (USA Scientific Inc. 1402–2500) where a 2.5 µL addition of a 5 µM primer mix was added (see Supplemental Table S9). The samples were mixed and incubated in the thermal cycler for an initial denaturation step of one cycle for 3 min at 98°C, then seven cycles of denaturation for 20 sec at 98°C, annealing for 30 sec at 68°C, and extension for 8 min at 72°C, finally, a terminal extension of one cycle for 5 min at 72°C, holding at 4°C. After incubation, the entire volume of each strip tube was pooled into a 1.5 mL tube (total volume 200 µL) before a 0.95× SPRI bead clean. The resultant product was eluted into 50 µL of elution buffer. The product was quantified via Qubit Flex Fluorometer. Forty-seven microliters from the previous elution was transferred into a 0.2 mL PCR tube, 10 µL of MAS Enzyme was added to each reaction then pipette mixed. The reactions were then incubated for 30 min at 37°C, holding at 4°C. The reactions were removed, and two reaction mixes were added, the first consisted of 1.5 µL of MAS Adapter A Fwd 1.5 µL of MAS Adapter Q Rev, and 20 µL of MAS Ligation additive. The second reaction mix added consisted of 10 µL of Mas Ligase Buffer, and 10 mL of MAS Ligase for a total combined reaction of 100 µL. The reaction was mixed with wide bore pipette tips (Mettler-Toledo Rainin LLC 30389241), before being incubated for 60 min at 42°C, holding at 4°C. The reactions were removed from the thermal cycler and 75 µL (0.75×) of resuspended SPRI beads were added. The reactions were mixed thoroughly using wide bore pipette tips and then left to incubate at room temperature for 10 min. The reactions were placed on a magnetic strip to pellet the beads, which were then washed twice in 200 µL of 80% ethanol. Forty-five microliters of elution buffer was added to the reactions after the second ethanol wash and was left to elute off the beads for 5 min at room temperature. The reaction was then added back onto the magnet and the 45 µL eluted MAS Array was moved to a separate 0.2 mL PCR tube. Forty-two microliters of each of the eluted MAS array was transferred to a new 0.2 mL PCR tube and a reaction mix consisting of 6 µL of Repair buffer, and 2 µL of DNA Repair Mix, was added for a total volume of 50 µL. The reaction was mixed using wide bore pipette tips before incubating for 30 min at 37°C, holding at 4°C. The reactions were removed from the thermal cycler and 37.5 µL (0.75×) of resuspended SPRI beads were added, and then cleaned according to the manufacturer's specifications. The reaction was eluted in 40 µL of elution buffer. To the 40 µL of eluted DNA, a reaction mix consisting of 5 µL of Nuclease buffer and 5 mL of Nuclease mix was added for a total volume of 50 µL. The reaction was pipette mixed using wide bore pipettes then incubated for 60 min at 37°C, holding at 4°C. The reactions were removed from the thermal cycler and 37.5 µL (0.75×) of resuspended SPRI beads were added. The reactions were mixed thoroughly using wide bore pipette tips and then left to incubate at room temperature for 10 min. The reactions were placed on a magnetic strip to pellet the beads, which were then washed twice in 200 µL of 80% ethanol. Twenty-five microliters of elution buffer was added to the reactions after the second ethanol wash and were left to elute off the beads for 5 min at room temperature. The reaction was then added back onto the magnet and the 25 µL eluted MAS Array was moved to a separate 0.2 mL PCR tube. The reaction was then quantified using a Qubit Flex Fluorometer, and characterized using a Genomic DNA ScreenTape Analysis (Agilent 5067–5366 and 5067–5365) on an Agilent 4150 TapeStation system.

### PacBio monomeric MAS-ISO-seq for SeraCare fusion RNA mix v4

#### RNA QC of Seraseq fusion RNA Mix v4 for monomeric MAS-seq

The RNA sample (Seraseq Fusion RNA Mix v4 0710–0497) was quality checked using a High Sensitivity RNA ScreenTape (Agilent 5067–5579 and 5067–5580) on an Agilent 4150 TapeStation system (Agilent G2992AA) to determine RIN before FSS.

### cDNA synthesis from Seraseq RNA Mix v4 for monomeric MAS-seq

cDNA was generated from RNA using components from a NEBNext Single Cell/Low Input cDNA Synthesis & Amplification Module (New England Biolabs E6421S), MAS-Seq for 10x Single Cell 3′ kit (PacBio 102-659-600), and individually created oligos. The RNA mix was diluted to 10 ng/µL and split into two separate reaction vessels. Per reaction, the diluted RNA (10 ng/µL, 7 µL total volume, 70 ng total) was combined with 2 µL of NEBNext Single Cell RT primer (Sequence: AAG CAG TGG TAT CAA CGC AGA GTA CTT TTT TTT TTT TTT TTT TTT TTT TTT TTT TV), mixed via pipetting, and incubated at 70° C for 45 min before cooling to 20°C. Each reaction was then immediately combined with a second reaction mix consisting of 5 µL of NEBNext Single Cell buffer, 2 µL of NEBNext Single Cell RT Enzyme Mix, and 3 µL of Nuclease-free water. The reaction was then incubated at 42°C for 45 min before being removed from the thermal cycler, having 1 µL of 100 µM Template switch oligo (Sequence; GCA ATG AAG TCG CAG GGT TrGrG rG) mixed in via pipetting, returning the reaction mix to the thermal cycler and incubating at 42°C for 15 min, then 85°C for 5 min, holding at 4°C. Thirty microliters of elution buffer was added to each reaction vessel for a total volume of 50 µL, each reaction was then cleaned using 40 µL (0.8 × reaction volume) of SPRI beads (Beckman Coulter Inc. B23318) according to the manufacturer's recommendations. The reaction was eluted off the beads in 50 µL of elution buffer. Fifteen microliters of each cDNA reaction was aliquoted from the previous elution volume, and then combined with 25 µL of NEBNext Single Cell cDNA PCR Master Mix, 2.5 µL of MAS Capture Primer FWD (Sequence: AAG CAG TGG TAT CAA CGC AGA G), 2.5 µL of MAS Capture Primer REV, and 5 µL of Nuclease-free water for a total volume of 50 µL. The reaction was mixed and then incubated in the thermal cycler for one cycle of 3 min at 98°C, 14 cycles of 20 sec at 98°C—30 sec at 62°C—8 min at 72°C, then one cycle of 5 min at 72°C, holding at 4°C. Each reaction was then cleaned using 35 µL (0.7 × reaction volume) of SPRI beads. The reaction was eluted off the beads in 50 µL of elution buffer. The samples were quantified using a Qubit Flex Fluorometer (Thermo Fisher Scientific Q33327) and Qubit dsDNA HS Assay kit (Thermo Fisher Scientific Q32854) and analyzed via High Sensitivity D5000 ScreenTape (Agilent 5067–5594, 5067–5593, and 5067–5592) on an Agilent 4150 TapeStation system.

#### PacBio SMRTbell library preparation

The following section of the sequencing preparation was completed using kit components from the MAS-Seq for 10x Single Cell 3′ kit (PacBio 102-659-600), as well as individually created oligos. A PCR mix for the sample was made using 25 µL of MAS PCR Mix, 5 ng of cDNA in 2 µL of volume, and 23 µL of nuclease-free water for a total volume of 50 µL. The master mix was mixed and a 45 µL aliquot was distributed to one well of a 0.2 mL PCR tube strip (USA Scientific Inc. 1402–2500) where 5 µL addition of a 5 µM primer mix of primers A-FWD and Q-REV was added (A-FWD, Sequence: AGCTTACTUGTGAAGAUCTACACGACGCTCTTCCGATCT, Q-REV, Sequence: AUGCACACAGCUACUAAGCAGTGGTATCAACGCAGAG). The sample was mixed and incubated in the thermal cycler for an initial denaturation step of one cycle for 3 min at 98°C, then seven cycles of denaturation for 20 sec at 98°C, annealing for 30 sec at 68°C, and extension for 8 min at 72°C, finally, a terminal extension of one cycle for 5 min at 72°C, holding at 4°C. After incubation, 47.5 µL (0.95×) SPRI beads were added for a clean. The resultant product was eluted into 60 µL of elution buffer. The product was quantified via Qubit Flex Fluorometer. Fifty-five microliters was transferred into a 0.2 mL PCR tube, 2 µL of MAS Enzyme was added to each reaction then pipette mixed. The reaction was incubated for 30 min at 37°C, holding at 4°C. The reaction was removed, and two reaction mixes were added, the first consisted of 1.5 µL of MAS Adapter A Fwd 1.5 µL of MAS Adapter Q Rev, and 20 µL of MAS Ligation additive. The second reaction mix added consisted of 10 µL of Mas Ligase Buffer, and 10 mL of MAS Ligase for a total combined reaction of 100 µL. The reaction was mixed with wide bore pipette tips (Mettler-Toledo Rainin LLC 30389241), before being incubated for 60 min at 42°C, holding at 4°C. The reactions were removed from the thermal cycler and 75 µL (0.75×) of resuspended SPRI beads were added and cleaned according to the manufacturer's recommendations. The reaction was eluted in 45 µL of elution buffer 42 µL of the eluted MAS array was transferred to a new 0.2 mL PCR tube and a reaction mix consisting of 6 µL of Repair buffer, and 2 µL of DNA Repair Mix was added for a total volume of 50 µL. The reaction was mixed using wide bore pipette tips before incubating for 30 min at 37°C, holding at 4°C. The reactions were removed from the thermal cycler and 37.5 µL (0.75×) of resuspended SPRI beads were added, and then cleaned according to the manufacturer's recommendations. The reaction was eluted in 40 µL of elution buffer. To the 40 µL of eluted DNA, a reaction mix consisting of 5 µL of Nuclease buffer and 5 mL of Nuclease mix was added for a total volume of 50 µL. The reaction was pipette mixed using wide bore pipettes then incubated for 60 min at 37°C, holding at 4°C. The reactions were removed from the thermal cycler and 37.5 µL (0.75×) of resuspended SPRI beads were added and cleaned according to the manufacturer's recommendations. The reaction was eluted in 25 µL of elution buffer. The final product was then quantified using a Qubit Flex Fluorometer and characterized using a High Sensitivity D5000 ScreenTape on an Agilent 4150 TapeStation system.

#### Illumina TruSeq RNA-seq for nine DepMap cell lines and three SeraCare fusion RNA mix v4 replicates

DepMap samples were quantified by Qubit Ribogreen and normalized to 350 ng inputs, respectively, for the TruSeq stranded RNA protocol. All samples were determined by Agilent Bioanalyzer to have high quality with RINs > 9. Polyadenylated RNAs were selected before fragmentation on the Covaris. Stranded cDNA libraries were generated following the Illumina TruSeq Stranded Total RNA protocol (https://support.illumina.com/content/dam/illumina-support/documents/documentation/chemistry_documentation/samplepreps_truseq/truseq-stranded-total-rna-workflow/truseq-stranded-total-rna-workflow-reference-1000000040499-00.pdf). cDNA libraries incorporating ligated adapters were pooled and loaded on the NovaSeq SP for paired-end 151 bp sequencing targeting 50 M paired reads per sample.

#### Single-cell RNA-seq data

Melanoma sample M132TS—used previously published data from Al'Khafaji et al. (2024). This earlier publication focused on the T cells and here we focused on the tumor cells, and so we extracted both and reprocessed through CellBender (Fleming et al. 2023). HGSOC—used previously published data from Dondi et al. (2023), reads downloaded from the EGA (Freeberg et al. 2022) under accession numbers EGAD00001009814 (PacBio) and EGAD00001009815 (Illumina). Cell annotations and long-read gene counts per cell were retrieved from Dondi et al. For visualization, counts were normalized independently for each patient using sctransform (Hafemeister and Satija 2019), regressing out cell cycle effects and library size as nonregularized dependent variables. Similar cells were grouped using Seurat FindClusters (Satija et al. 2015). The results of cell clustering and cell typing were visualized in a low-dimensional representation using Uniform Manifold Approximation and Projection (UMAP) (McInnes et al. 2018).

## Data access

The PacBio MAS-Iso-Seq and Illumina TruSeq data generated in this study for SeraCare SeraSeq Fusion Mix RNA v4 and the nine DepMap cell lines have been submitted to the NCBI BioProject database (https://www.ncbi.nlm.nih.gov/bioproject/) under accession numbers PRJNA1076207 and PRJNA1077632, respectively. Our PBSIM3 simulated fusion reads are available at Zenodo (https://doi.org/10.5281/zenodo.10650516). CTAT-LR-Fusion is freely available as open-source software at GitHub (https://github.com/TrinityCTAT/CTAT-LR-Fusion/wiki), and v1.0.0 is included as Supplemental File 1. All analyses and figures generated as part of this work (https://github.com/broadinstitute/CTAT-LRF-Paper), benchmarking analysis code, and the raw outputs from each of the evaluated prediction methods (https://github.com/fusiontranscripts/LR-FusionBenchmarking) are packaged as Supplemental File 2.

## Supplemental Material

Supplement 1

Supplement 2

Supplement 3

Supplement 4

Supplement 5

Supplement 6

Supplement 7

Supplement 8

Supplement 9

Supplement 10

Supplement 11

Supplement 12
