# Supplementary material for: Accurate fusion transcript identification from long- and short-read isoform sequencing at bulk or single-cell resolution
Source: Genome Res. 2025 Apr;35(4):967–86. doi: 10.1101/gr.279200.124 (PMC12047241; doi:10.1101/gr.279200.124)
Supplement: Supplement 1 [file Supplemental_Figures.pdf]

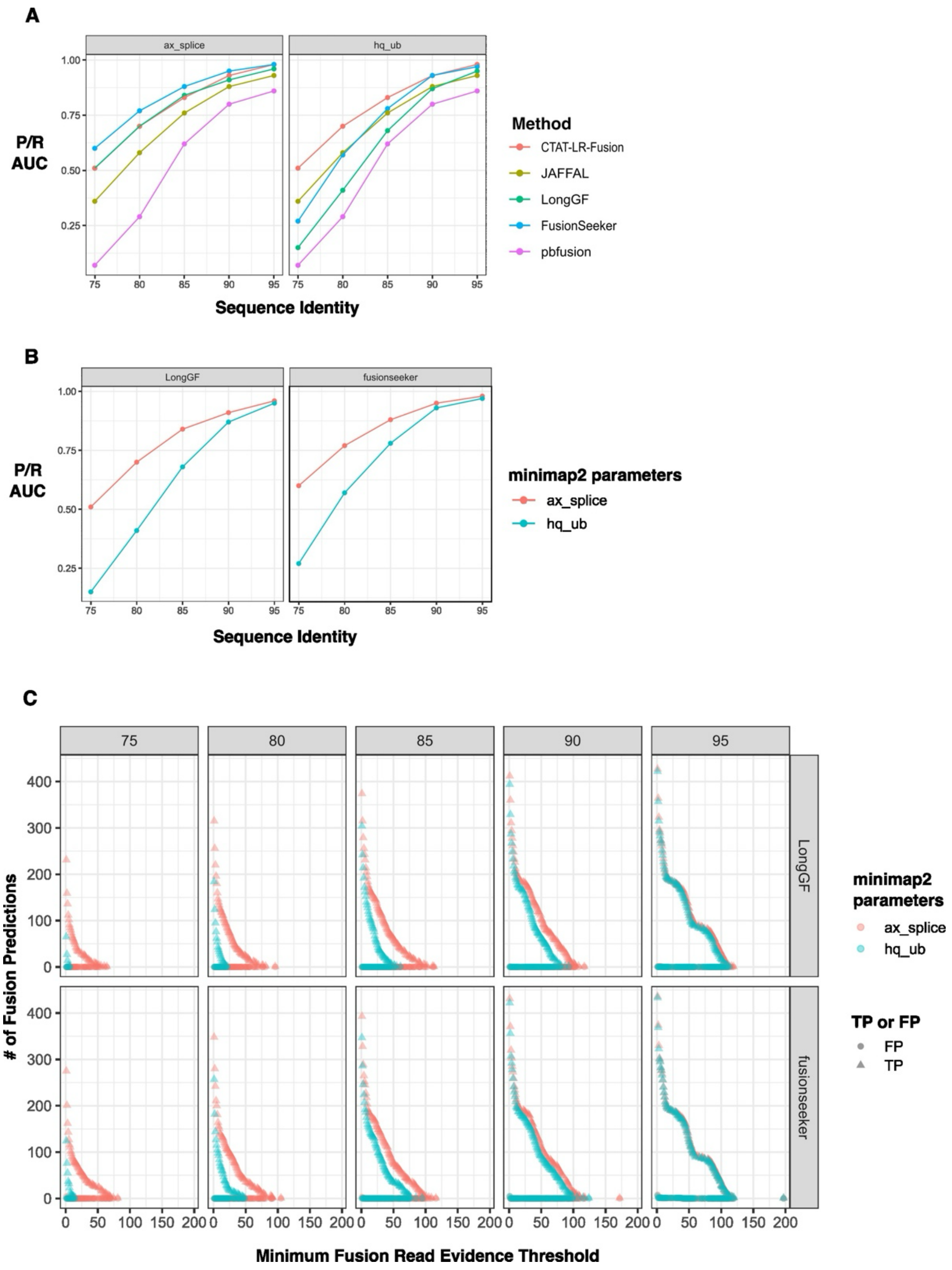


**Supplemental Figure S1. FusionSeeker and LongGF performance improvements with divergent simulated reads and fusion isoform detection using altered minimap2 parameters.** The minimap2 parameters "-ax splice" or "-ax splice:hq -ub" were leveraged to generate aligned bam files input to LongGF or FusionSeeker followed by benchmarking fusion detection accuracy. (A) Comparison of accuracy (AUC of precision/recall plots) according to fusion detection method and minimap2 alignment parameters. Only LongGF and Fusionseeker allow for configurable minimap2 alignments, and accuracy differences are apparent in comparison to the alternative methods. (B) Comparison of the difference in fusion detection accuracy for LongGF and FusionSeeker according to minimap2 settings. (C) Comparison of numbers of true positives (TP) and false positives (FP) for Fusionseeker and LongGF for each divergent read set. Differences in recall are observed below the 95% identity sequence divergence ranges tested, while numbers of FP are largely unaffected.

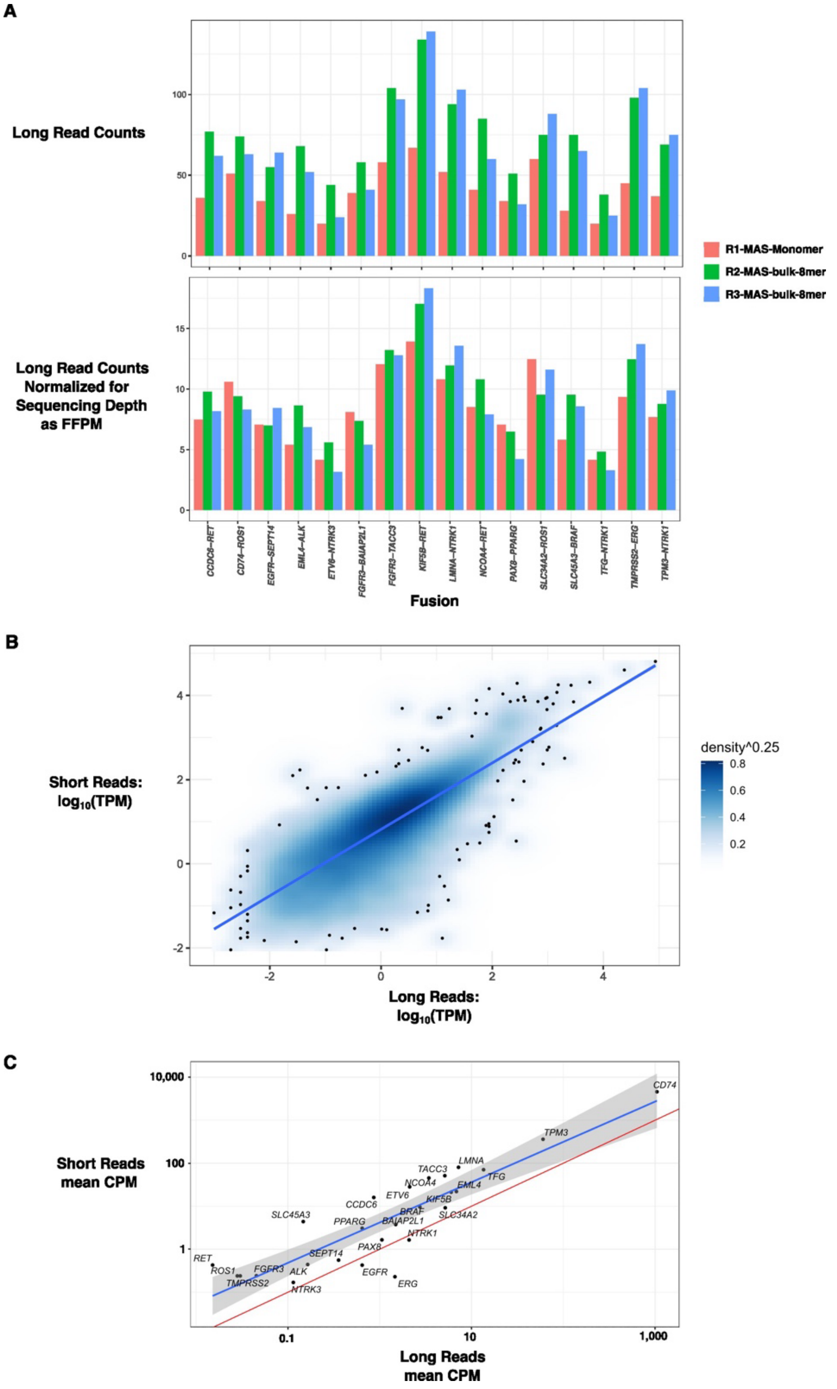


**Supplemental Figure S2: CTAT-LR-fusion detection of 16 control fusion transcripts based on PacBio long read isoform sequencing.** (A,top) raw counts, (A,bottom) normalized for sequencing depth as FFPM. (B) Gene expression quantification comparison based on long reads in comparison to short reads based on quantification of GENCODE v22 reference annotations performed using Salmon. Expression values (TPM measurements) for PacBio long read sequencing and Illumina TruSeq short read sequencing are significantly positively correlated (R=0.79, $p<2.2e-16$). (C) Read support quantification for the individual fusion control genes according to Illumina short reads or PacBio long isoform reads normalized as counts per million (CPM) indicating higher read abundances for short reads as compared to long reads (median 4.5 short reads per long read support); identity line shown as red and linear regression line in blue with gray representing the 95% confidence level.

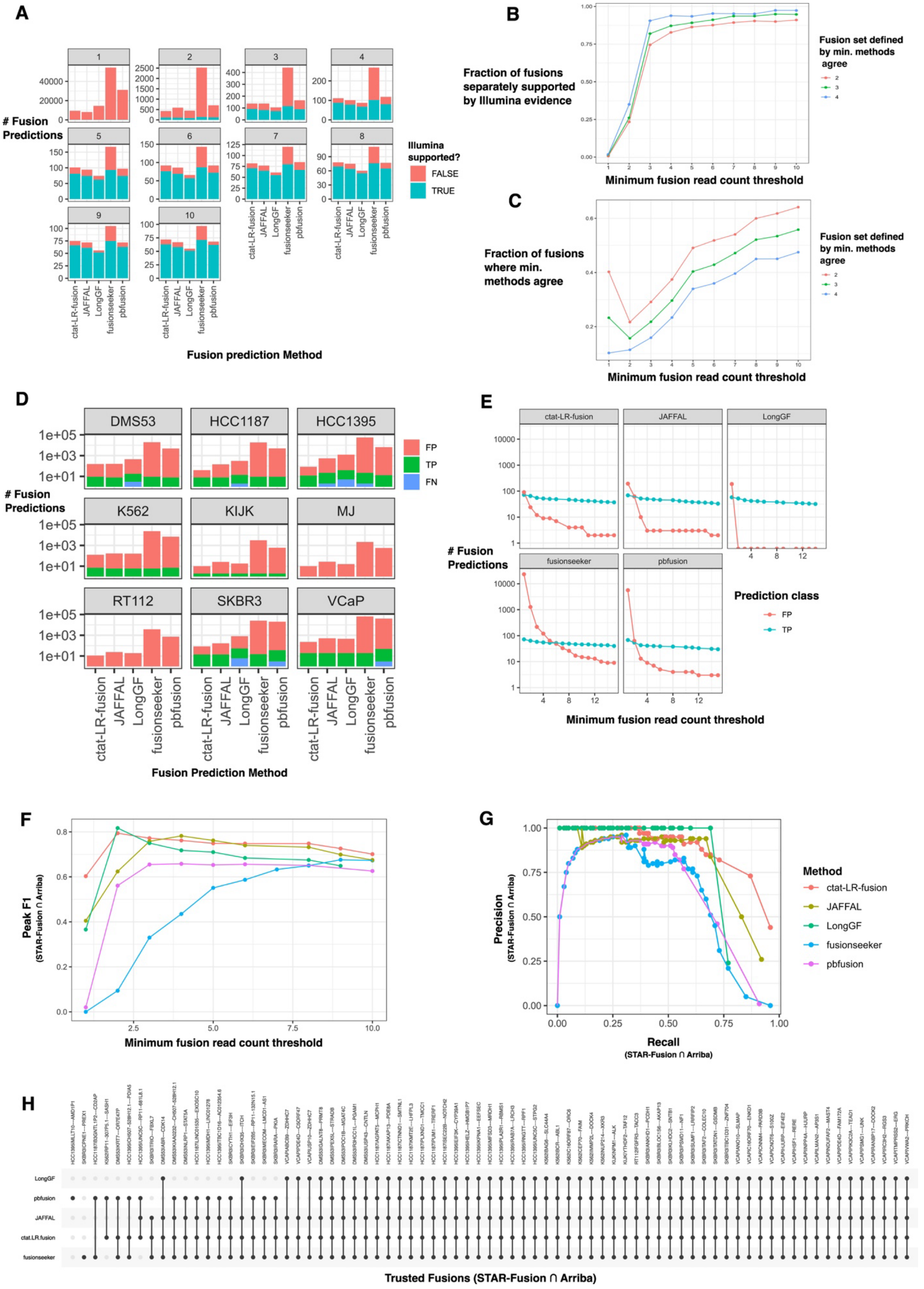
A
# Fusion Predictions
Illumina supported?
FALSE
TRUE
Fusion prediction Method
ctat-LR-fusion
JAFFAL
LongGF
fusionseeker
pbfusion
B
Fraction of fusions separately supported by Illumina evidence
Fusion set defined by min. methods agree
Minimum fusion read count threshold
C
Fraction of fusions where min. methods agree
Fusion set defined by min. methods agree
Minimum fusion read count threshold
D
DMS53
HCC1187
HCC1395
K562
KIJK
MJ
RT112
SKBR3
VCaP
FP
TP
FN
# Fusion Predictions
Fusion Prediction Method
E
# Fusion Predictions
Prediction class
FP
TP
Minimum fusion read count threshold
F
Peak F1
(STAR-Fusion ∩ Arriba)
Minimum fusion read count threshold
G
Precision
(STAR-Fusion ∩ Arriba)
Recall
(STAR-Fusion ∩ Arriba)
Method
ctat-LR-fusion
JAFFAL
LongGF
fusionseeker
pbfusion
H
LongGF
pbfusion
JAFFAL
ctat.LR.fusion
fusionseeker
Trusted Fusions (STAR-Fusion ∩ Arriba)

**Supplemental Figure S3: Evaluation of fusions detected in nine DepMap cell lines based on Illumina short read supported fusions as truth sets based on the intersection of Arriba and STAR-Fusion predictions (referred to below as *the example Illumina-based truth set*).** (A) Numbers of Illumina-supported and other fusion predictions by method according to corresponding minimum number of fusion evidence reads (1 to 10 minimum reads as indicated). (B) Fraction of agreed-upon long-read fusion predictions that are separately supported by Illumina short read based fusion predictions (both STAR-Fusion and Arriba). (C) Fraction of total fusions that agree according to minimum number of methods after filtering according to minimum read evidence thresholds. (D) Numbers of true positives (TP), false positives (FP) and false negatives (FN), (E) numbers of TP and FP vs. minimum fusion read evidence threshold, (F) peak F1 score vs. minimum read support threshold, and (G) precision-recall plot, and (H) UpSet plot for prediction of truth set fusions, all based on benchmarking with the *example Illumina-based truth set*. Similar reporting for all Illumina-based truth sets is available in **Supplementary Code**.

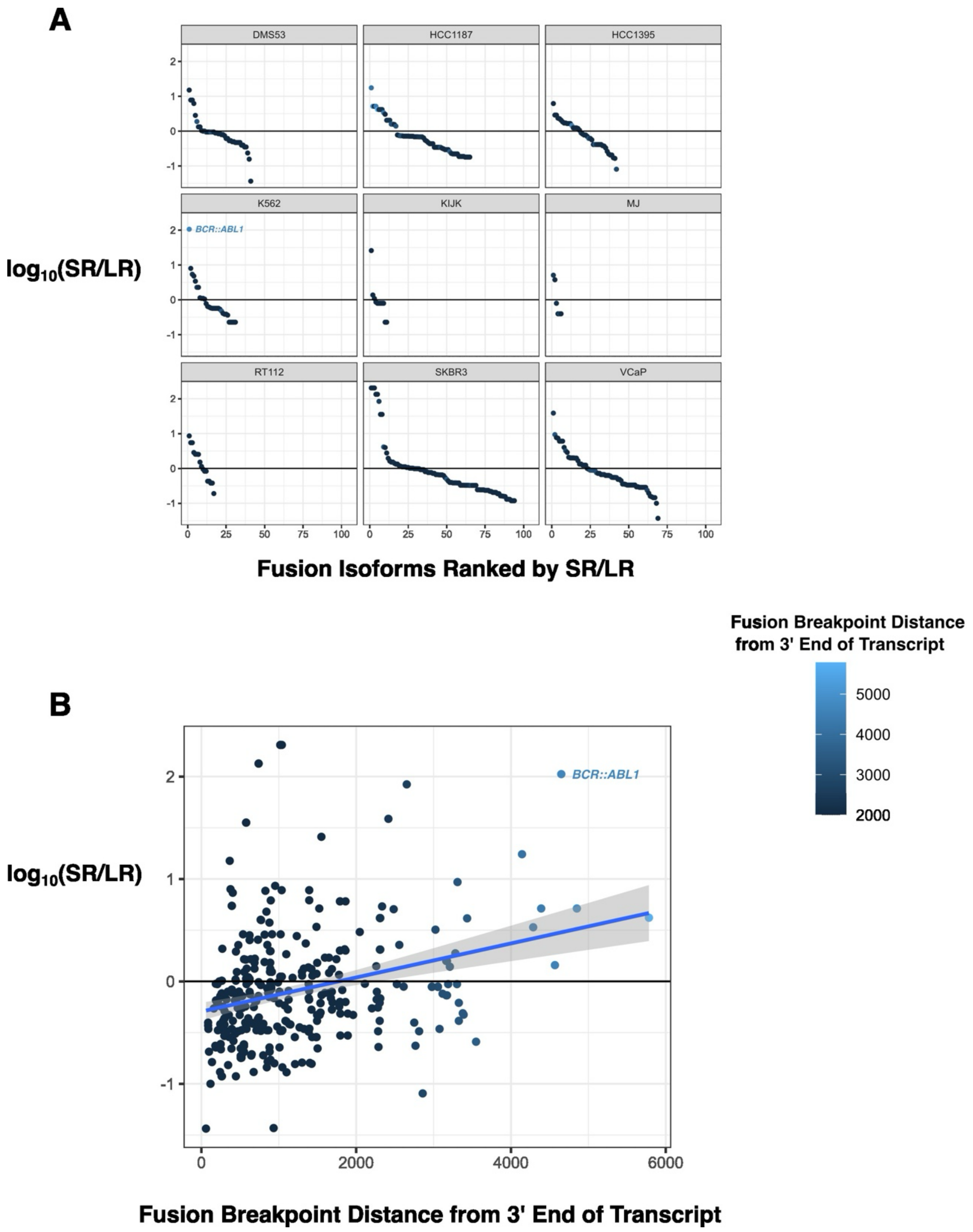


**Supplemental Figure S4: Short read (SR) vs. long read (LR) support for fusion evidence.** (A) Relative support of SR vs. LR support for fusion transcript isoforms, ranked by SR/LR and computed as $\log_{10}(SR/LR)$ where SR and LR are normalized according to sequenced number of bases. (B) SR vs. LR support for fusion isoforms according to median distance of the breakpoint to the end of the read alignment. Extreme outlier K562|BCR::ABL1 is indicated.

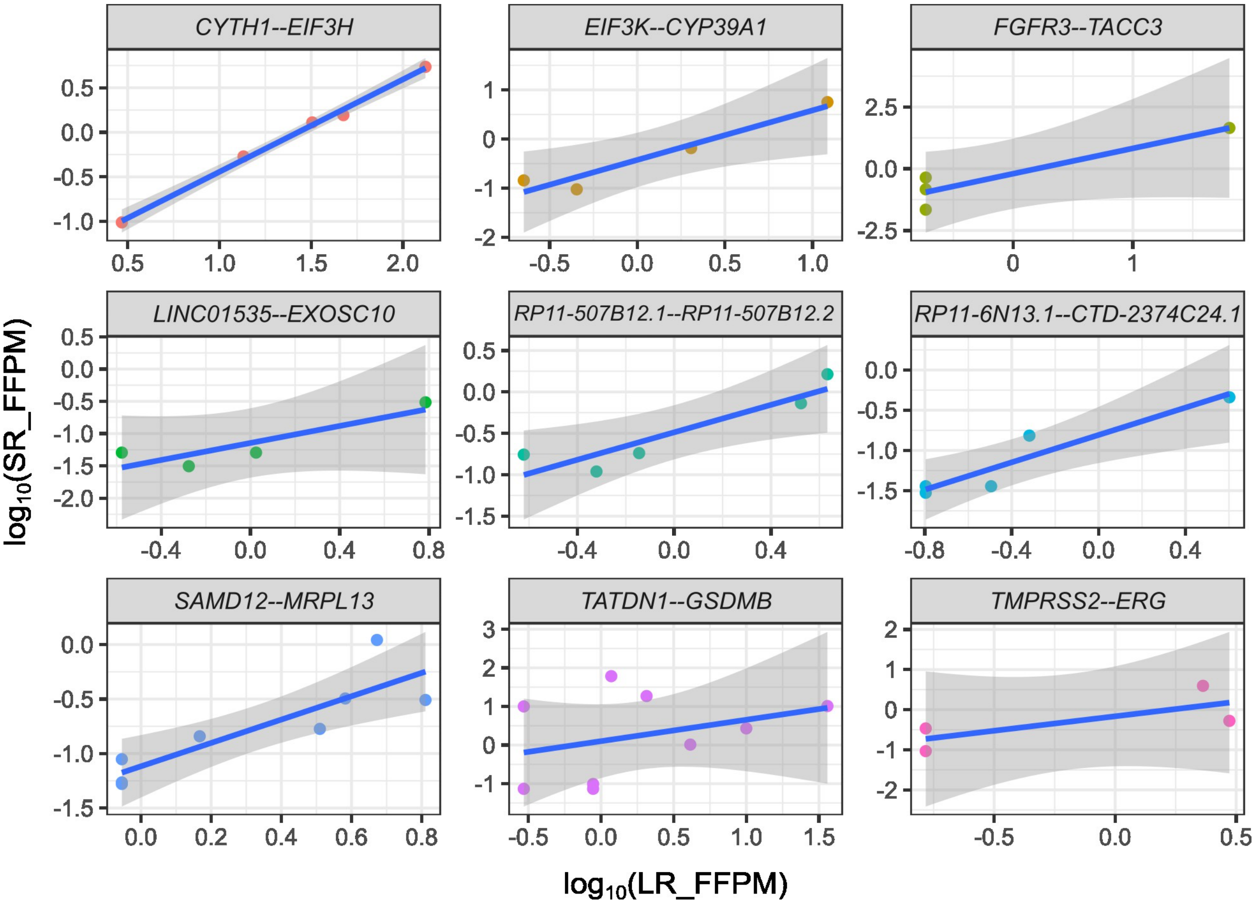


**Supplemental Figure S5: Comparison of short vs. long read support for fusion transcript isoforms.** Fusion expression evidence normalized as FFPM are compared for long reads (x-axis) and short reads (y-axis) for fusions *CYTH1::EIF3H* in SKBR3, *LINC01535::EXOSC10* in HCC1187, *CYP39A1--EIF3K* in HCC1395, *FGFR3--TACC3* in RT112, *RP11-507B12.1--RP11-507B12.2* in DMS53, *RP11-6N13.1::CTD-2374C24.1* in K562, *SAMD12::MRPL13* in SKBR3, *TATDN1::GSDMB* in SKBR3, and *TMPRSS2::ERG* in VCaP.

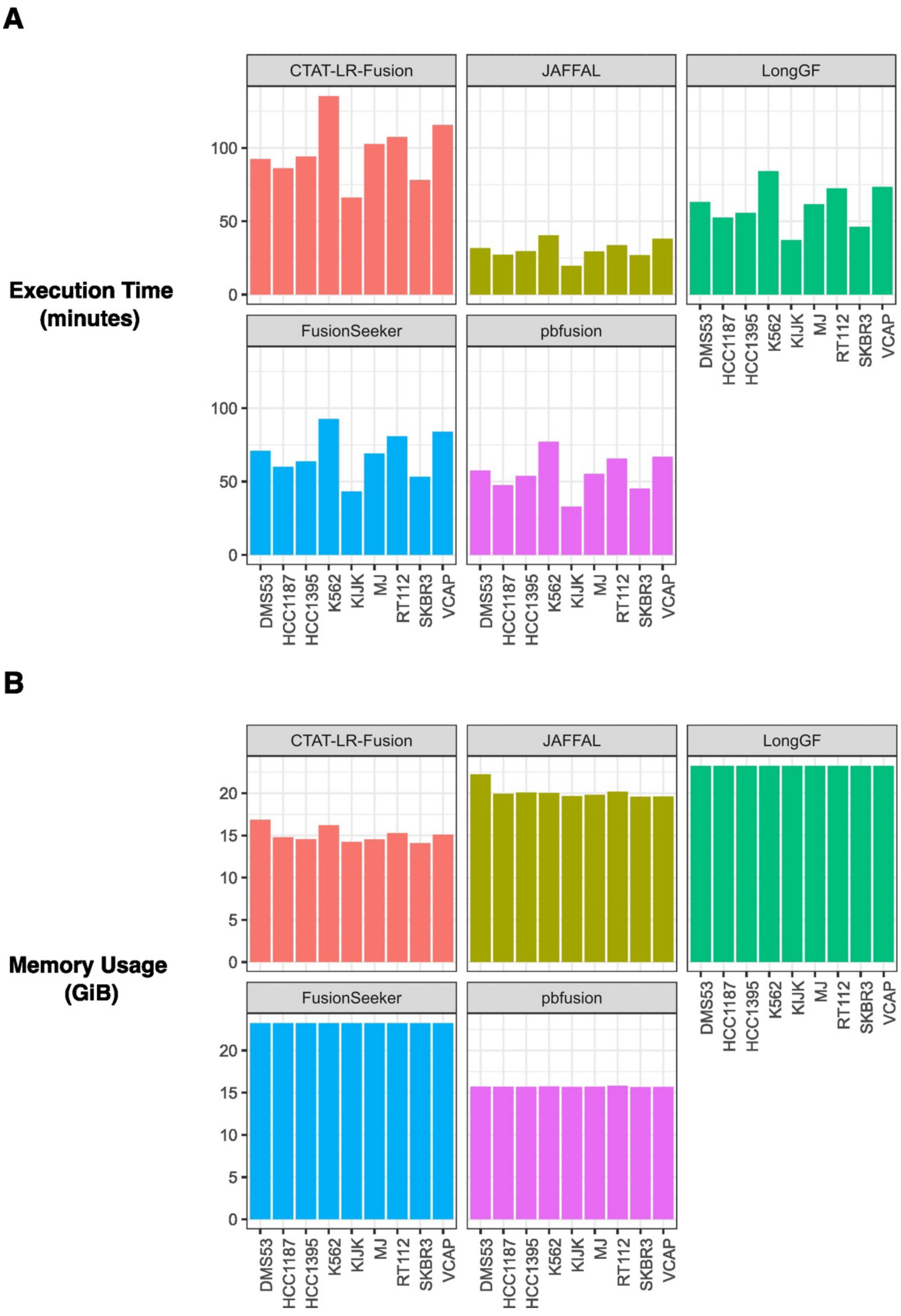


**Supplemental Figure S6: Comparison of running time and memory usage for different methods in processing each of the nine DepMap cancer cell lines**. (A) Execution time in minutes. (B) Maximum memory usage in gigabytes. Samples were processed on http://Terra.bio using the **Supplementary Code** “0.Workflows_and_Dockers/wdlworkflow long_comb_fusion.wdl”.
