# Supplementary figures and images for: Accurate fusion transcript identification from long- and short-read isoform sequencing at bulk or single-cell resolution
Source: Genome Res. 2025 Apr;35(4):967–86. doi: 10.1101/gr.279200.124 (PMC12047241; doi:10.1101/gr.279200.124)

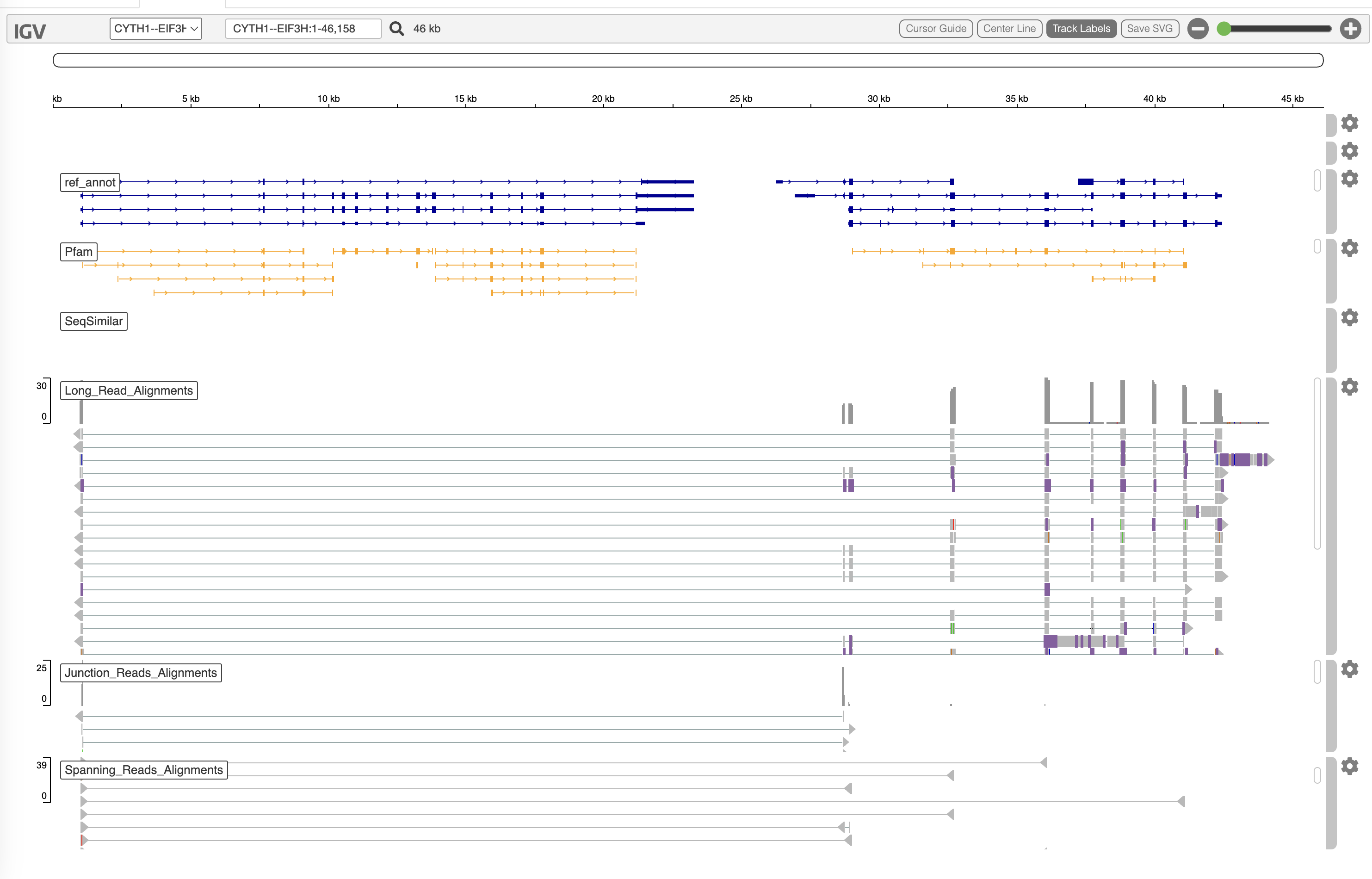

Supplement: Supplement 11 [file Supplemental_File_1.zip › CTAT-LR-fusion.v1.0.0/CTAT-LR-fusion.wiki/images/LR_FI_example.png]

# VCAP TMRSS2--ERG by FFPM

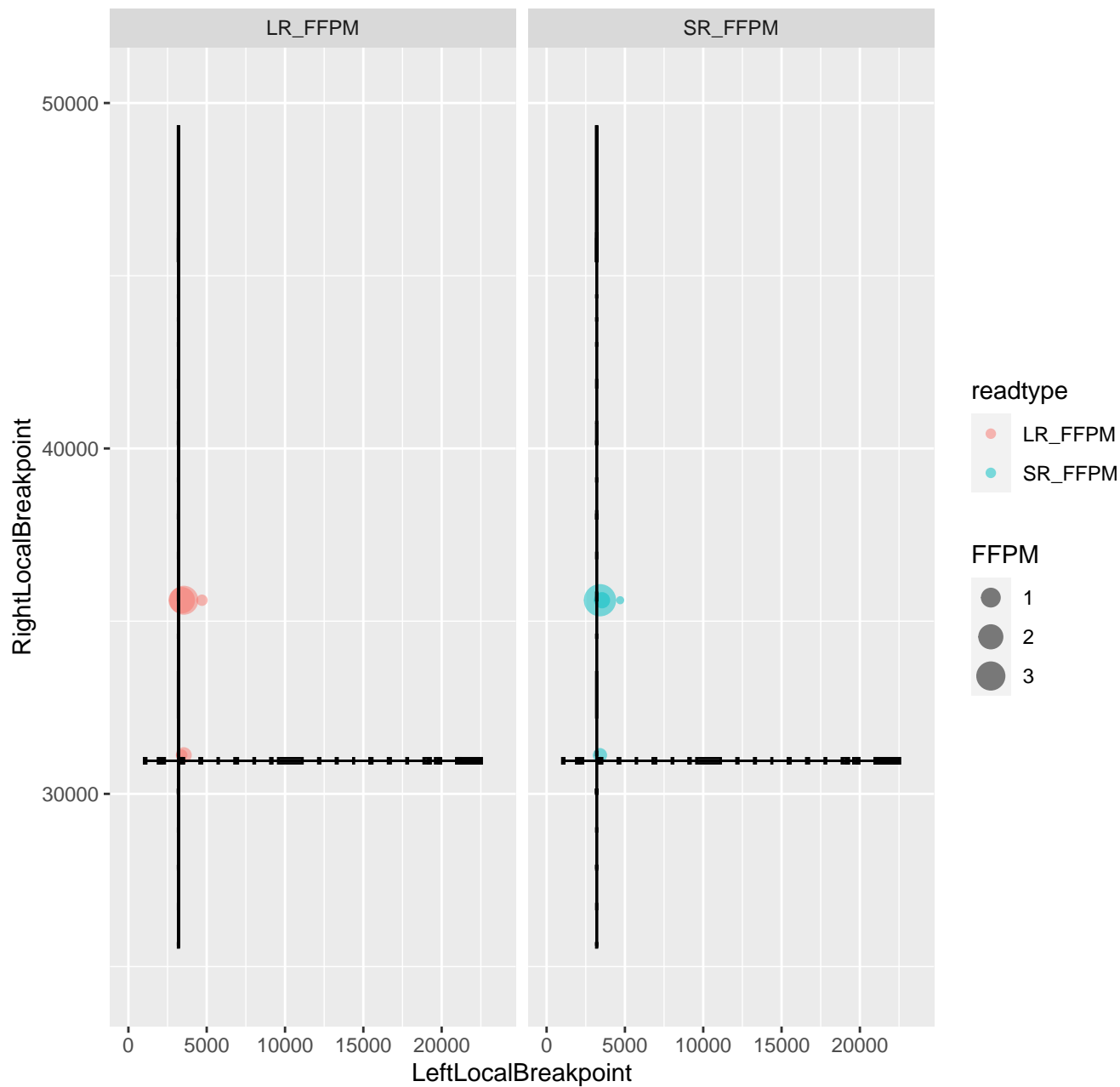

Supplement: Supplement 12 [file Supplemental_File_2.zip › CTAT-LRF-Paper/3.DepMap9Lines/3a.CTAT_DepMap9Lines/depmap_VCaP_TMPRSS2--ERG_LR_vs_SR_isoforms.pdf]

# M132TS, Fusion: NUTM2A-AS1--RP11-203L2.4

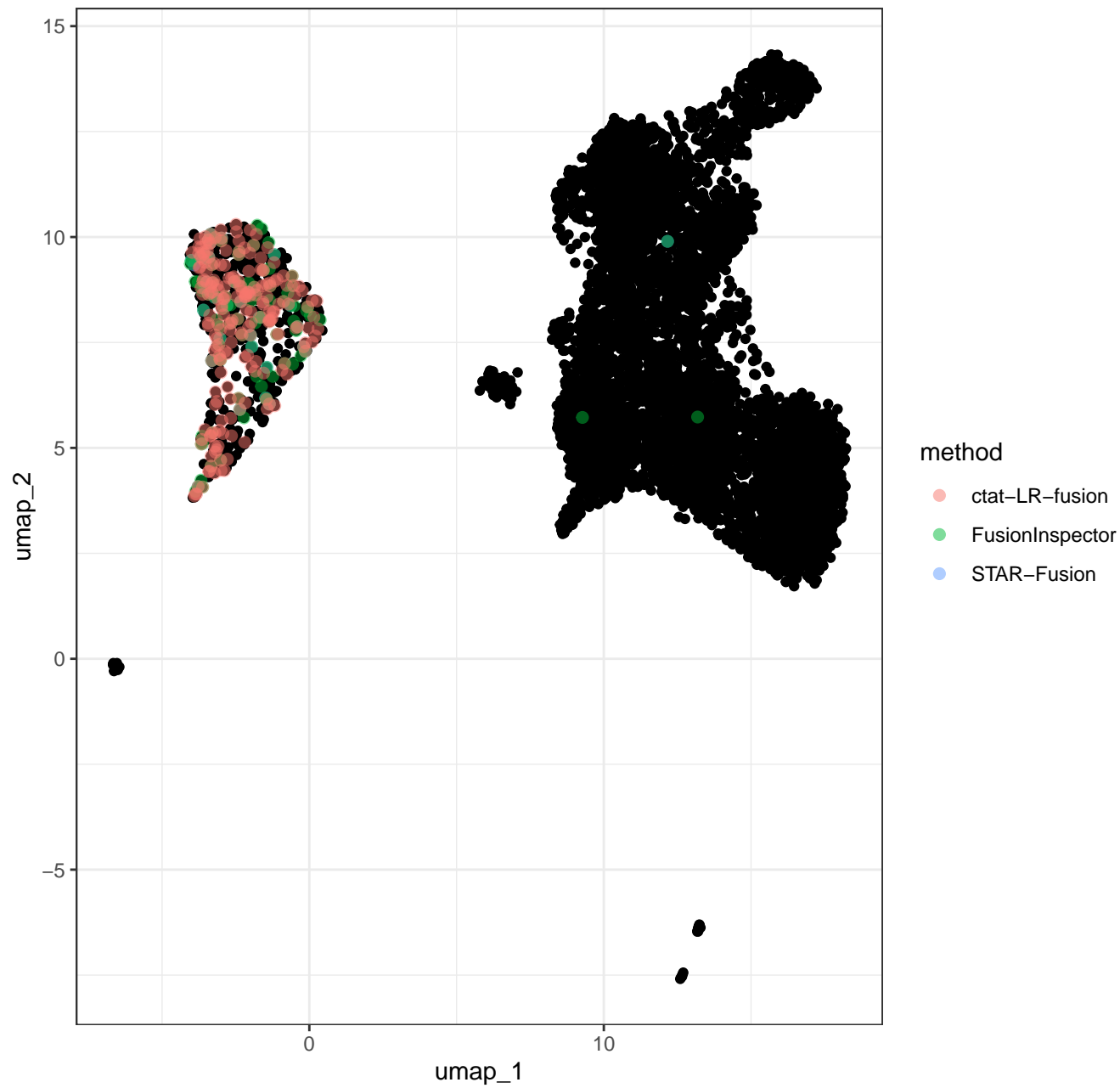

Supplement: Supplement 12 [file Supplemental_File_2.zip › CTAT-LRF-Paper/4.SingleCellFusions/4a.sc_Melanoma/M132TS.fusions_of_interest.pdf]

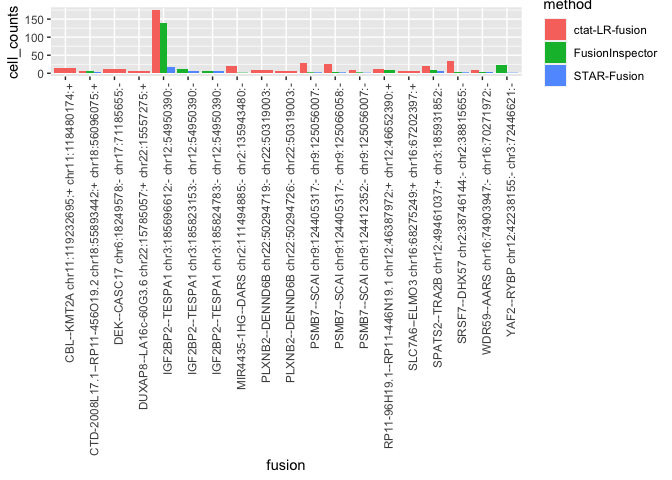

Supplement: Supplement 12 [file Supplemental_File_2.zip › CTAT-LRF-Paper/4.SingleCellFusions/4b.sc_HGSOC/Patient2_analysis_files/figure-gfm/unnamed-chunk-8-1.png]

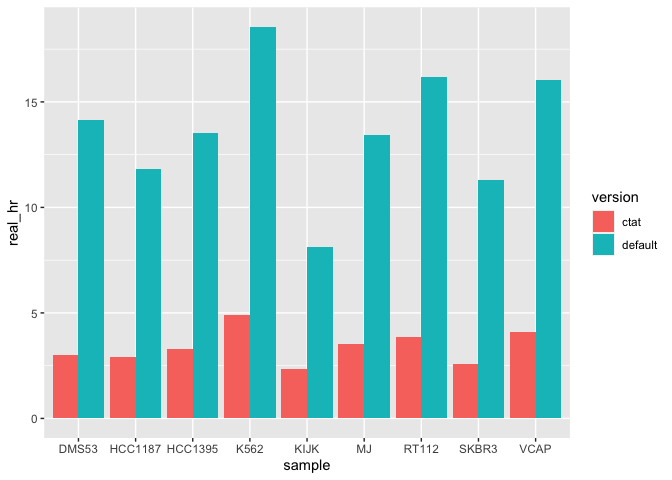

Supplement: Supplement 12 [file Supplemental_File_2.zip › CTAT-LRF-Paper/5.Misc/5.1.ctat-mm2-timings/ctat-mm2-timings_files/figure-gfm/unnamed-chunk-3-1.png]

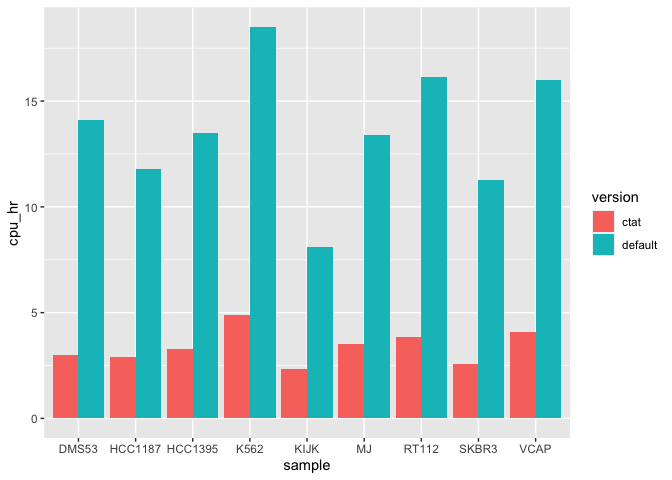

Supplement: Supplement 12 [file Supplemental_File_2.zip › CTAT-LRF-Paper/5.Misc/5.1.ctat-mm2-timings/ctat-mm2-timings_files/figure-gfm/unnamed-chunk-4-1.png]

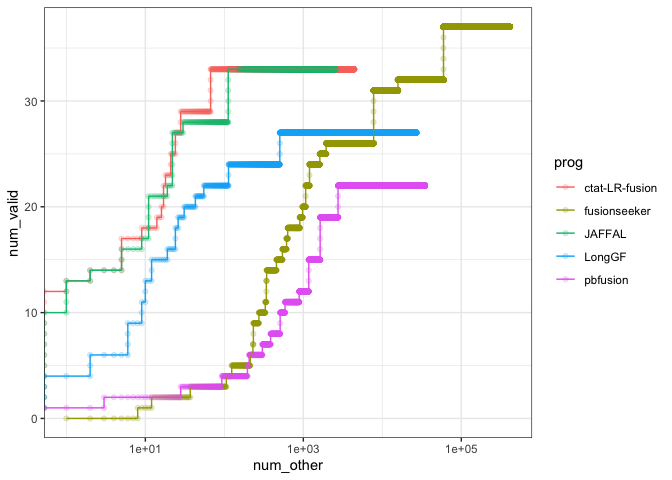

Supplement: Supplement 12 [file Supplemental_File_2.zip › CTAT-LRF-Paper/6.SGNEx_ONT_cell_lines/SGNex_ONT_eval_files/figure-gfm/unnamed-chunk-14-1.png]

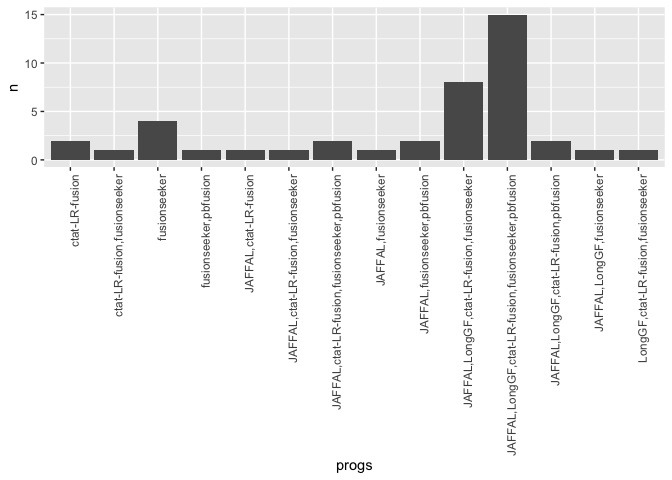

Supplement: Supplement 12 [file Supplemental_File_2.zip › CTAT-LRF-Paper/6.SGNEx_ONT_cell_lines/SGNex_ONT_eval_files/figure-gfm/unnamed-chunk-19-1.png]

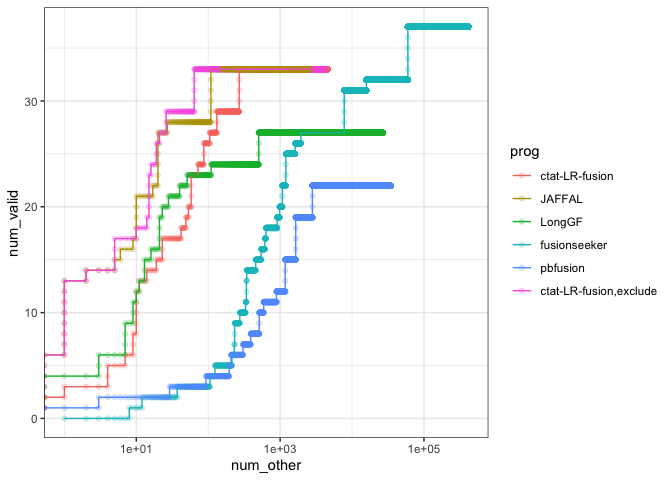

Supplement: Supplement 12 [file Supplemental_File_2.zip › CTAT-LRF-Paper/6.SGNEx_ONT_cell_lines/SGNex_ONT_eval_files/figure-gfm/unnamed-chunk-20-1.png]

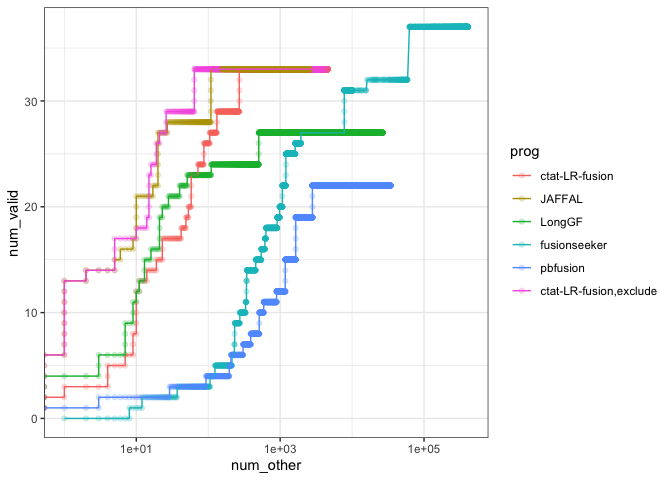

Supplement: Supplement 12 [file Supplemental_File_2.zip › CTAT-LRF-Paper/6.SGNEx_ONT_cell_lines/SGNex_ONT_eval_files/figure-gfm/unnamed-chunk-23-1.png]

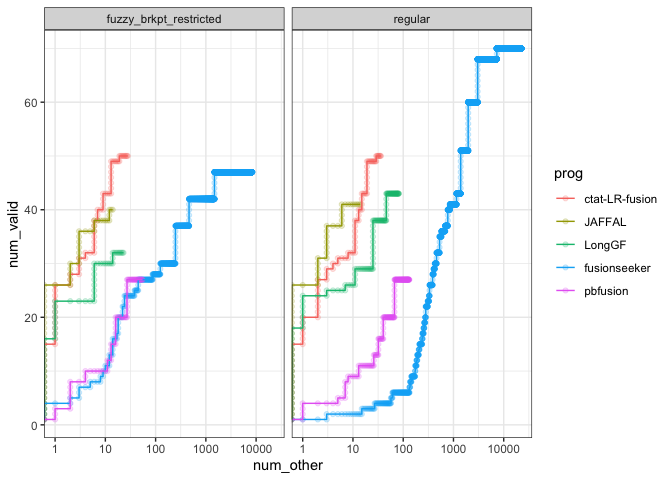

Supplement: Supplement 12 [file Supplemental_File_2.zip › CTAT-LRF-Paper/6.SGNEx_ONT_cell_lines/SGNex_ONT_eval_files/figure-gfm/unnamed-chunk-25-1.png]

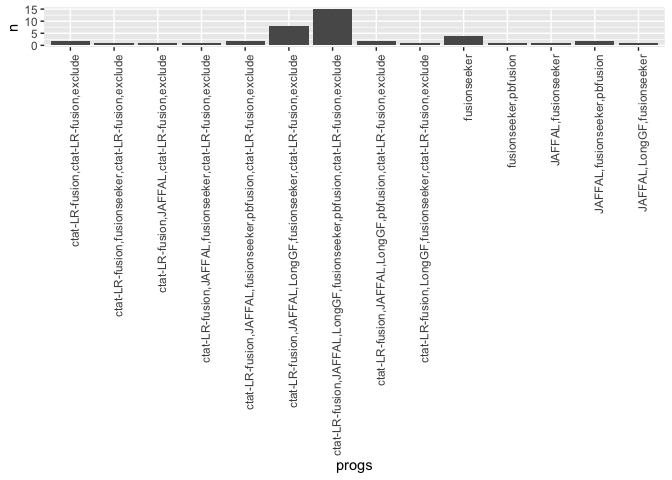

Supplement: Supplement 12 [file Supplemental_File_2.zip › CTAT-LRF-Paper/6.SGNEx_ONT_cell_lines/SGNex_ONT_eval_files/figure-gfm/unnamed-chunk-26-1.png]

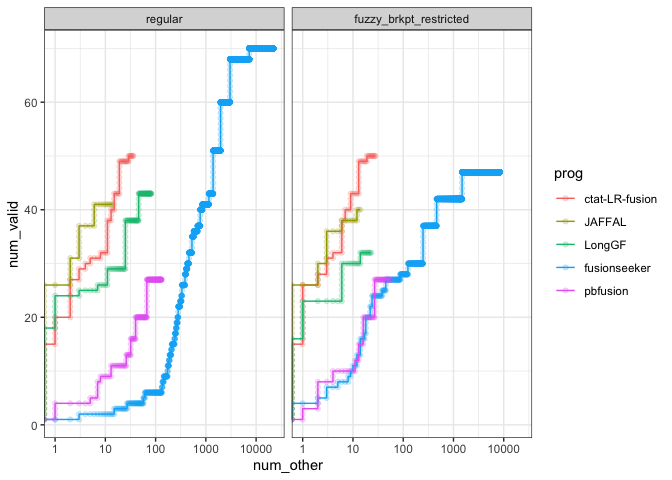

Supplement: Supplement 12 [file Supplemental_File_2.zip › CTAT-LRF-Paper/6.SGNEx_ONT_cell_lines/SGNex_ONT_eval_files/figure-gfm/unnamed-chunk-28-1.png]

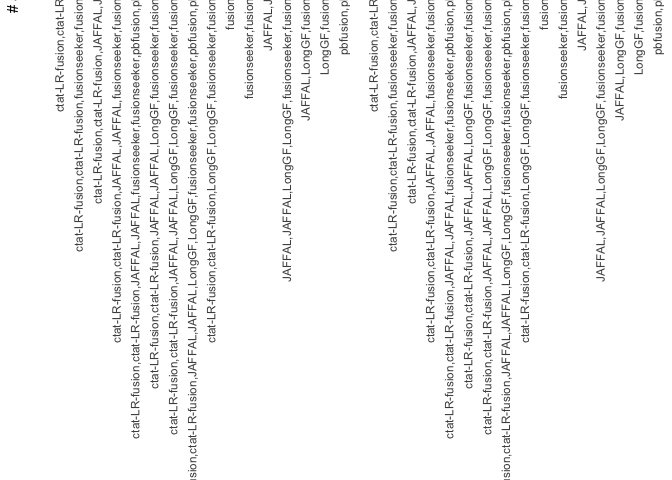

Supplement: Supplement 12 [file Supplemental_File_2.zip › CTAT-LRF-Paper/6.SGNEx_ONT_cell_lines/SGNex_ONT_eval_files/figure-gfm/unnamed-chunk-32-1.png]
