# Supplementary material for: Accurate fusion transcript identification from long- and short-read isoform sequencing at bulk or single-cell resolution
Source: Genome Res. 2025 Apr;35(4):967–86. doi: 10.1101/gr.279200.124 (PMC12047241; doi:10.1101/gr.279200.124)
Supplement: Supplement 12 [file Supplemental_File_2.zip › CTAT-LRF-Paper/2.SeraCareFusions/__SeraCareDocs/coa-0710-0497-seraseq-fusion-rna-mix-v4-10646847.pdf]

Certificate of Analysis
For Research Use Only, Not for use in Diagnostic Procedures

Product Description:	Seraseq Fusion RNA Mix v4		
Material No:	0710-0497	Batch No:	10646847
Date of Manufacture:	05 JAN 2023	Expiration Date:	07 JUN 2024

Nominal Concentration:	25 ng / µL	Volume:	25 µL
Concentration test Method:	Thermo Fisher Qubit RNA BR Assay		
Measured Concentration:	26 ng / µL		

Nominal Fusion Concentration: 1500 Fusion copies/µL

Fusion Test Method: Droplet Digital PCR using TaqMan™ probes run on the BioRad QX200 system

Measured Fusion Concentrations:

RNA Fusion	Digital PCR Average Fusion copies/µL
CCDC6-RET	1432
CD74-ROS1	1639
EGFR variant III	3976
EGFR-SEPT14	1551
EML4-ALK	1624
ETV6-NTRK3	1364
FGFR3-BAIAP2L1	1293
FGFR3-TACC3	3373
KIF5B-RET	2371
LMNA-NTRK1	1608
MET Exon 14 Skipping	1587
NCOA4-RET	2007
PAX8-PPARG1	1761
SLC34A2-ROS1	2249
SLC45A3-BRAF	1577
TFG-NTRK1	1216
TMPRSS2-ERG	3173
TPM3-NTRK1	1364

Certificate of Analysis
For Research Use Only, Not for use in Diagnostic Procedures

NGS Result: Positive for each of the 18 fusions and exon skipping events

NGS Fusion Test Method: Archer® FusionPlex® Solid Tumor Assay run on the Illumina® MiSeq™ instrument (300-cycle Reagent Kit v2,) at 250 ng RNA input

NGS Analysis Method: Data analyzed using Archer Analysis Suite Software version 6.2.7 (default parameters).

NGS Data:

RNA Fusion	NGS Average Unique Start Sites per Fusion	NGS Average Unique Reads per Fusion*
CCDC6-RET	147	836
CD74-ROS1	136	1009
EGFR variant III	134	425
EGFR-SEPT14	311	940
EML4-ALK	176	1628
ETV6-NTRK3	372	1747
FGFR3-BAIAP2L1	151	1442
FGFR3-TACC3	194	2266
KIF5B-RET	224	2910
LMNA-NTRK1	245	3630
MET Exon 14 Skipping	161	494
NCOA4-RET	133	922
PAX8-PPARG1	118	704
SLC34A2-ROS1	162	1360
SLC45A3-BRAF	92	6438
TFG-NTRK1	189	1703
TMPRSS2-ERG	145	11100
TPM3-NTRK1	341	3474

*Total number of reads per sample was 5.2 M.

Approval:

Prepared By — Date: 16 JAN 2023

QA Verified By — Date: 17 JAN 2023
