# Supplementary material for: Accurate fusion transcript identification from long- and short-read isoform sequencing at bulk or single-cell resolution
Source: Genome Res. 2025 Apr;35(4):967–86. doi: 10.1101/gr.279200.124 (PMC12047241; doi:10.1101/gr.279200.124)
Supplement: Supplement 12 [file Supplemental_File_2.zip › CTAT-LRF-Paper/4.SingleCellFusions/4b.sc_HGSOC/Patient2_Tum.fusions_of_interest.pdf]

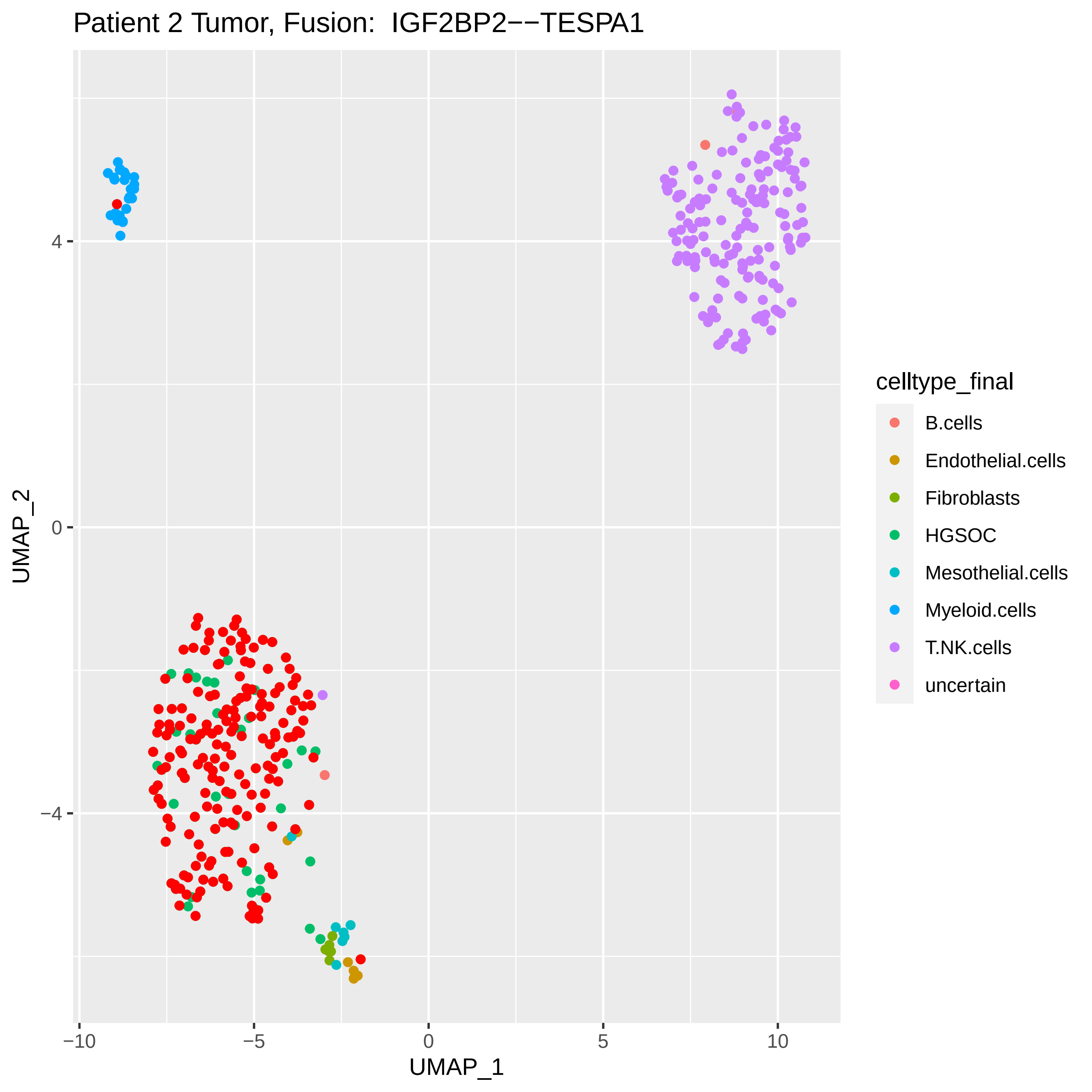
Patient 2 Tumor, Fusion: IGF2BP2−−TESPA1
UMAP_2
4
0
−4
−10
−5
0
5
10
UMAP_1
celltype_final
B.cells
Endothelial.cells
Fibroblasts
HGSOC
Mesothelial.cells
Myeloid.cells
T.NK.cells
uncertain

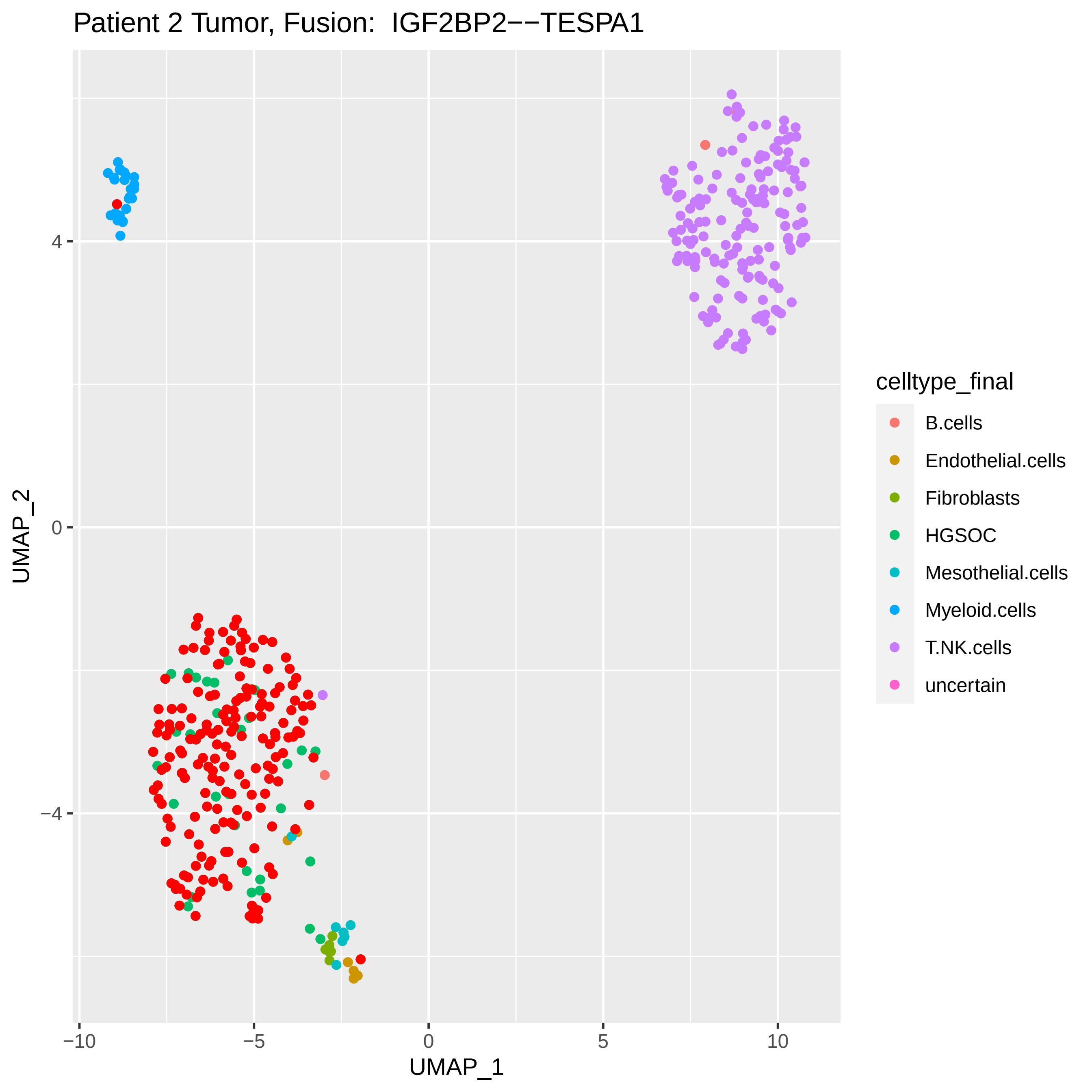

Patient 2 Tumor, Fusion: IGF2BP2−−TESPA1
UMAP_2
UMAP_1
4
0
−4
−10
−5
0
5
10
celltype_final
B.cells
Endothelial.cells
Fibroblasts
HGSOC
Mesothelial.cells
Myeloid.cells
T.NK.cells
uncertain

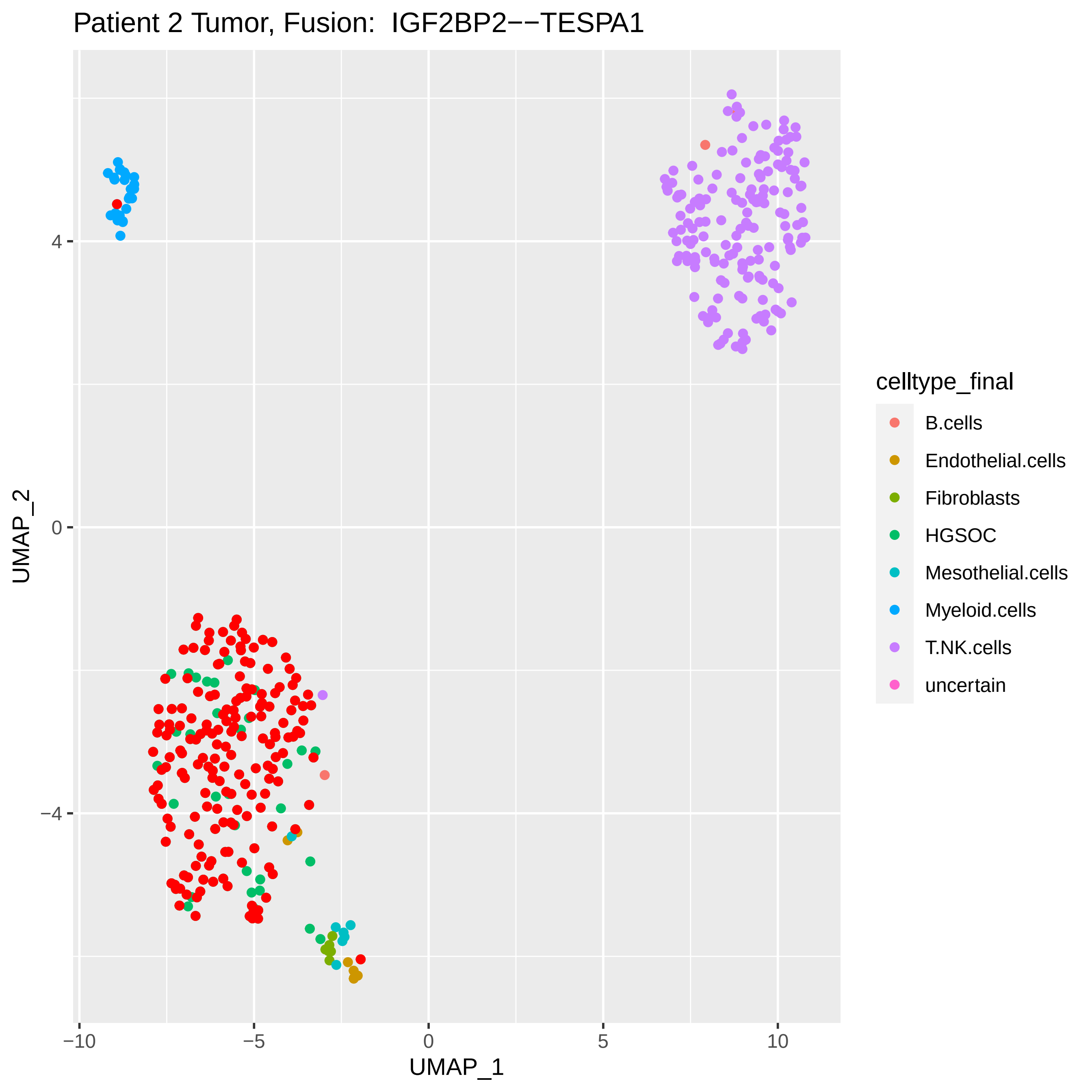
Patient 2 Tumor, Fusion: IGF2BP2−−TESPA1
UMAP_2
4
0
−4
−10
−5
0
5
10
UMAP_1
celltype_final
B.cells
Endothelial.cells
Fibroblasts
HGSOC
Mesothelial.cells
Myeloid.cells
T.NK.cells
uncertain

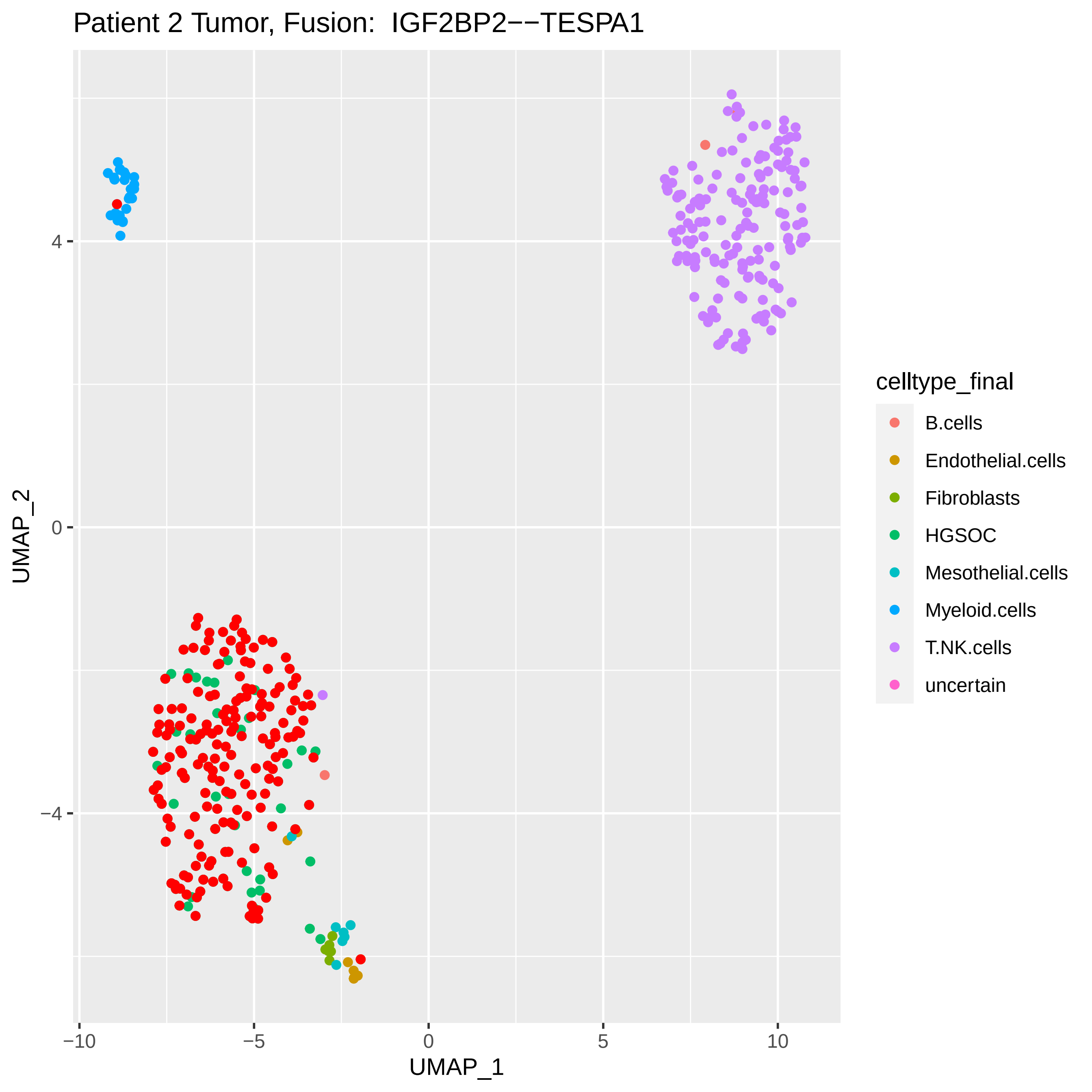

Patient 2 Tumor, Fusion: IGF2BP2−−TESPA1
UMAP_2
4
0
−4
−10
−5
0
5
10
UMAP_1
celltype_final
B.cells
Endothelial.cells
Fibroblasts
HGSOC
Mesothelial.cells
Myeloid.cells
T.NK.cells
uncertain

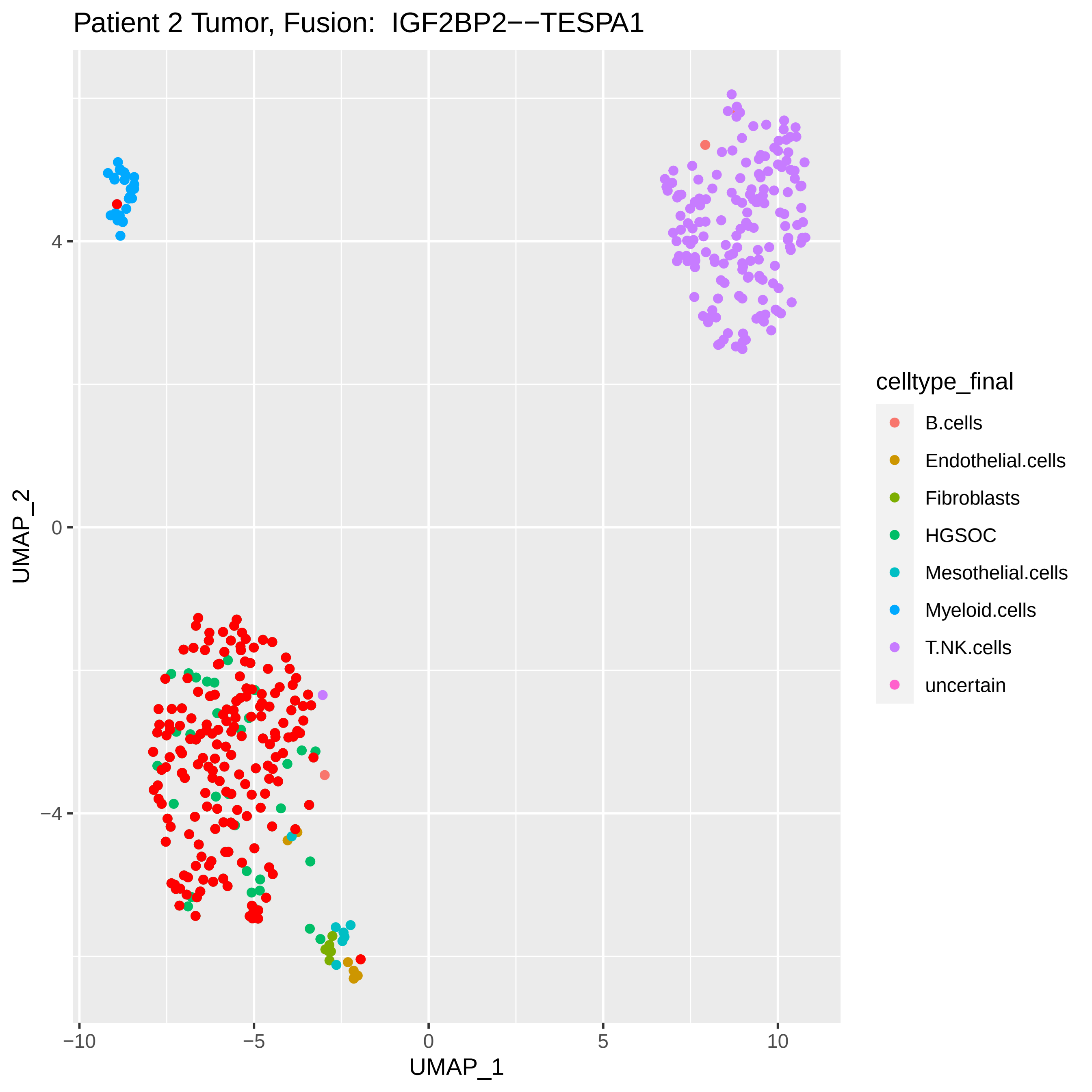
Patient 2 Tumor, Fusion: IGF2BP2−−TESPA1
UMAP_2
UMAP_1
4
0
−4
−10
−5
0
5
10
celltype_final
B.cells
Endothelial.cells
Fibroblasts
HGSOC
Mesothelial.cells
Myeloid.cells
T.NK.cells
uncertain

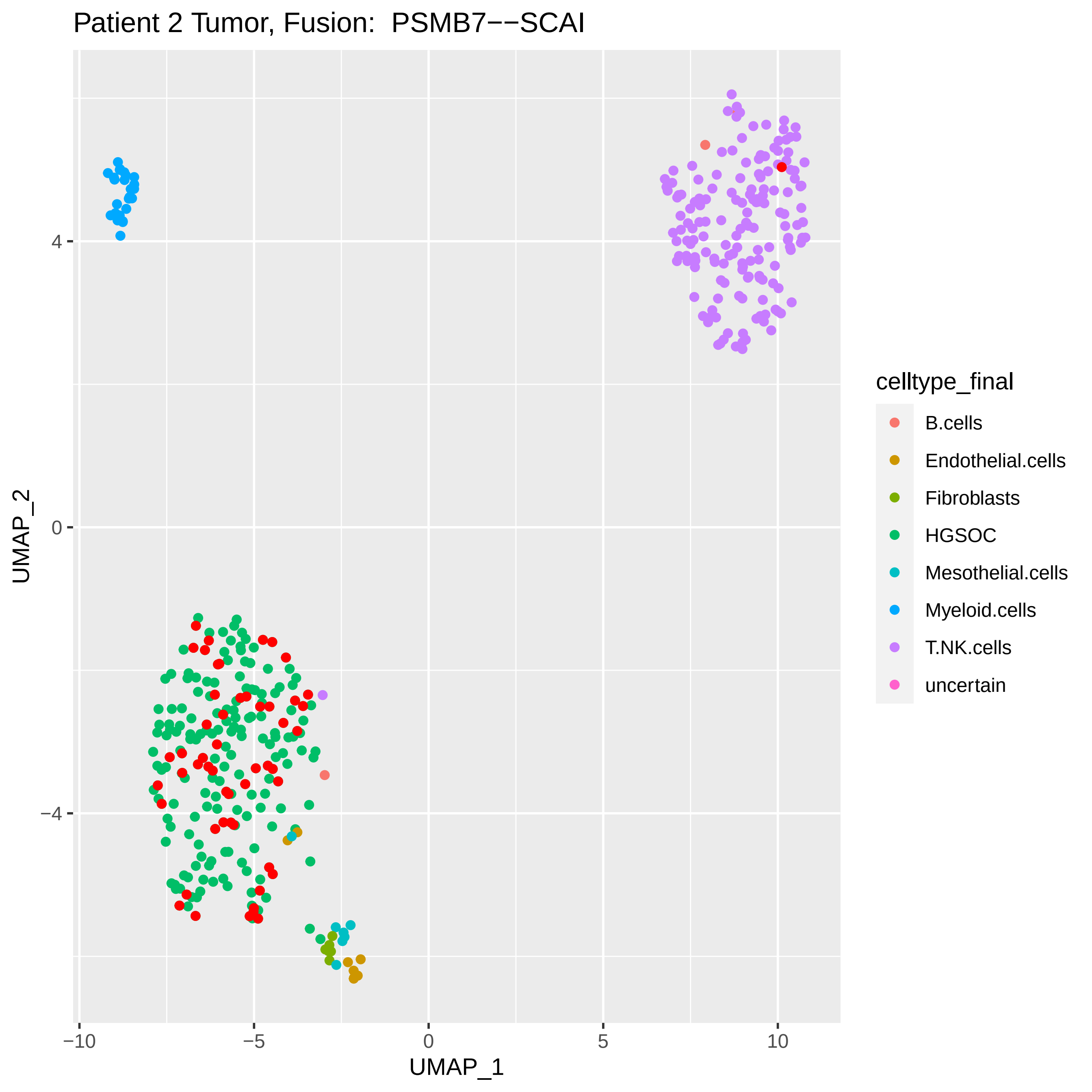

Patient 2 Tumor, Fusion: PSMB7--SCAI
UMAP_2
UMAP_1
4
0
−4
−10
−5
0
5
10
celltype_final
B.cells
Endothelial.cells
Fibroblasts
HGSOC
Mesothelial.cells
Myeloid.cells
T.NK.cells
uncertain

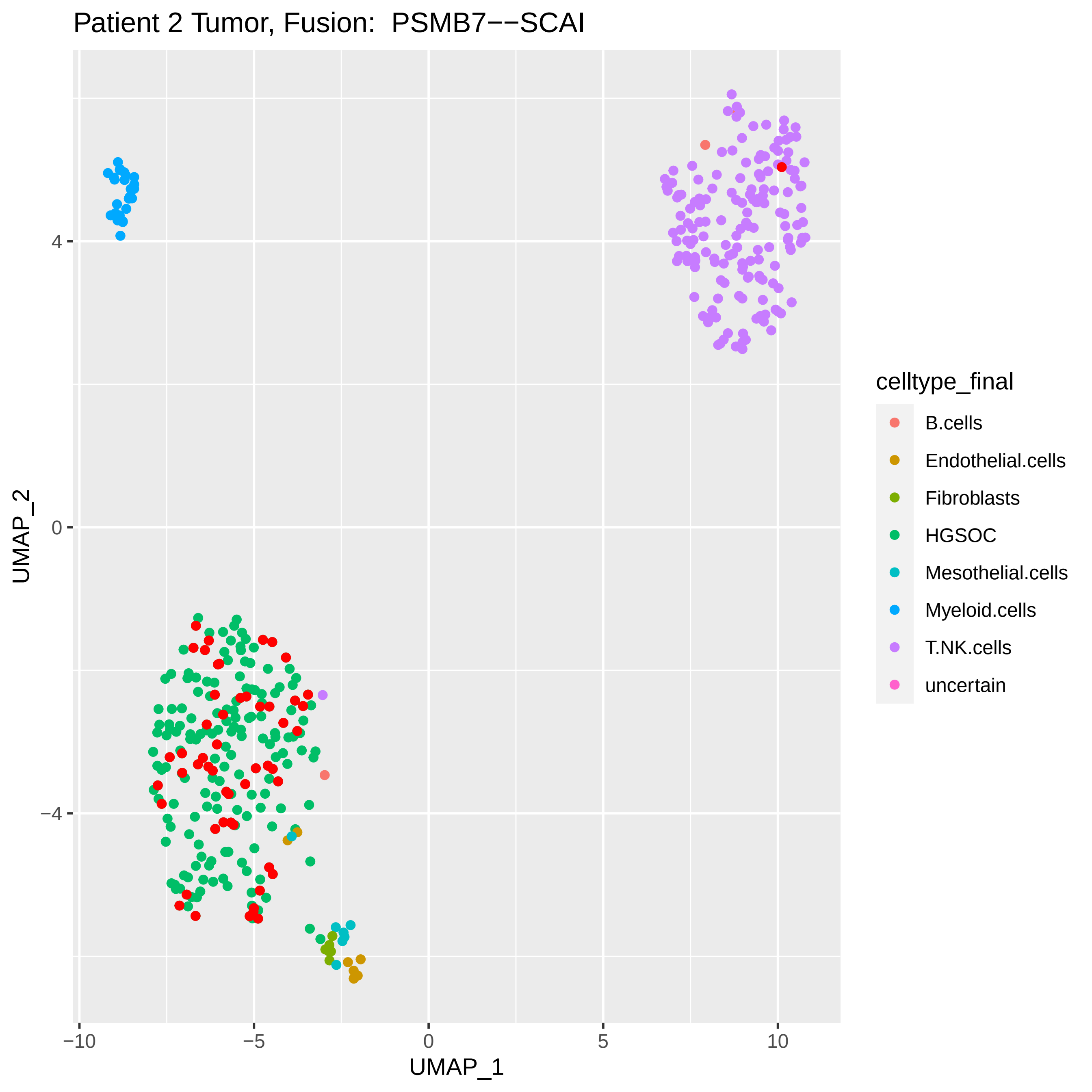
Patient 2 Tumor, Fusion: PSMB7−−SCAI
UMAP_2
UMAP_1
4
0
−4
−10
−5
0
5
10
celltype_final
B.cells
Endothelial.cells
Fibroblasts
HGSOC
Mesothelial.cells
Myeloid.cells
T.NK.cells
uncertain

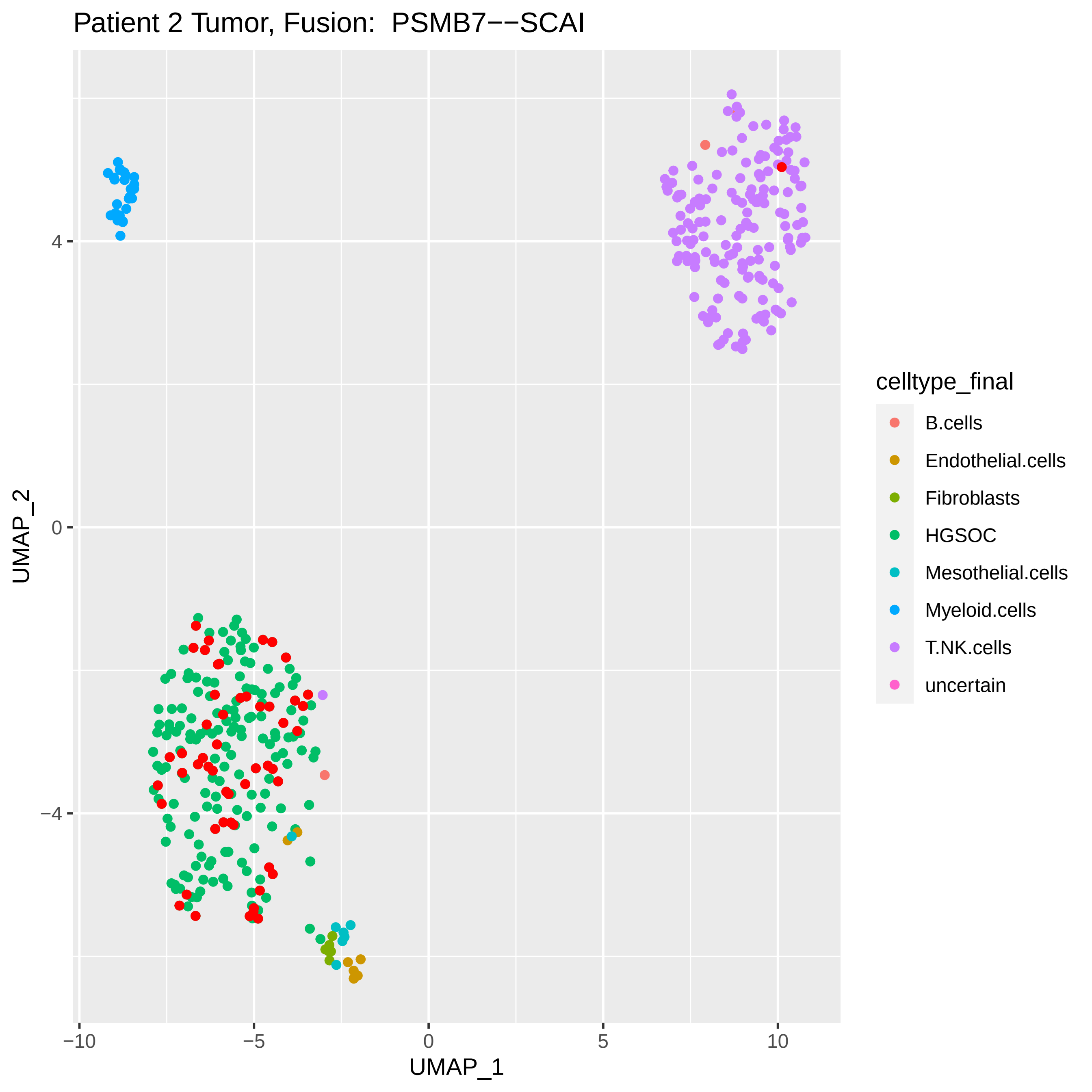
Patient 2 Tumor, Fusion: PSMB7−−SCAI
UMAP_2
4
0
−4
−10
−5
0
5
10
UMAP_1
celltype_final
B.cells
Endothelial.cells
Fibroblasts
HGSOC
Mesothelial.cells
Myeloid.cells
T.NK.cells
uncertain

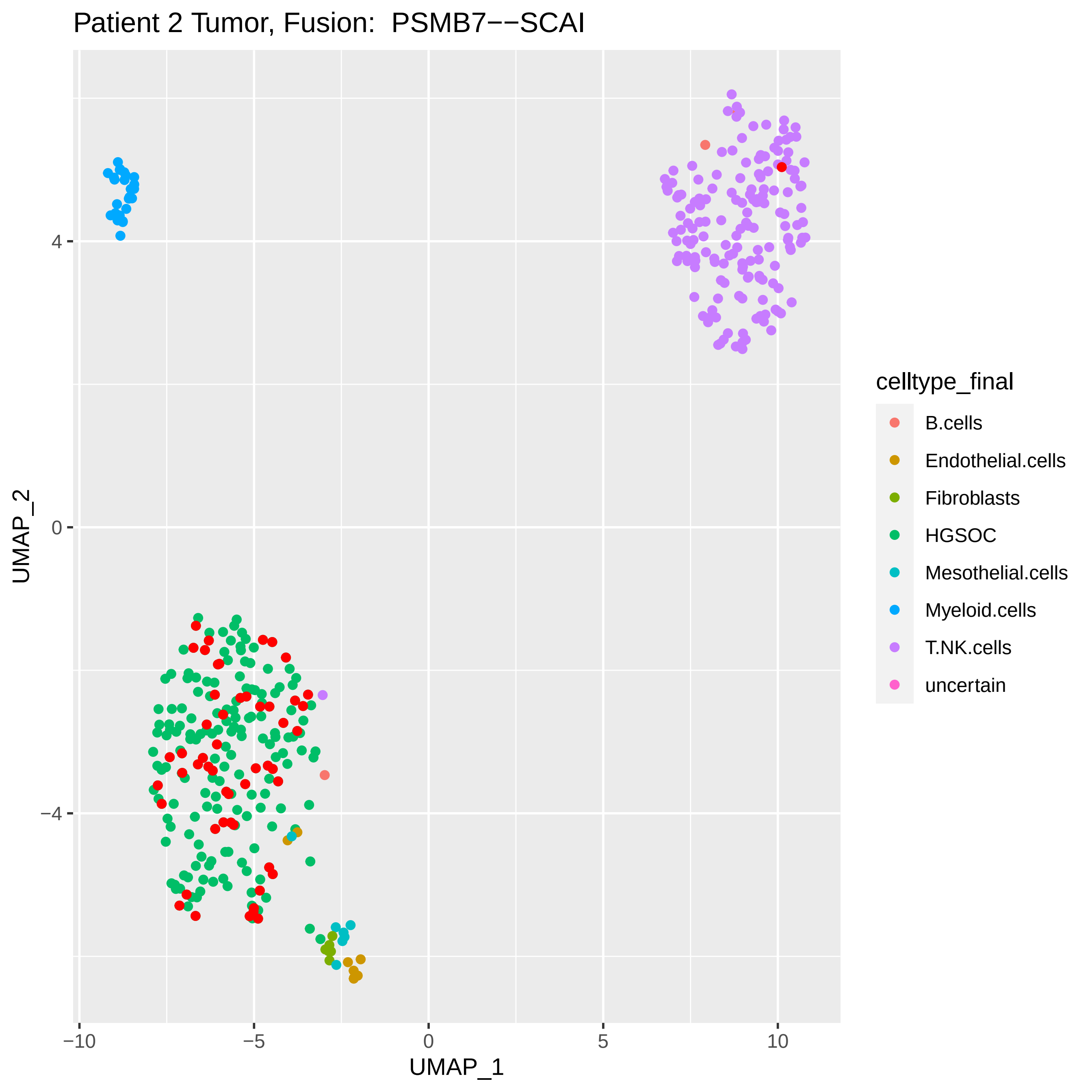

Patient 2 Tumor, Fusion: PSMB7−−SCAI
UMAP_2
UMAP_1
4
0
−4
−10
−5
0
5
10
celltype_final
B.cells
Endothelial.cells
Fibroblasts
HGSOC
Mesothelial.cells
Myeloid.cells
T.NK.cells
uncertain

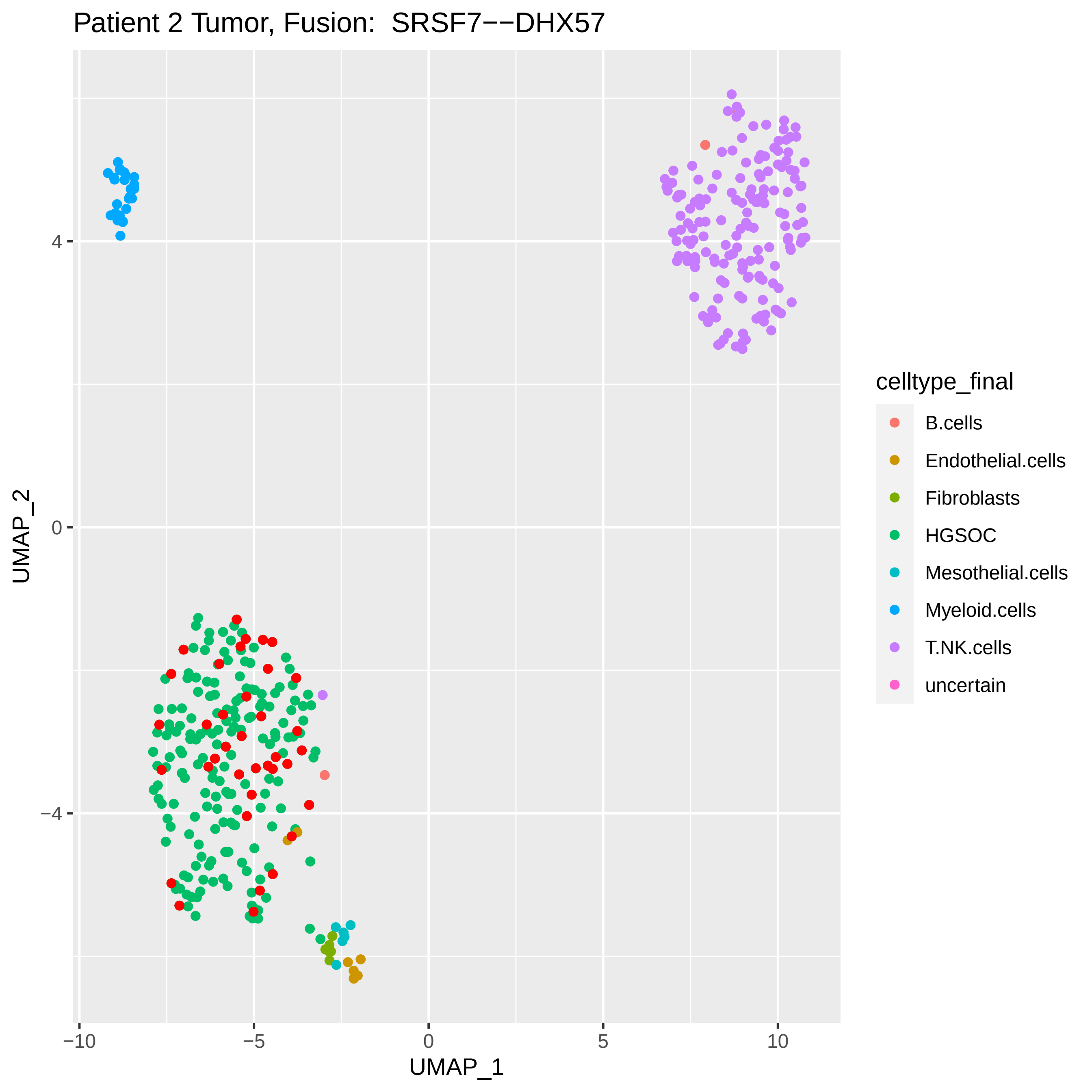
Patient 2 Tumor, Fusion: SRSF7--DHX57
UMAP_2
UMAP_1
4
0
−4
−10
−5
0
5
10
celltype_final
B.cells
Endothelial.cells
Fibroblasts
HGSOC
Mesothelial.cells
Myeloid.cells
T.NK.cells
uncertain

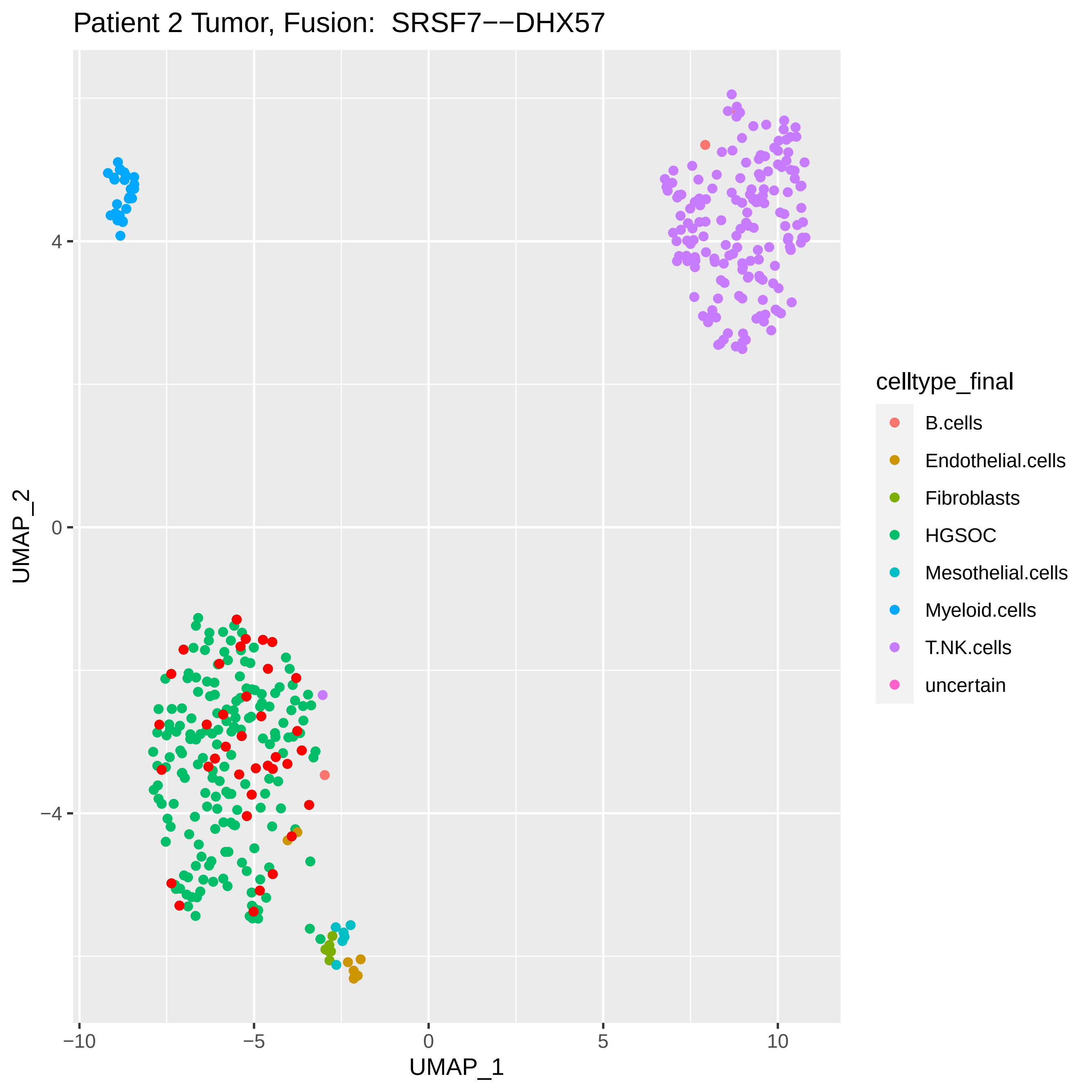

Patient 2 Tumor, Fusion: SRSF7--DHX57
UMAP_2
UMAP_1
4
0
−4
−10
−5
0
5
10
celltype_final
B.cells
Endothelial.cells
Fibroblasts
HGSOC
Mesothelial.cells
Myeloid.cells
T.NK.cells
uncertain

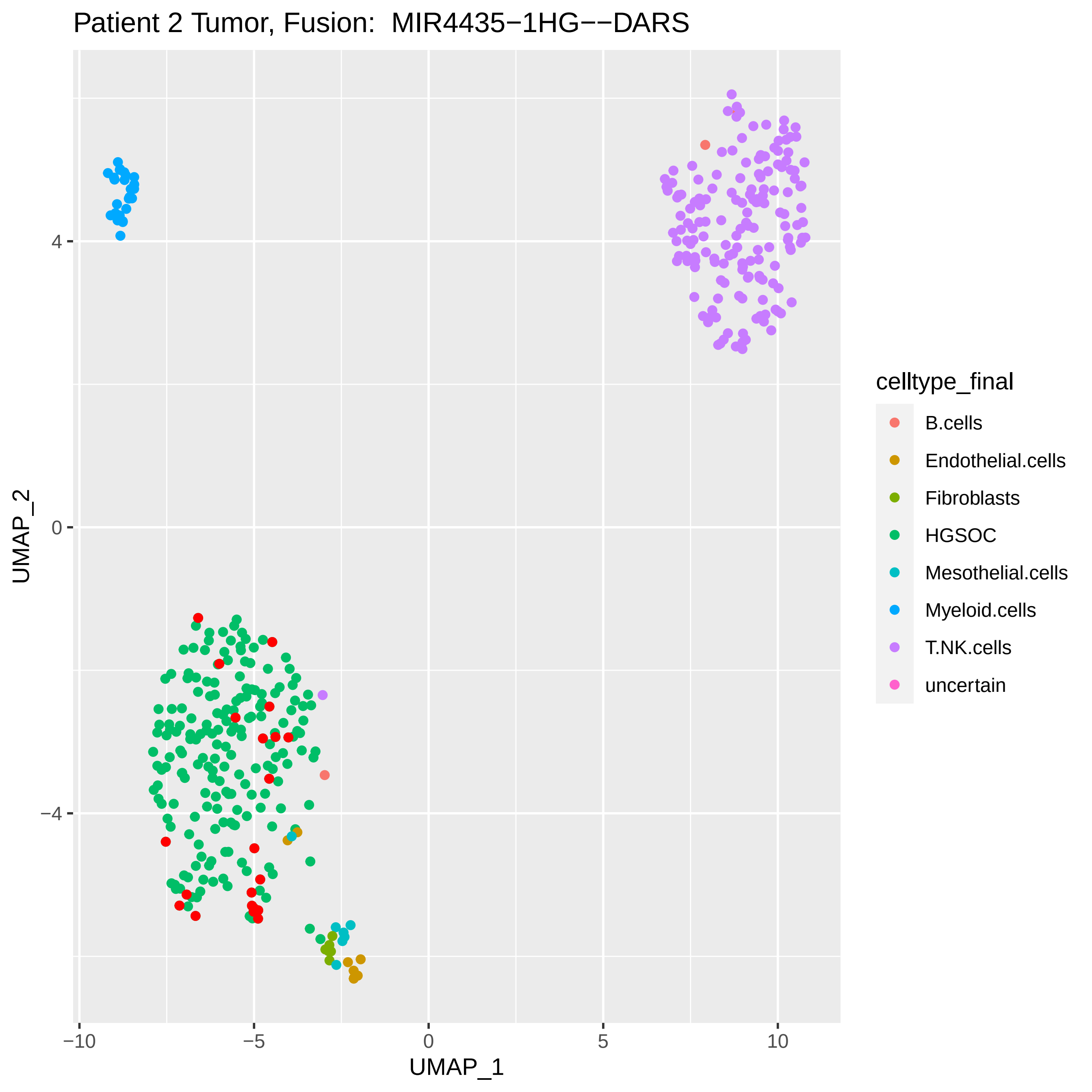

Patient 2 Tumor, Fusion: MIR4435−1HG−−DARS
UMAP_2
UMAP_1
4
0
−4
−10
−5
0
5
10
celltype_final
B.cells
Endothelial.cells
Fibroblasts
HGSOC
Mesothelial.cells
Myeloid.cells
T.NK.cells
uncertain

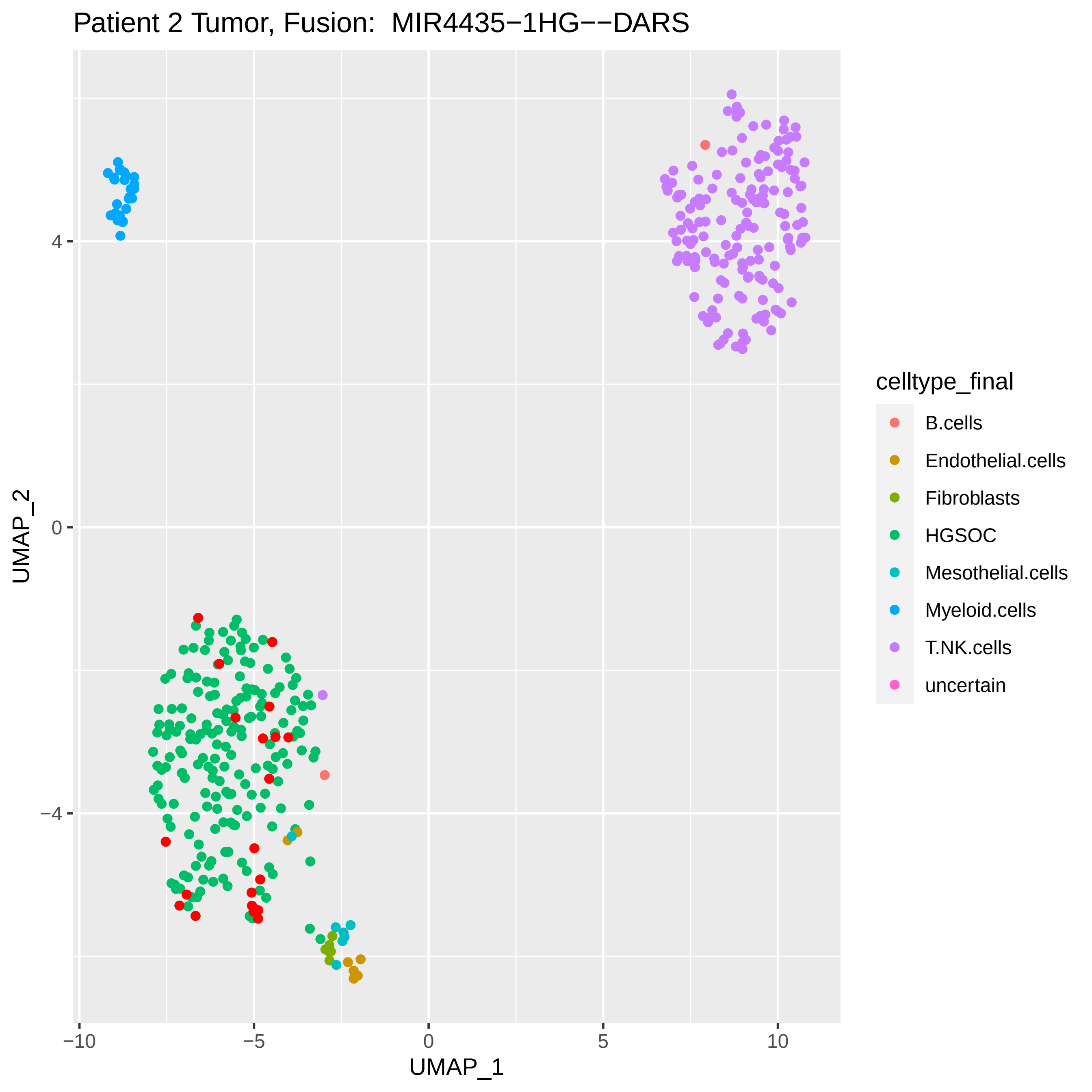
Patient 2 Tumor, Fusion: MIR4435−1HG−−DARS
UMAP_2
4
0
−4
UMAP_1
−10
−5
0
5
10
celltype_final
B.cells
Endothelial.cells
Fibroblasts
HGSOC
Mesothelial.cells
Myeloid.cells
T.NK.cells
uncertain

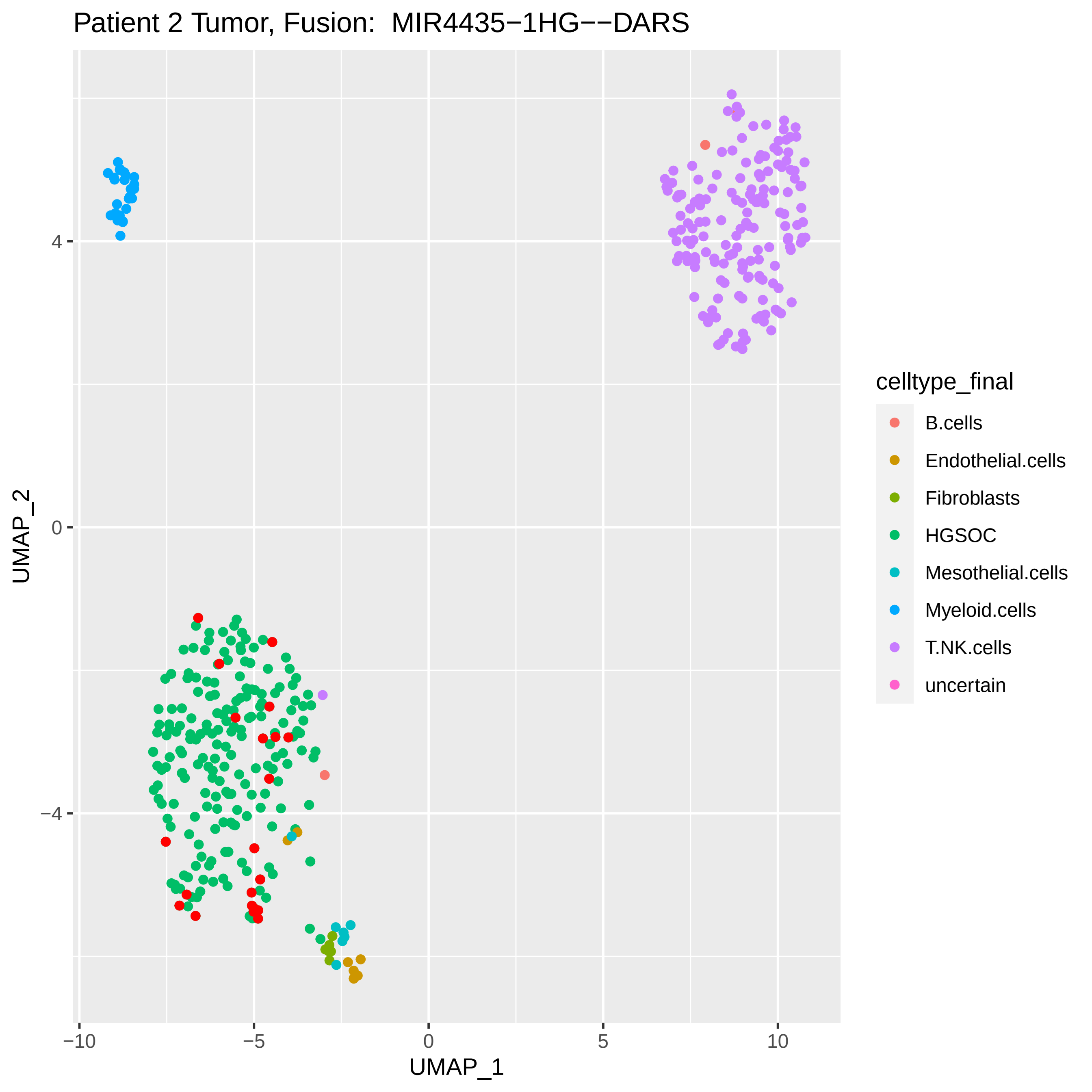

Patient 2 Tumor, Fusion: MIR4435−1HG−−DARS
UMAP_2
UMAP_1
4
0
−4
−10
−5
0
5
10
celltype_final
B.cells
Endothelial.cells
Fibroblasts
HGSOC
Mesothelial.cells
Myeloid.cells
T.NK.cells
uncertain

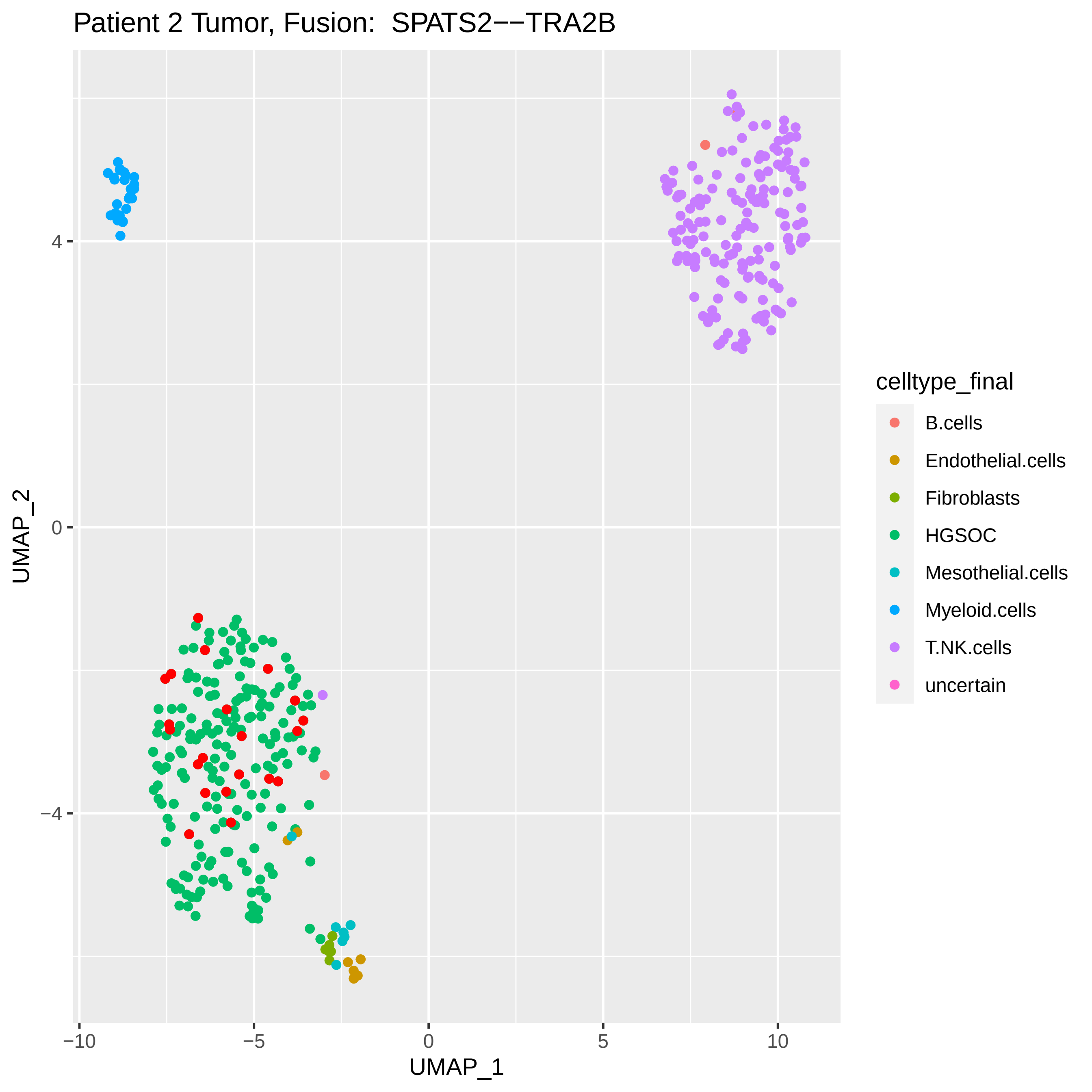
Patient 2 Tumor, Fusion: SPATS2--TRA2B
UMAP_2
UMAP_1
4
0
−4
−10
−5
0
5
10
celltype_final
B.cells
Endothelial.cells
Fibroblasts
HGSOC
Mesothelial.cells
Myeloid.cells
T.NK.cells
uncertain

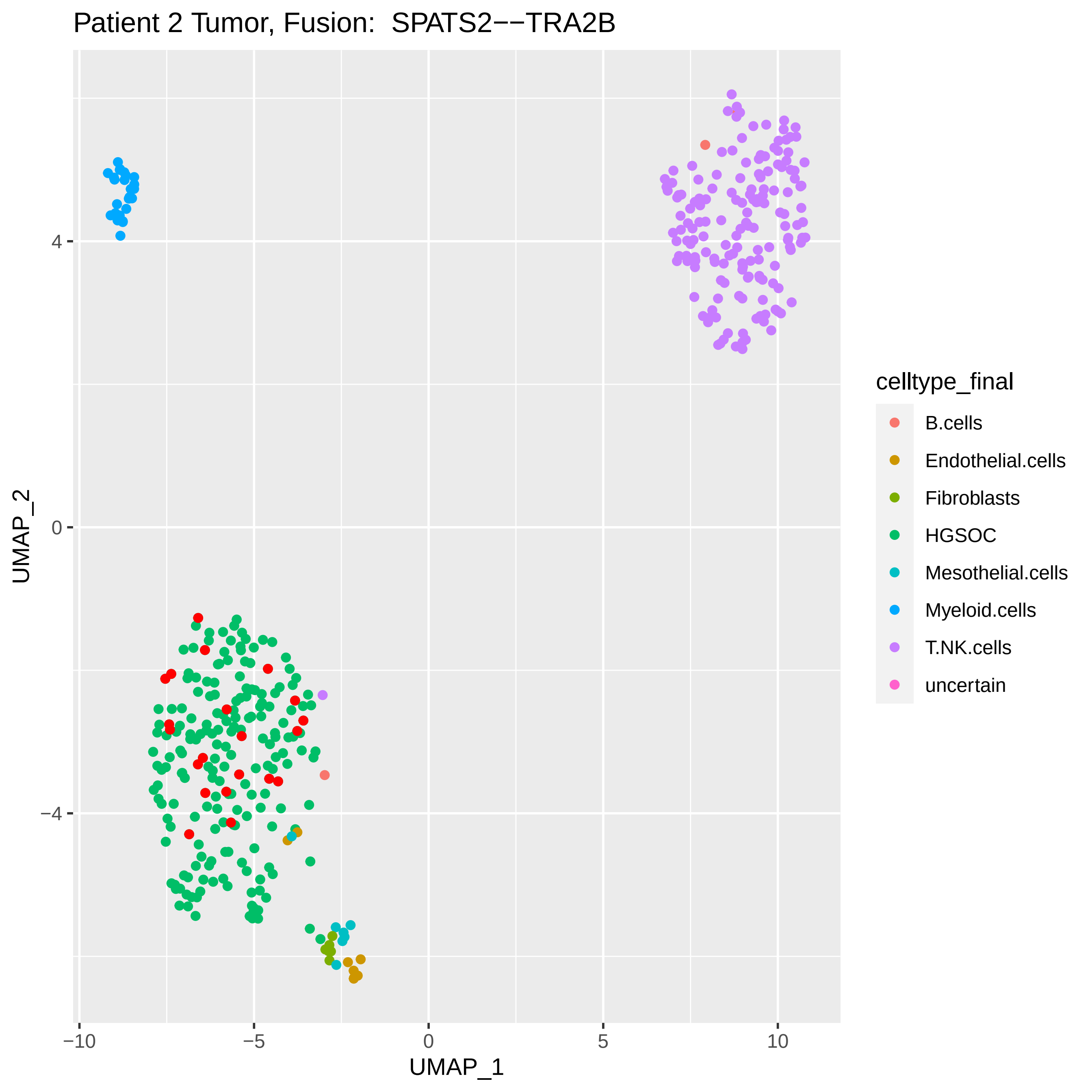

Patient 2 Tumor, Fusion: SPATS2--TRA2B
UMAP_2
UMAP_1
4
0
−4
−10
−5
0
5
10
celltype_final
B.cells
Endothelial.cells
Fibroblasts
HGSOC
Mesothelial.cells
Myeloid.cells
T.NK.cells
uncertain

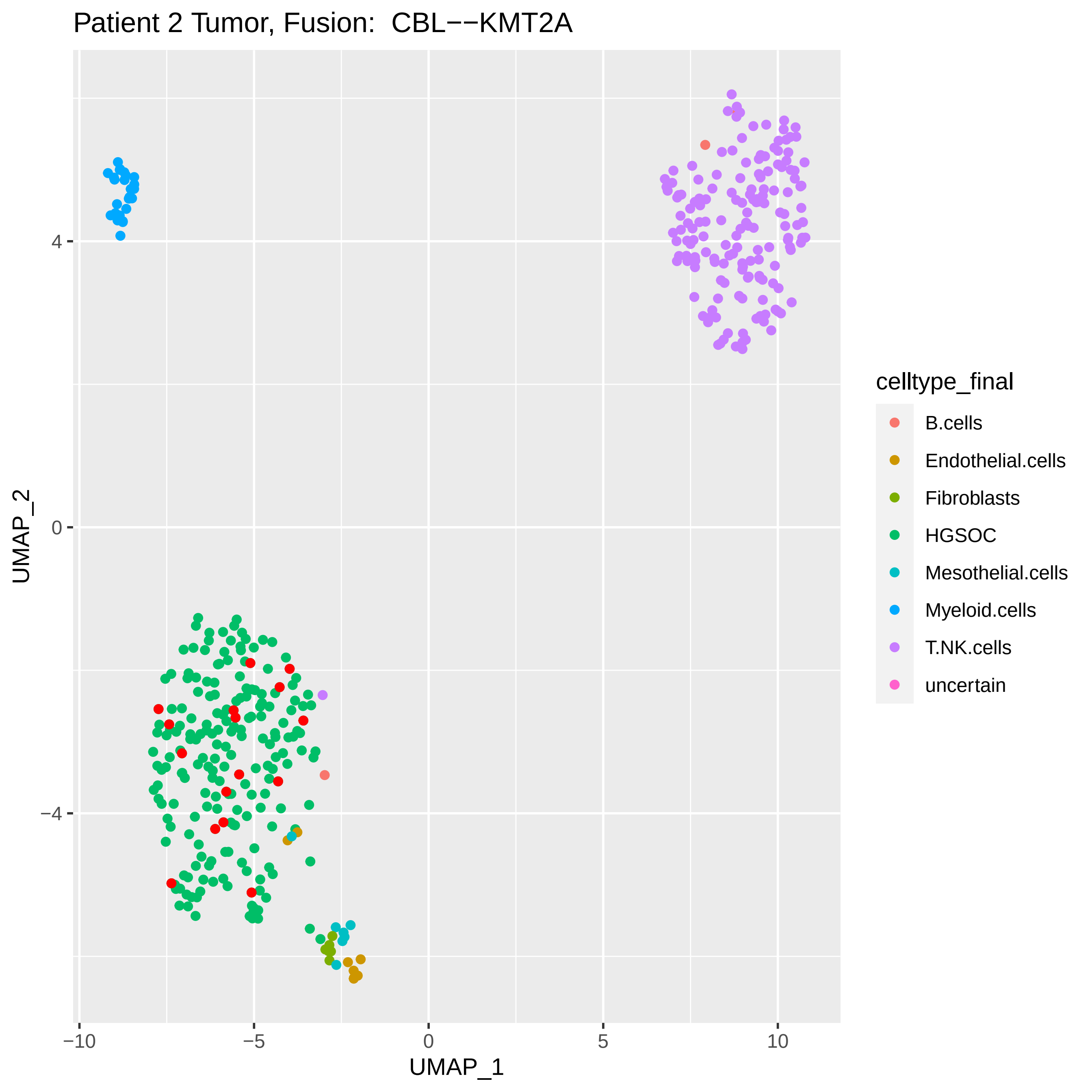
Patient 2 Tumor, Fusion: CBL−−KMT2A
UMAP_2
UMAP_1
4
0
−4
−10
−5
0
5
10
celltype_final
B.cells
Endothelial.cells
Fibroblasts
HGSOC
Mesothelial.cells
Myeloid.cells
T.NK.cells
uncertain

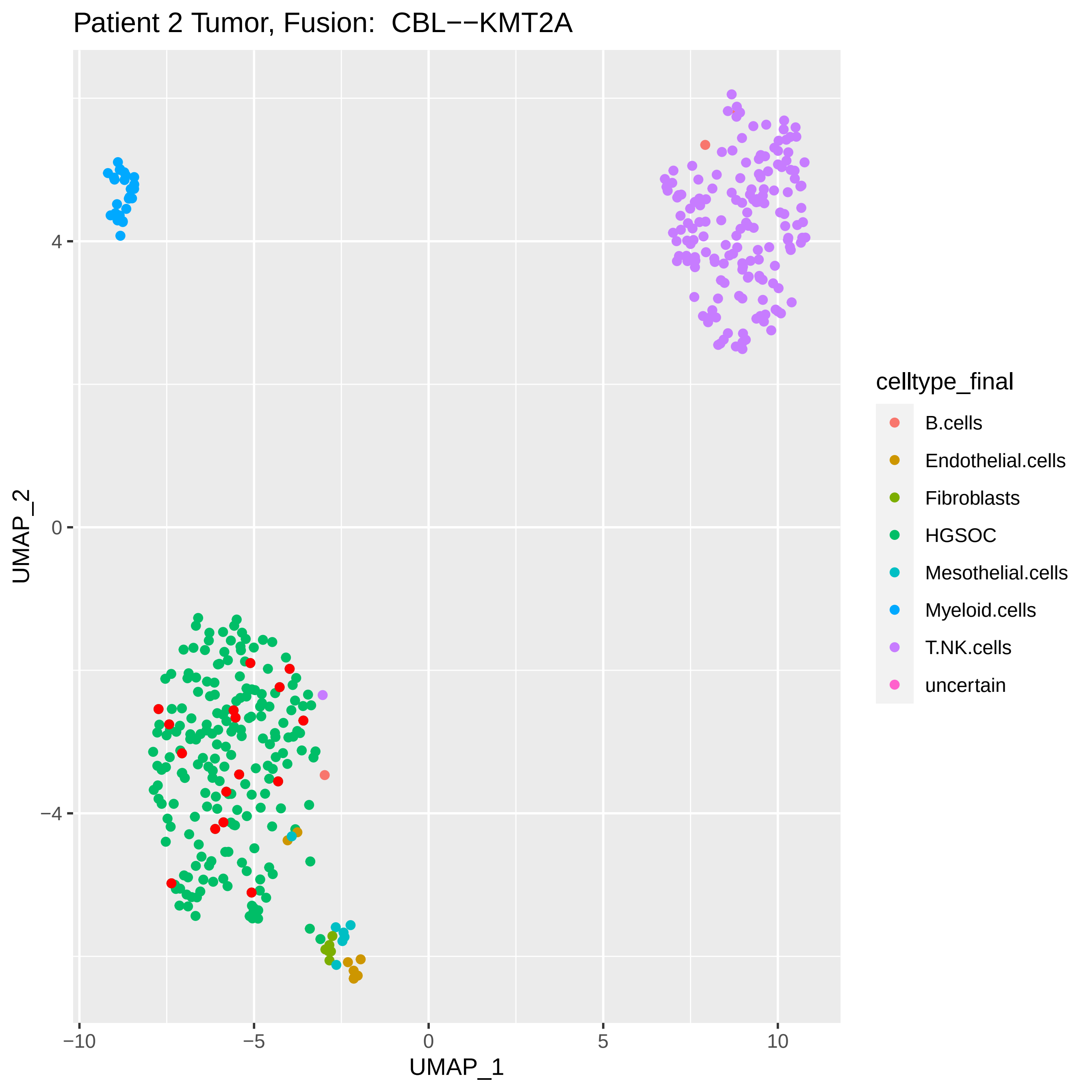

Patient 2 Tumor, Fusion: CBL−−KMT2A
UMAP_2
4
0
−4
UMAP_1
−10
−5
0
5
10
celltype_final
B.cells
Endothelial.cells
Fibroblasts
HGSOC
Mesothelial.cells
Myeloid.cells
T.NK.cells
uncertain

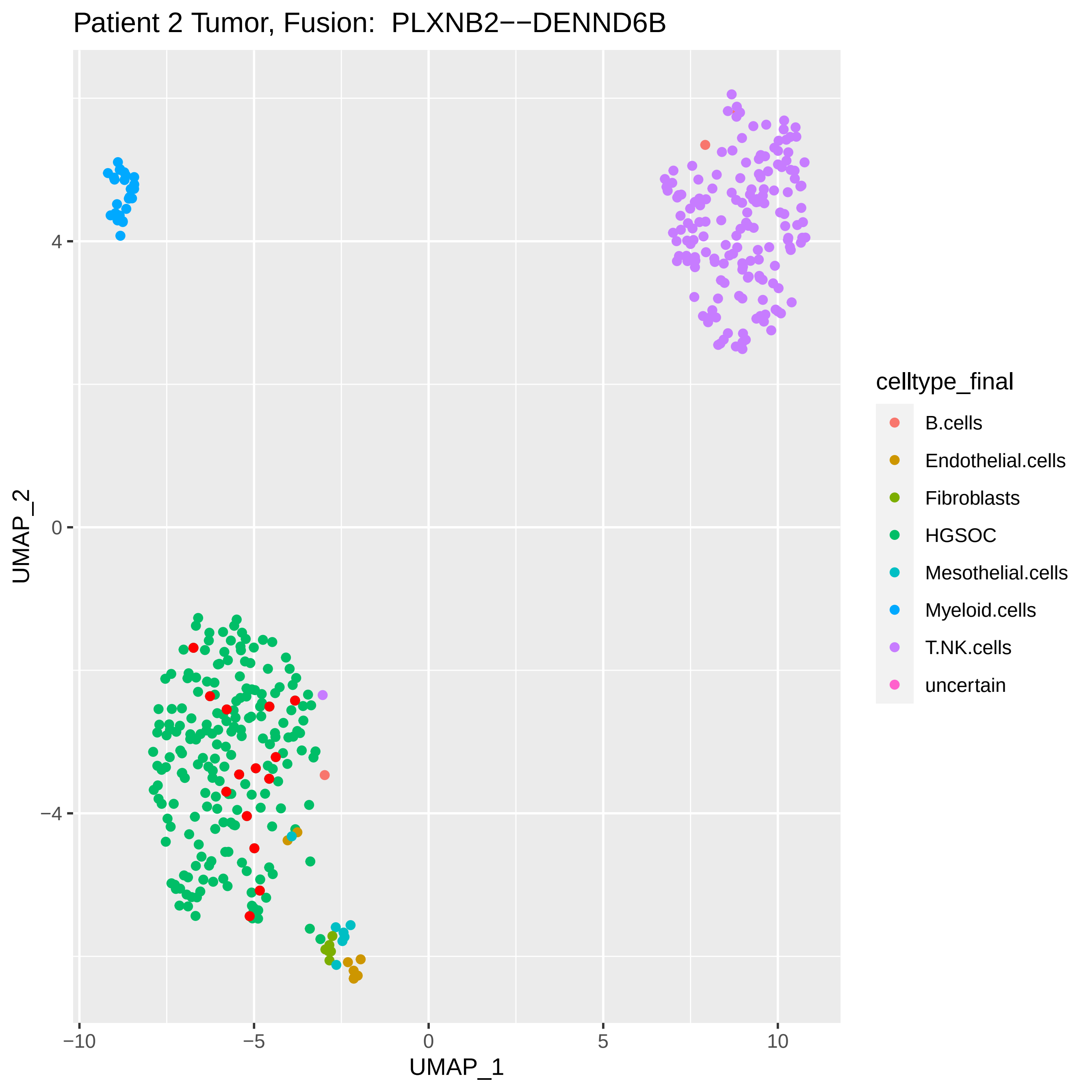

Patient 2 Tumor, Fusion: PLXNB2−−DENND6B
UMAP_2
UMAP_1
4
0
−4
−10
−5
0
5
10
celltype_final
B.cells
Endothelial.cells
Fibroblasts
HGSOC
Mesothelial.cells
Myeloid.cells
T.NK.cells
uncertain

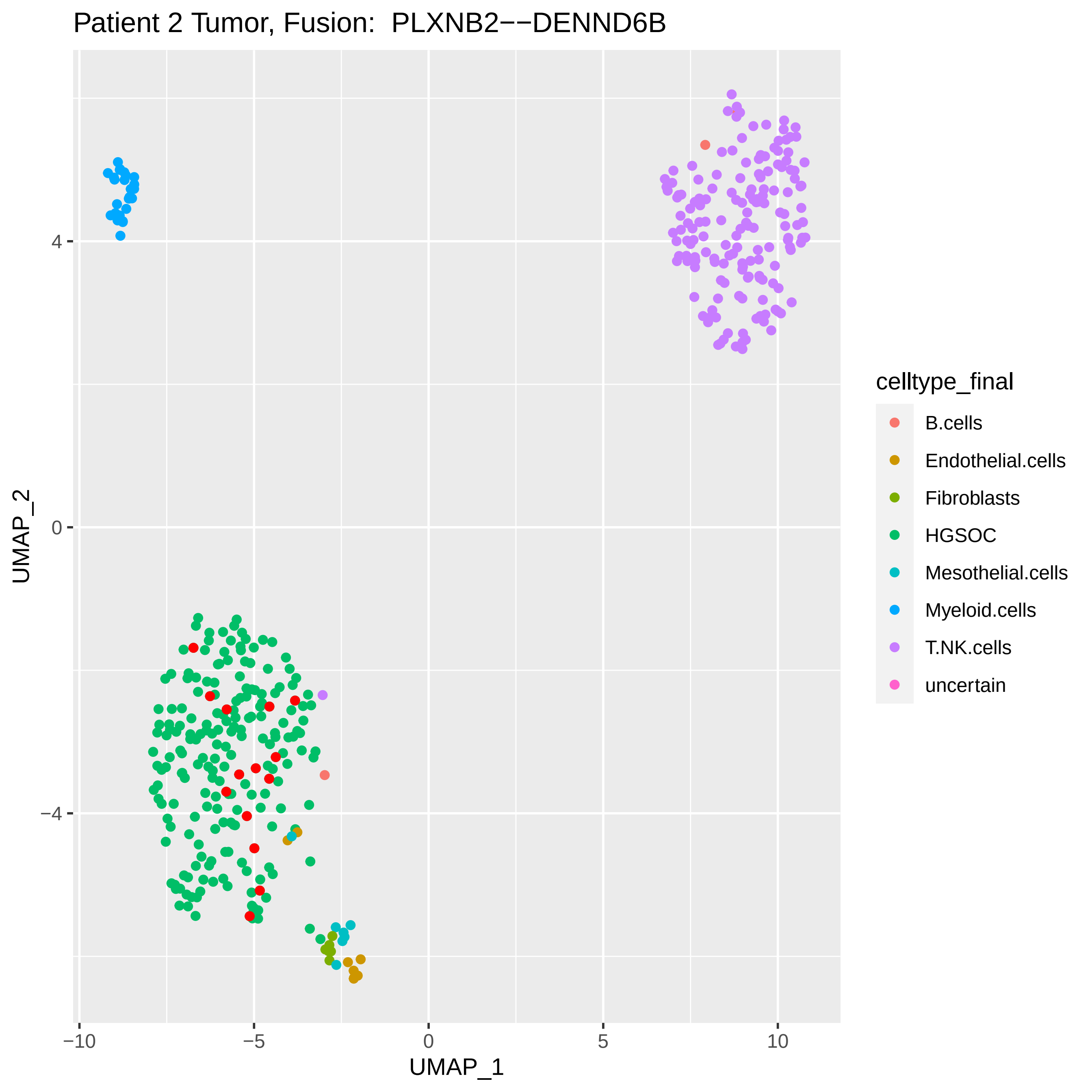

Patient 2 Tumor, Fusion: PLXNB2−−DENND6B
UMAP_2
UMAP_1
4
0
−4
−10
−5
0
5
10
celltype_final
B.cells
Endothelial.cells
Fibroblasts
HGSOC
Mesothelial.cells
Myeloid.cells
T.NK.cells
uncertain

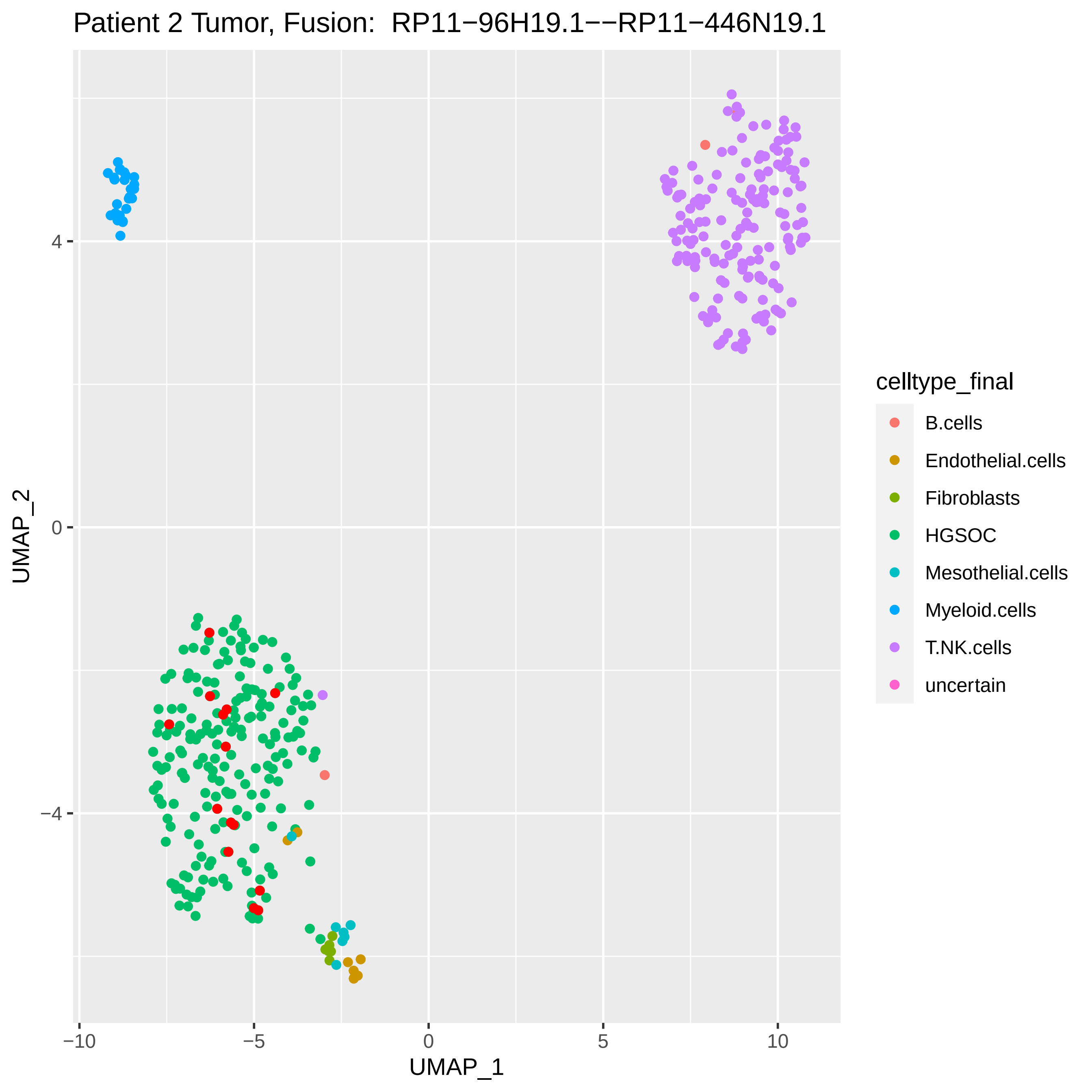

Patient 2 Tumor, Fusion: RP11−96H19.1−−RP11−446N19.1
UMAP_2
4
0
−4
UMAP_1
−10
−5
0
5
10
celltype_final
B.cells
Endothelial.cells
Fibroblasts
HGSOC
Mesothelial.cells
Myeloid.cells
T.NK.cells
uncertain

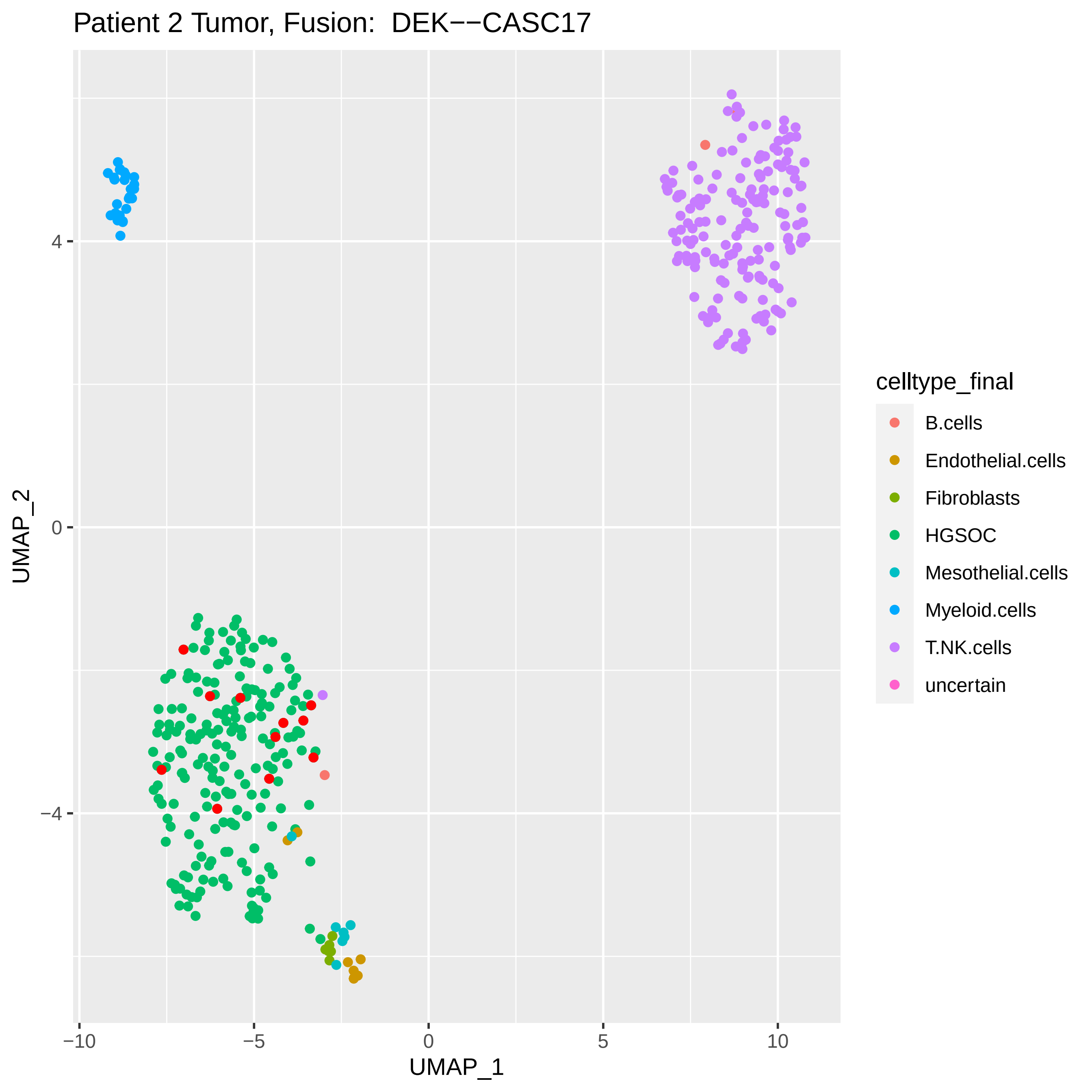
Patient 2 Tumor, Fusion: DEK−−CASC17
UMAP_2
4
0
−4
−10
−5
0
5
10
UMAP_1
celltype_final
B.cells
Endothelial.cells
Fibroblasts
HGSOC
Mesothelial.cells
Myeloid.cells
T.NK.cells
uncertain

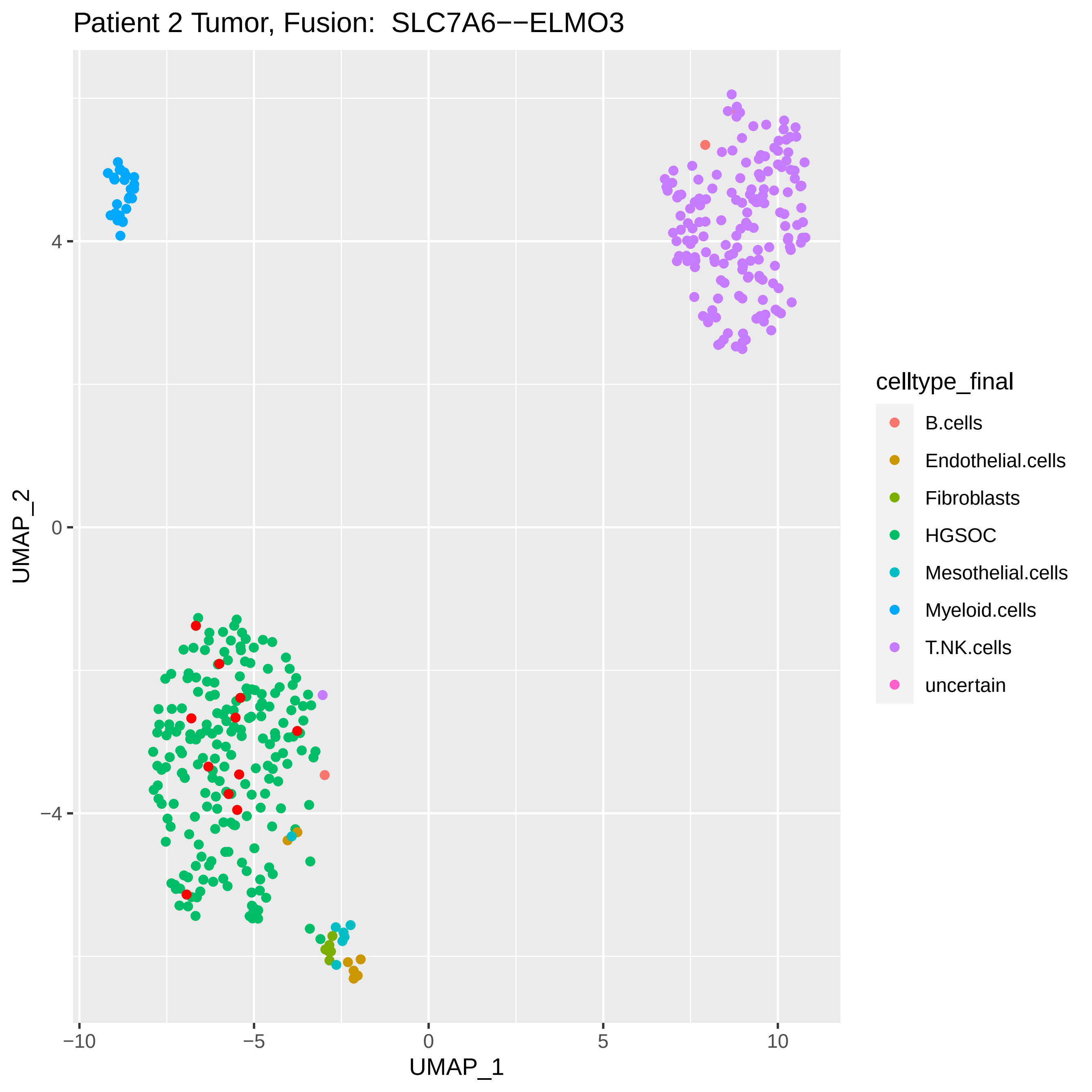
Patient 2 Tumor, Fusion: SLC7A6−−ELMO3
UMAP_2
UMAP_1
4
0
−4
−10
−5
0
5
10
celltype_final
B.cells
Endothelial.cells
Fibroblasts
HGSOC
Mesothelial.cells
Myeloid.cells
T.NK.cells
uncertain

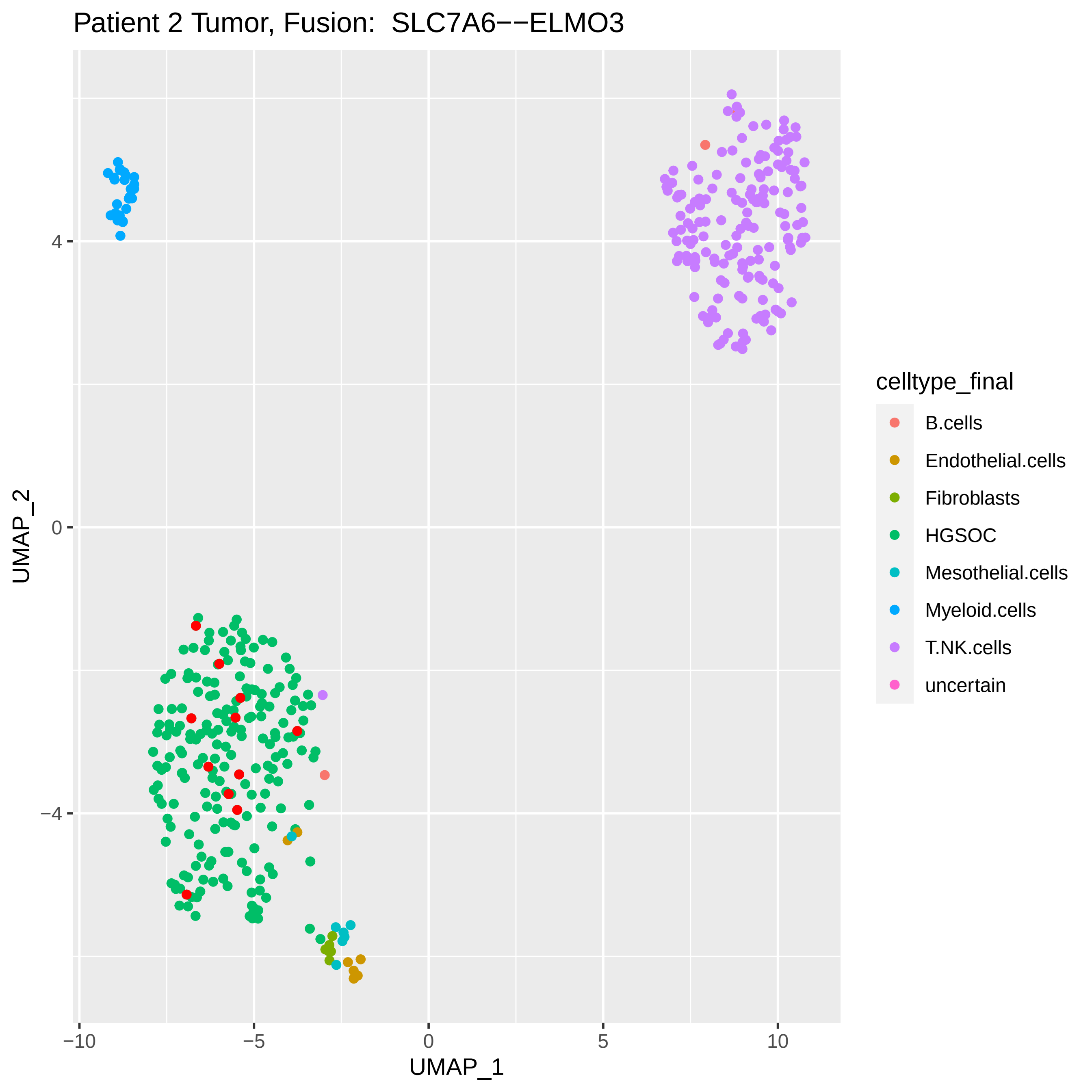
Patient 2 Tumor, Fusion: SLC7A6−−ELMO3
UMAP_2
4
0
−4
−10
−5
0
5
10
UMAP_1
celltype_final
B.cells
Endothelial.cells
Fibroblasts
HGSOC
Mesothelial.cells
Myeloid.cells
T.NK.cells
uncertain

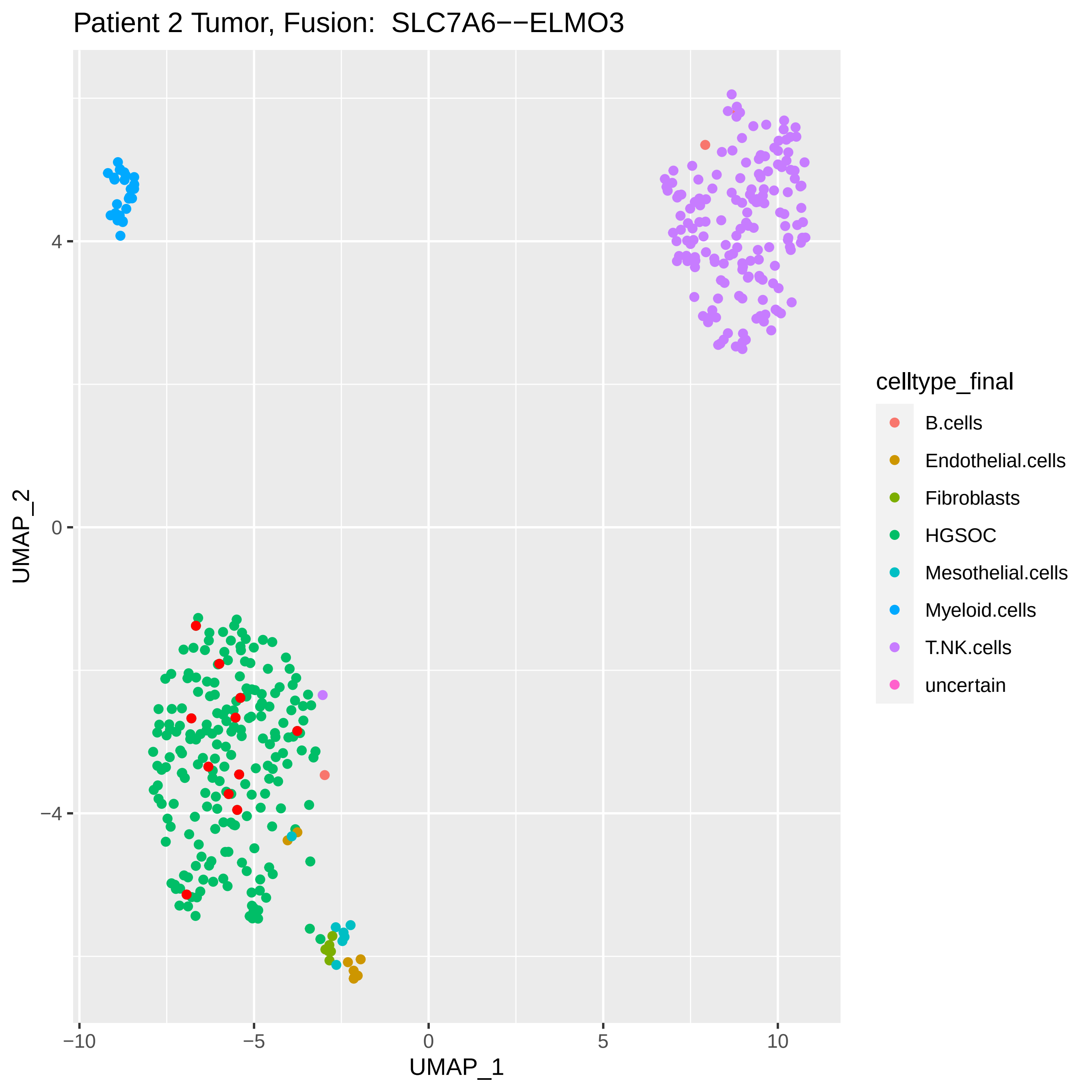

Patient 2 Tumor, Fusion: SLC7A6--ELMO3
UMAP_2
4
0
−4
−10
−5
0
5
10
UMAP_1
celltype_final
B.cells
Endothelial.cells
Fibroblasts
HGSOC
Mesothelial.cells
Myeloid.cells
T.NK.cells
uncertain

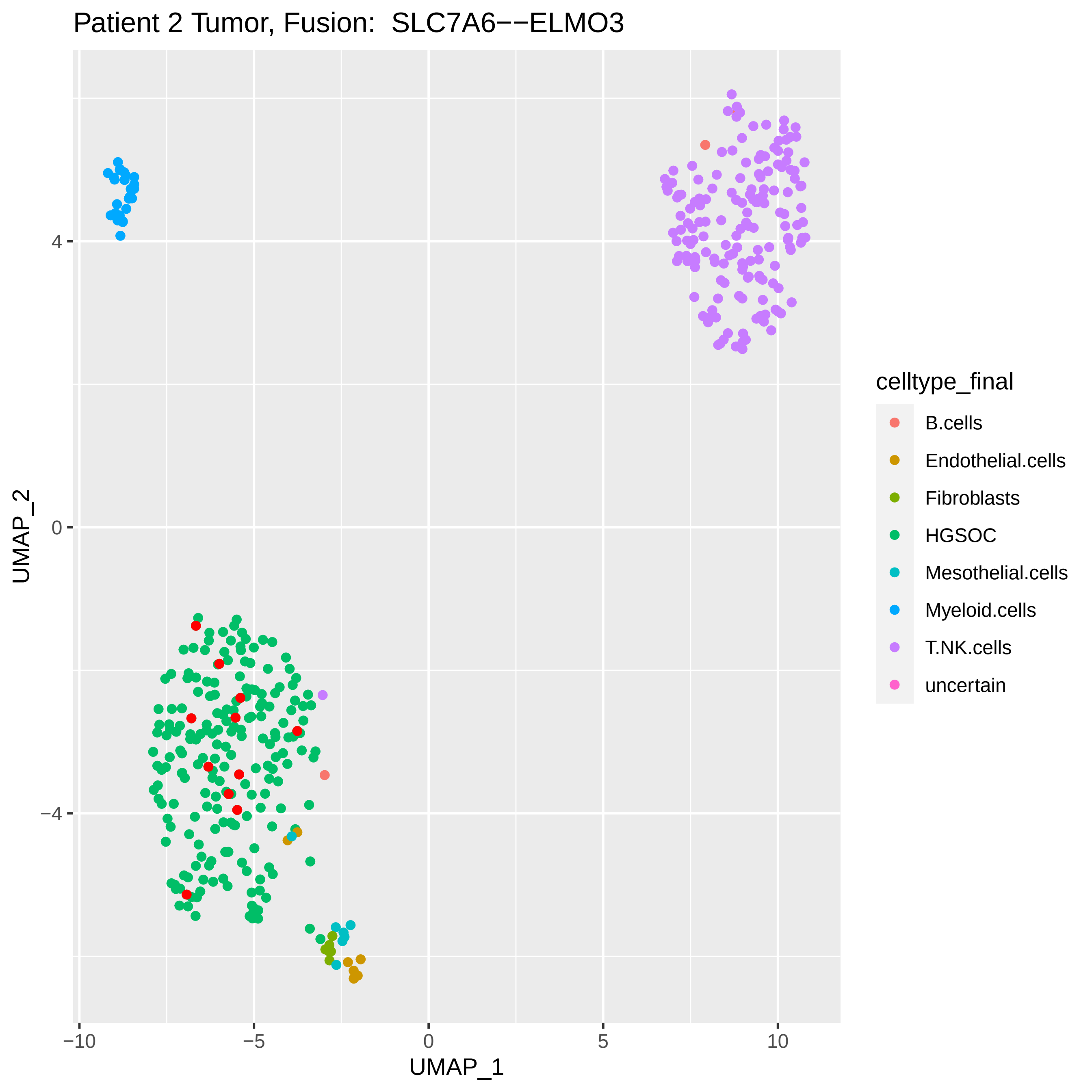

Patient 2 Tumor, Fusion: SLC7A6−−ELMO3
UMAP_2
UMAP_1
4
0
−4
−10
−5
0
5
10
celltype_final
B.cells
Endothelial.cells
Fibroblasts
HGSOC
Mesothelial.cells
Myeloid.cells
T.NK.cells
uncertain

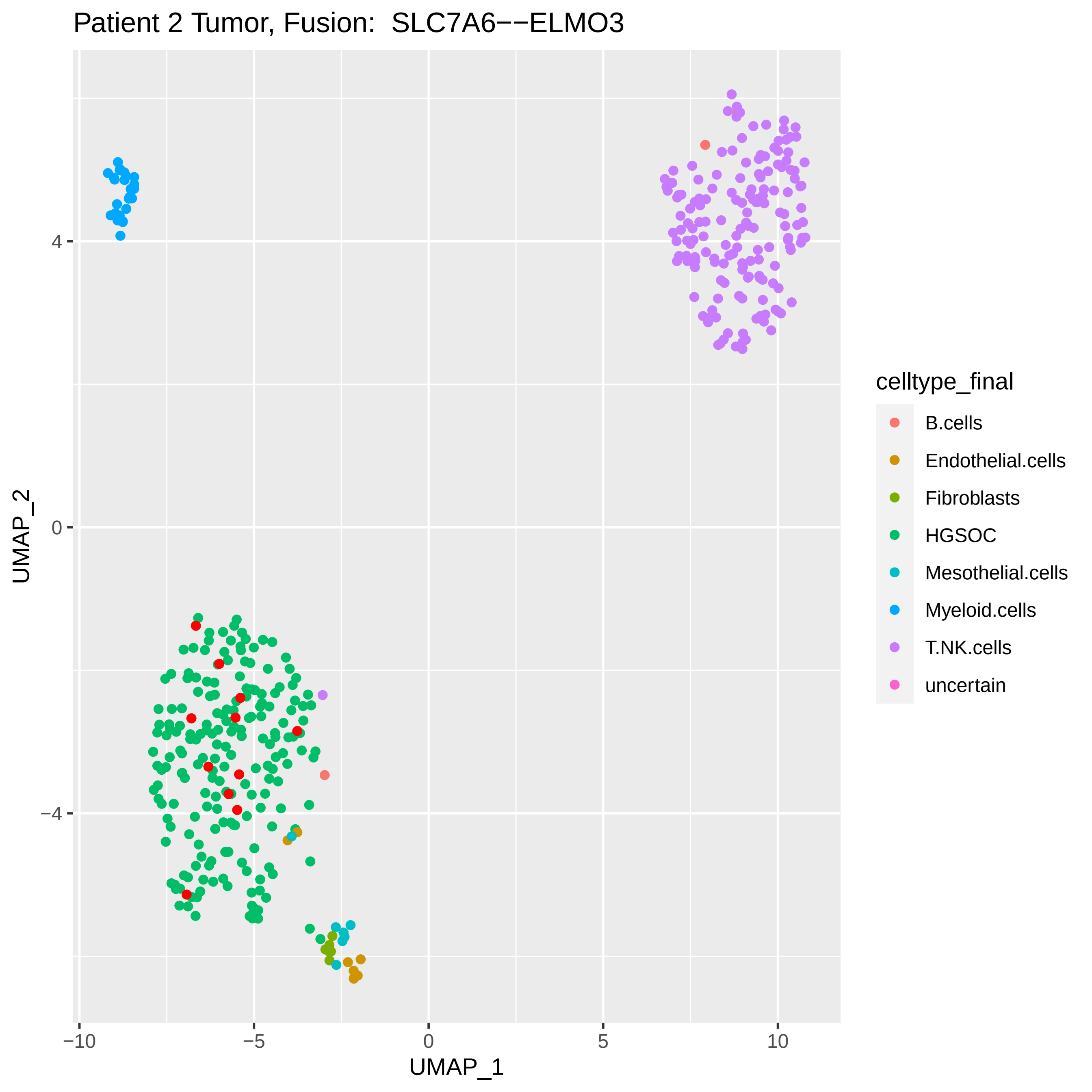
Patient 2 Tumor, Fusion: SLC7A6--ELMO3
UMAP_2
4
0
−4
−10
−5
0
5
10
UMAP_1
celltype_final
B.cells
Endothelial.cells
Fibroblasts
HGSOC
Mesothelial.cells
Myeloid.cells
T.NK.cells
uncertain

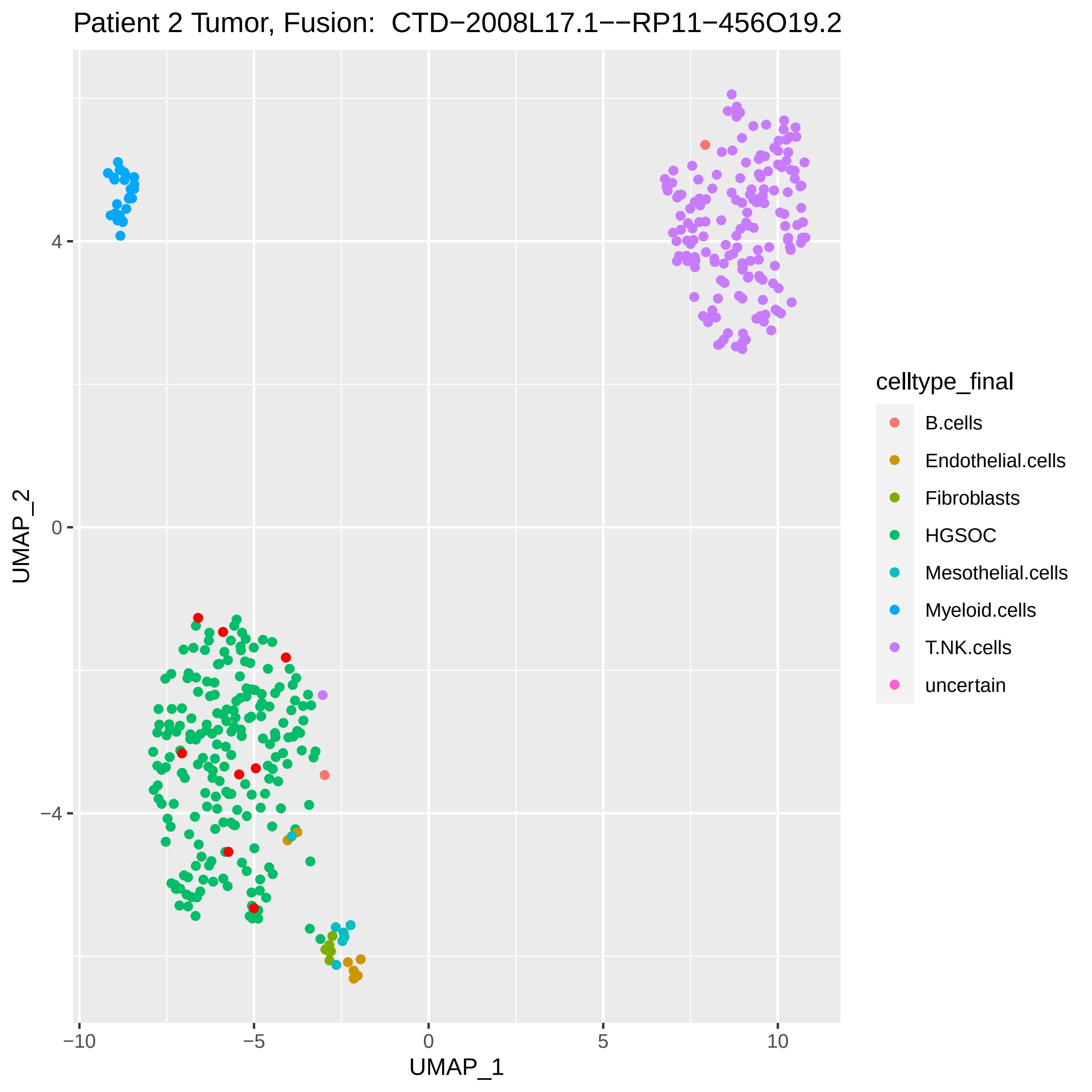
Patient 2 Tumor, Fusion: CTD−2008L17.1−−RP11−456O19.2
UMAP_2
UMAP_1
4
0
−4
−10
−5
0
5
10
celltype_final
B.cells
Endothelial.cells
Fibroblasts
HGSOC
Mesothelial.cells
Myeloid.cells
T.NK.cells
uncertain

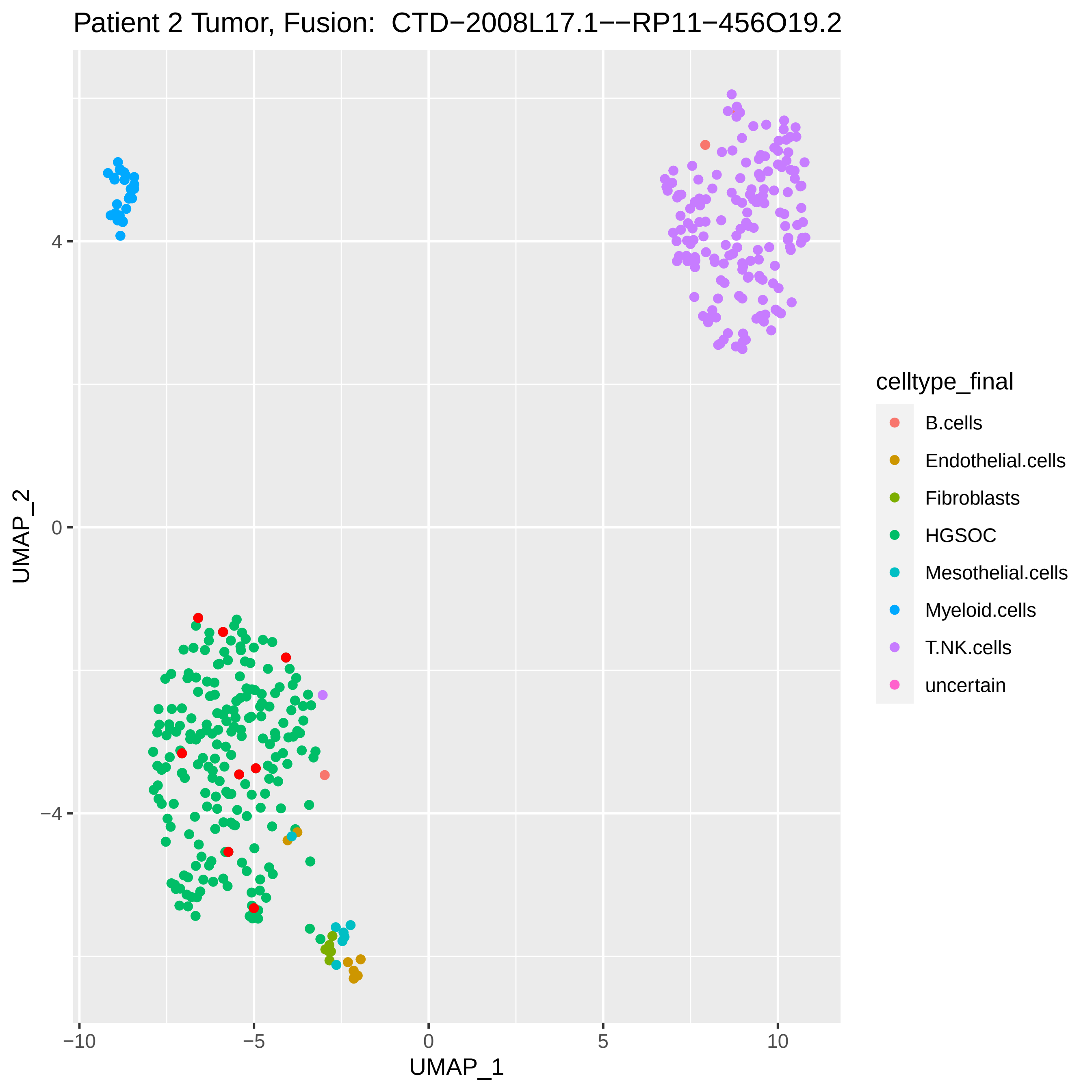
Patient 2 Tumor, Fusion: CTD−2008L17.1−−RP11−456O19.2
UMAP_2
UMAP_1
4
0
−4
−10
−5
0
5
10
celltype_final
B.cells
Endothelial.cells
Fibroblasts
HGSOC
Mesothelial.cells
Myeloid.cells
T.NK.cells
uncertain

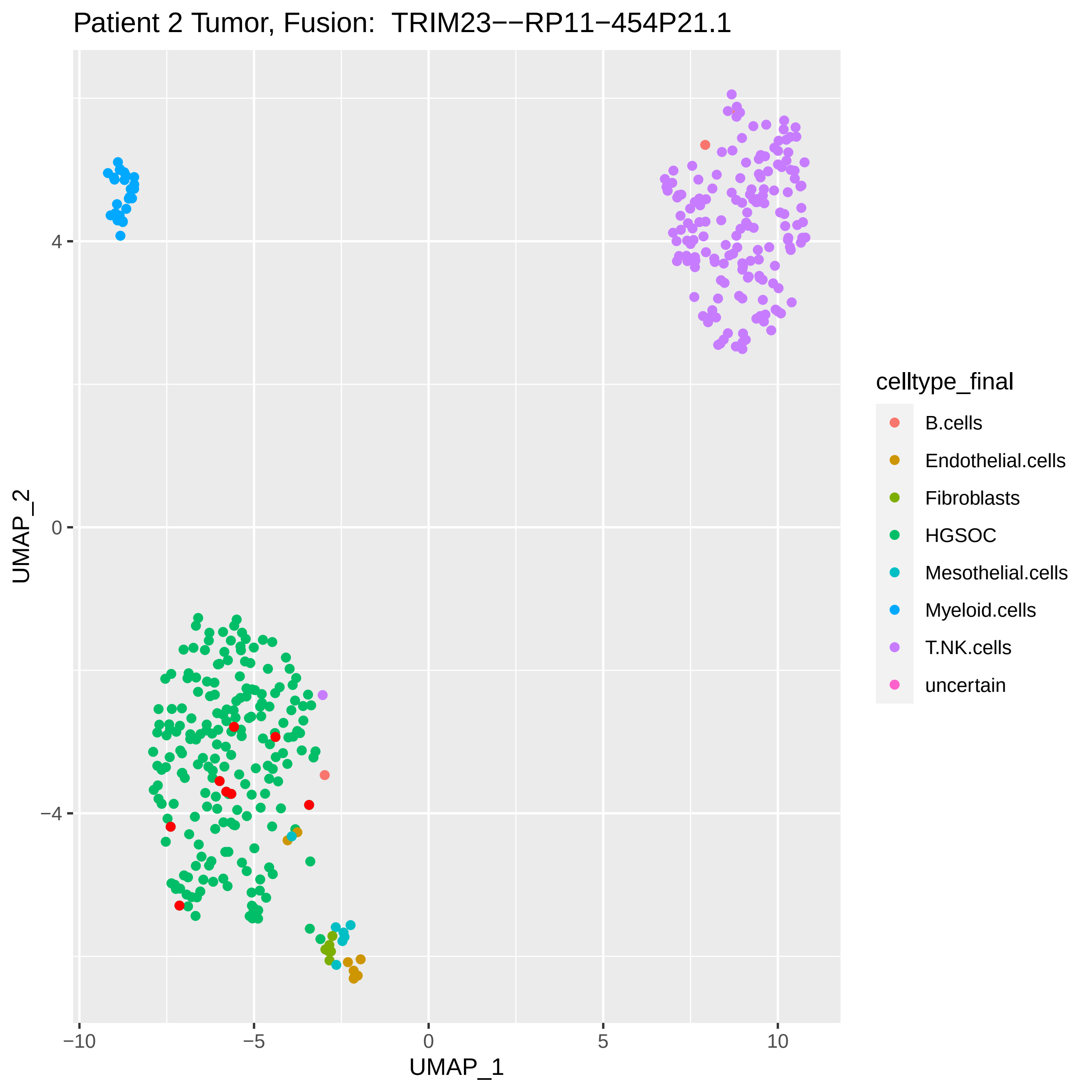
Patient 2 Tumor, Fusion: TRIM23−−RP11−454P21.1
UMAP_2
4
0
−4
UMAP_1
−10
−5
0
5
10
celltype_final
B.cells
Endothelial.cells
Fibroblasts
HGSOC
Mesothelial.cells
Myeloid.cells
T.NK.cells
uncertain

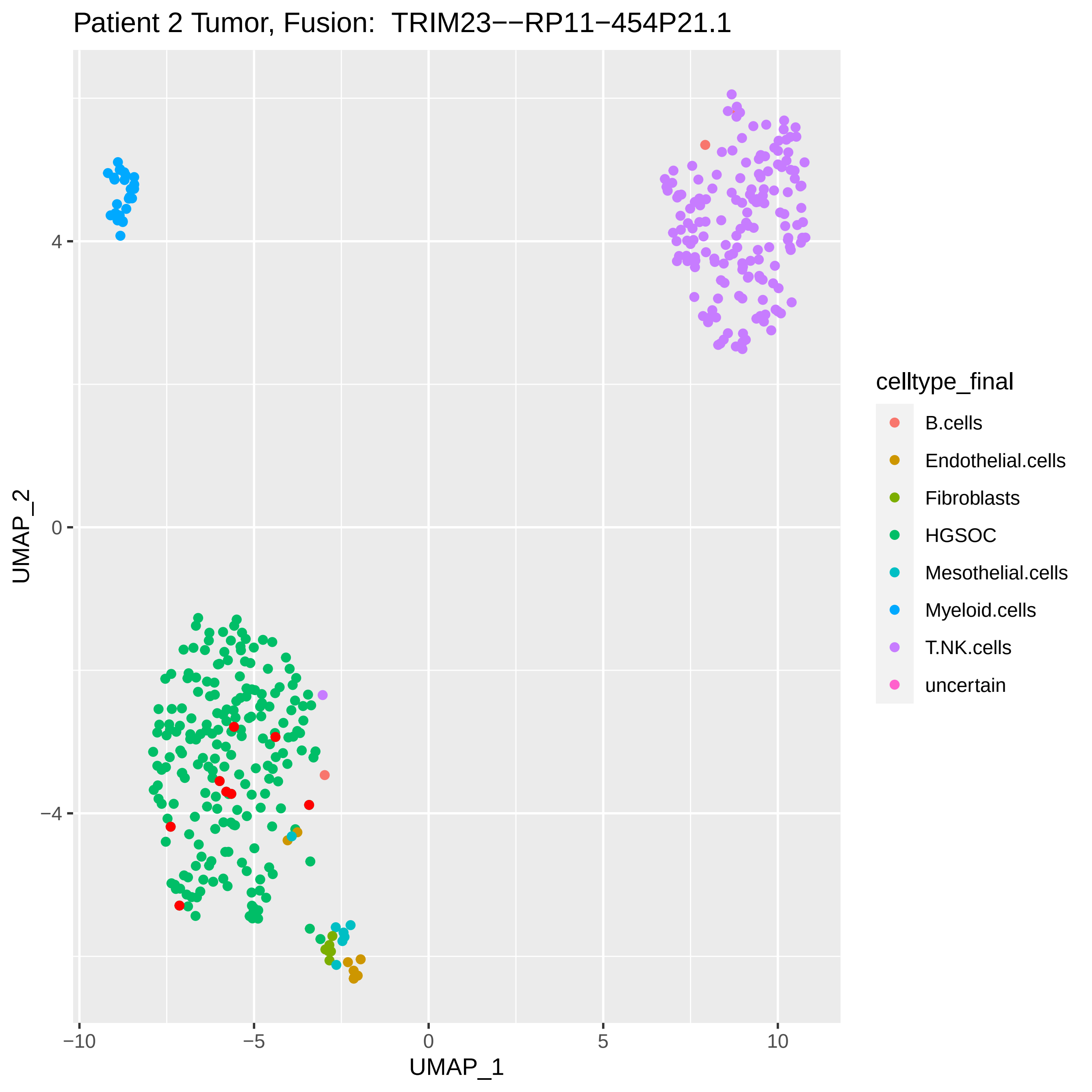

Patient 2 Tumor, Fusion: TRIM23−−RP11−454P21.1
UMAP_2
4
0
−4
UMAP_1
−10
−5
0
5
10
celltype_final
B.cells
Endothelial.cells
Fibroblasts
HGSOC
Mesothelial.cells
Myeloid.cells
T.NK.cells
uncertain

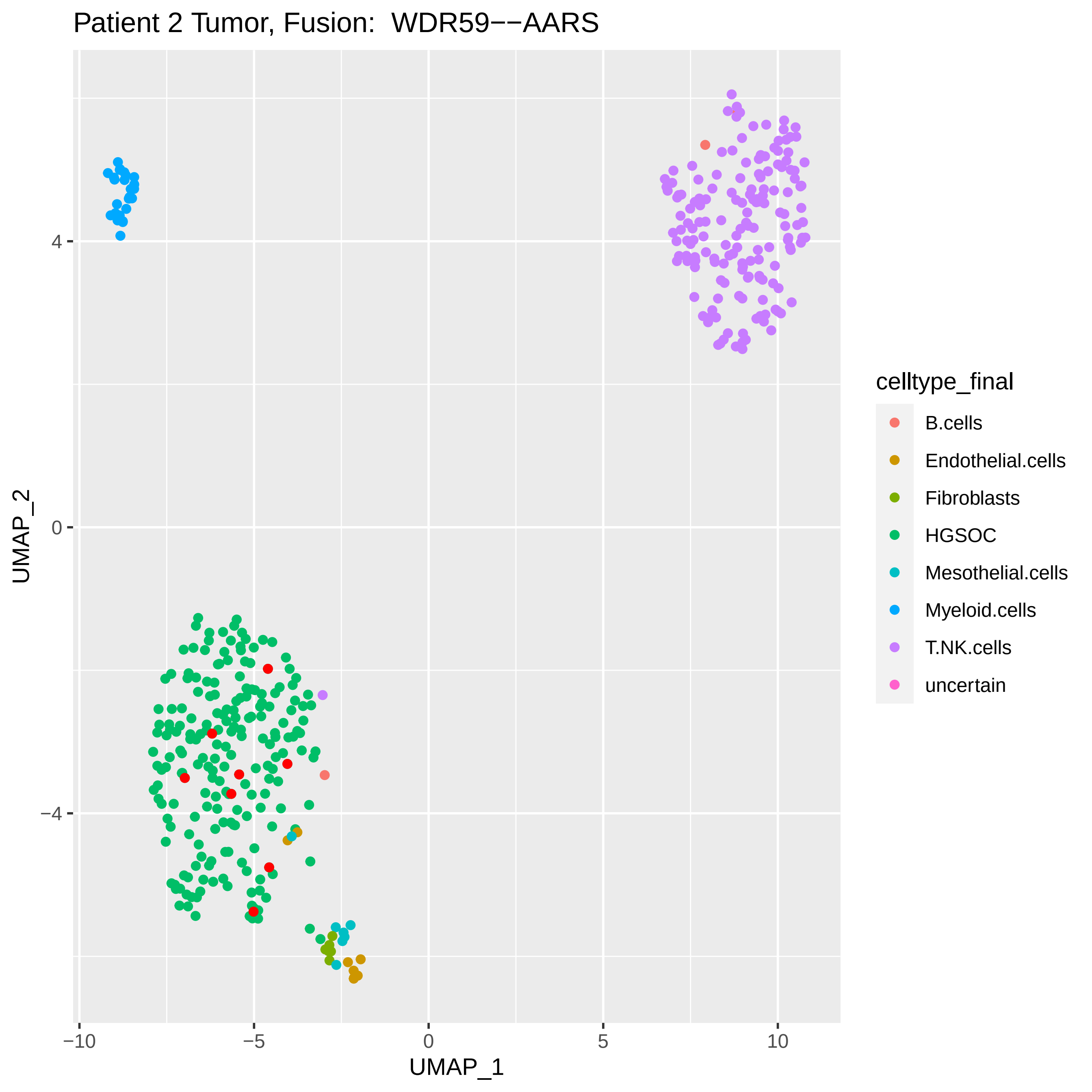

Patient 2 Tumor, Fusion: WDR59--AARS
UMAP_2
UMAP_1
4
0
−4
−10
−5
0
5
10
celltype_final
B.cells
Endothelial.cells
Fibroblasts
HGSOC
Mesothelial.cells
Myeloid.cells
T.NK.cells
uncertain

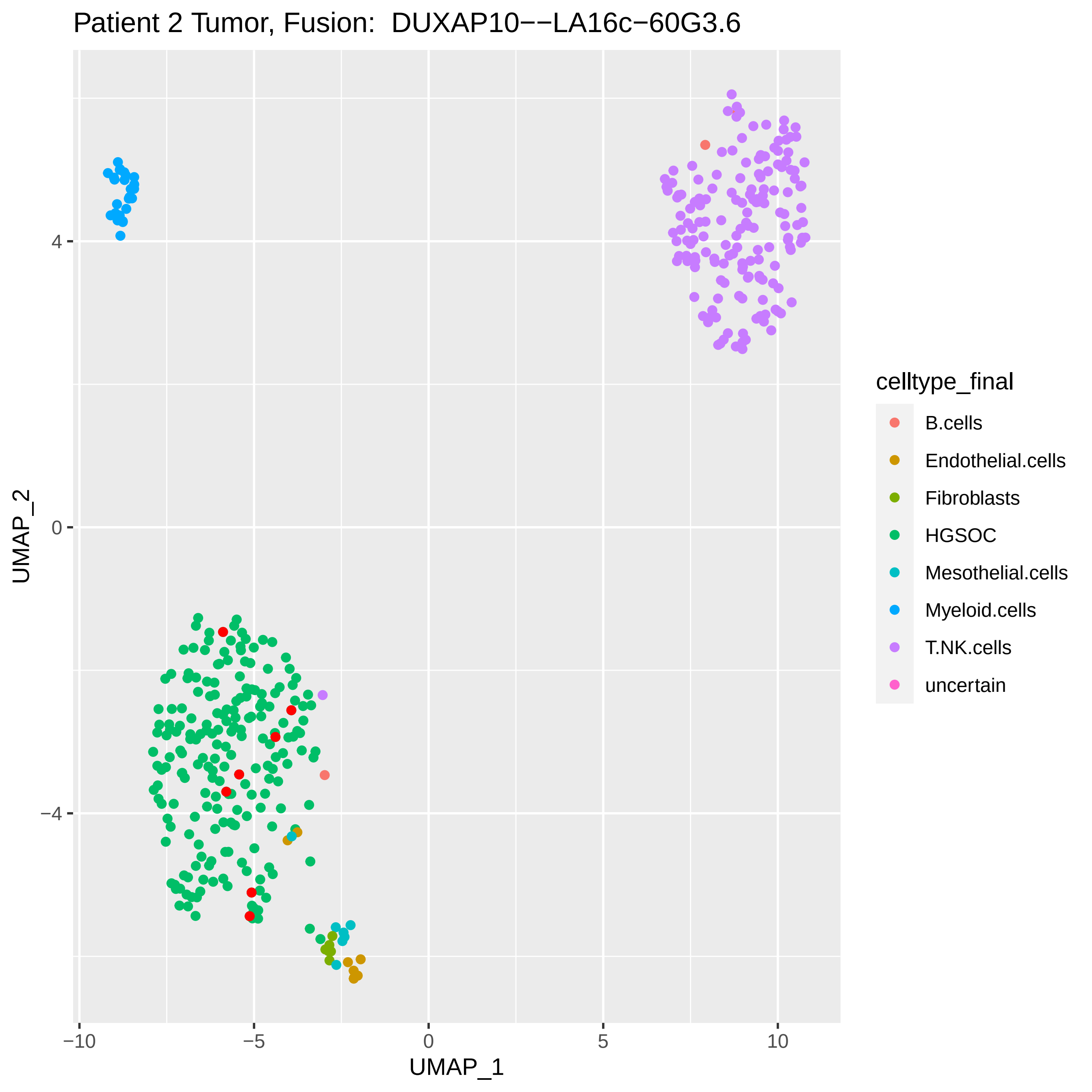

Patient 2 Tumor, Fusion: DUXAP10−−LA16c−60G3.6
UMAP_2
4
0
−4
−10
−5
0
5
10
UMAP_1
celltype_final
B.cells
Endothelial.cells
Fibroblasts
HGSOC
Mesothelial.cells
Myeloid.cells
T.NK.cells
uncertain

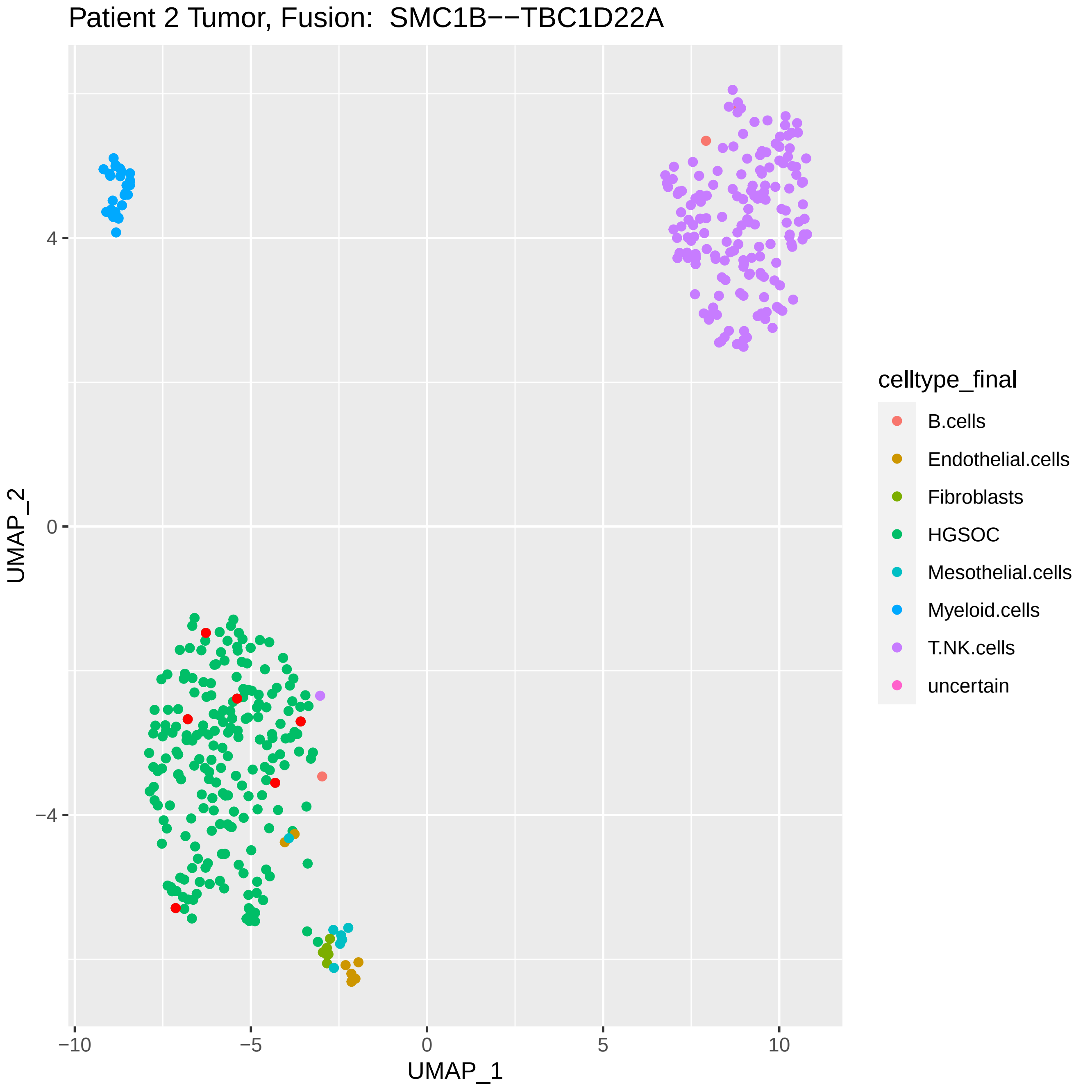
Patient 2 Tumor, Fusion: SMC1B−−TBC1D22A
UMAP_2
UMAP_1
4
0
−4
−10
−5
0
5
10
celltype_final
B.cells
Endothelial.cells
Fibroblasts
HGSOC
Mesothelial.cells
Myeloid.cells
T.NK.cells
uncertain

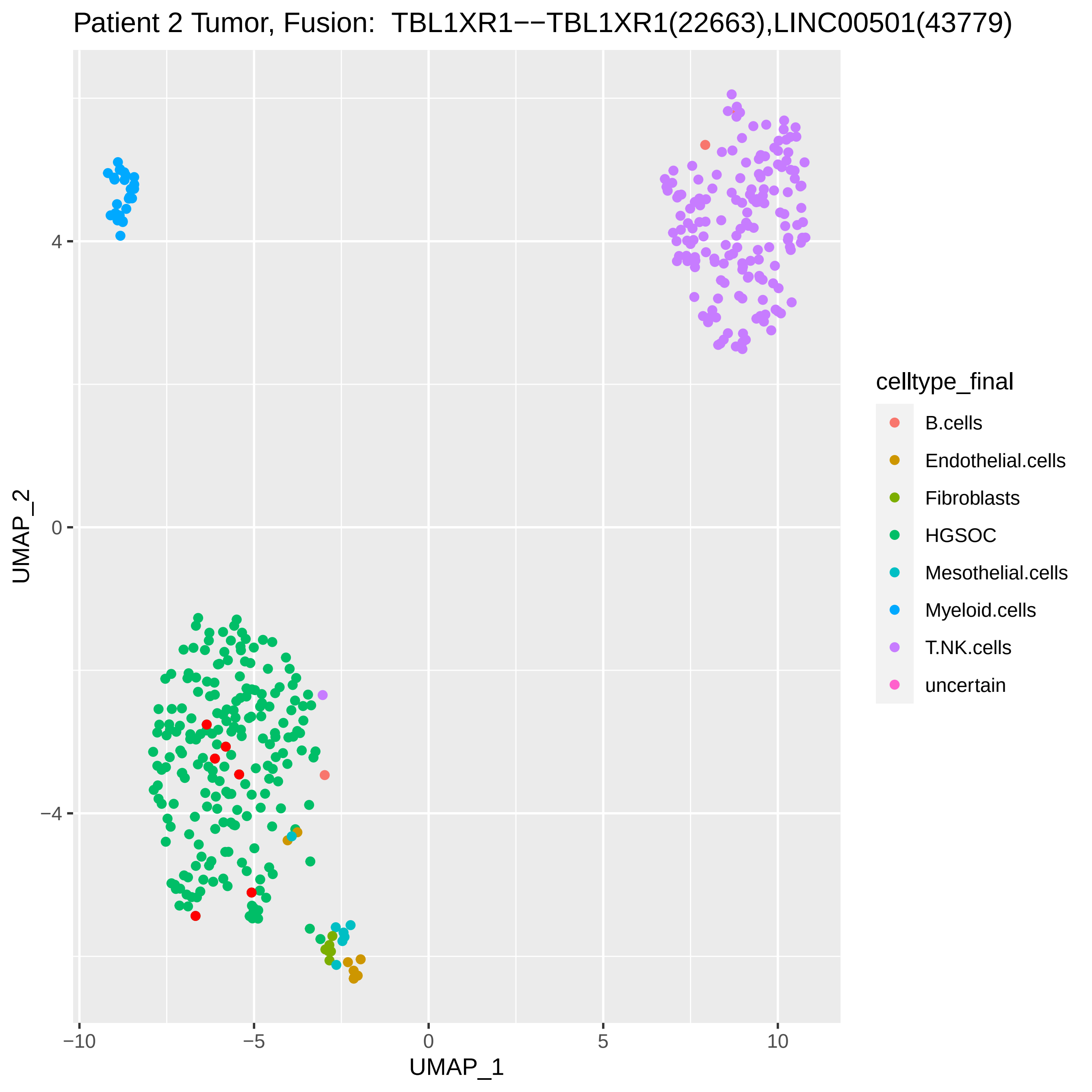

Patient 2 Tumor, Fusion: TBL1XR1−−TBL1XR1(22663),LINC00501(43779)
UMAP_2
UMAP_1
4
0
−4
−10
−5
0
5
10
celltype_final
B.cells
Endothelial.cells
Fibroblasts
HGSOC
Mesothelial.cells
Myeloid.cells
T.NK.cells
uncertain

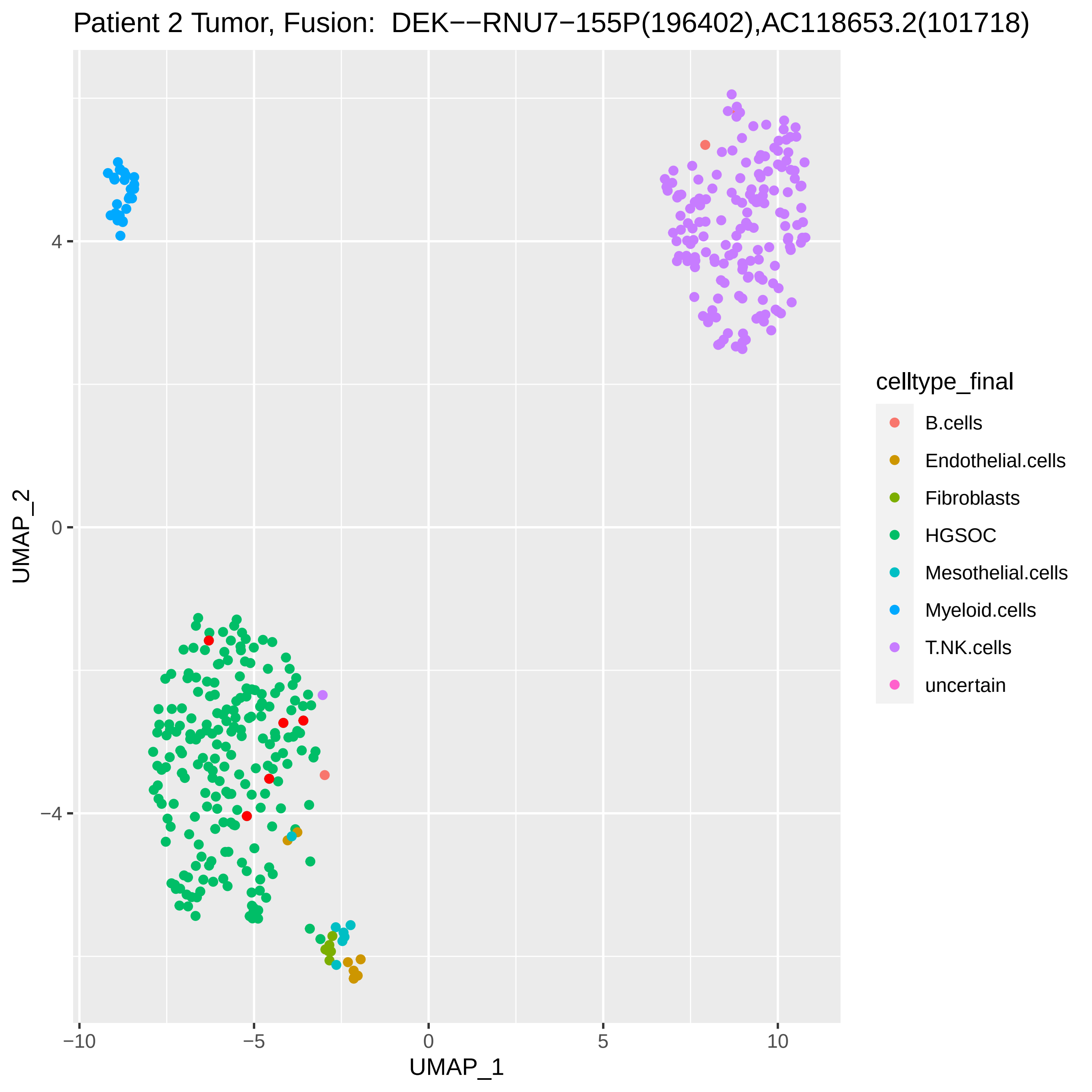
Patient 2 Tumor, Fusion: DEK−−RNU7−155P(196402),AC118653.2(101718)
UMAP_2
4
0
−4
UMAP_1
−10
−5
0
5
10
celltype_final
B.cells
Endothelial.cells
Fibroblasts
HGSOC
Mesothelial.cells
Myeloid.cells
T.NK.cells
uncertain
