# Supplementary material for: Accurate fusion transcript identification from long- and short-read isoform sequencing at bulk or single-cell resolution
Source: Genome Res. 2025 Apr;35(4):967–86. doi: 10.1101/gr.279200.124 (PMC12047241; doi:10.1101/gr.279200.124)
Supplement: Supplement 12 [file Patient3_Tum.fusions_of_interest.pdf › CTAT-LRF-Paper/4.SingleCellFusions/4b.sc_HGSOC/Patient3_Tum.fusions_of_interest.pdf]

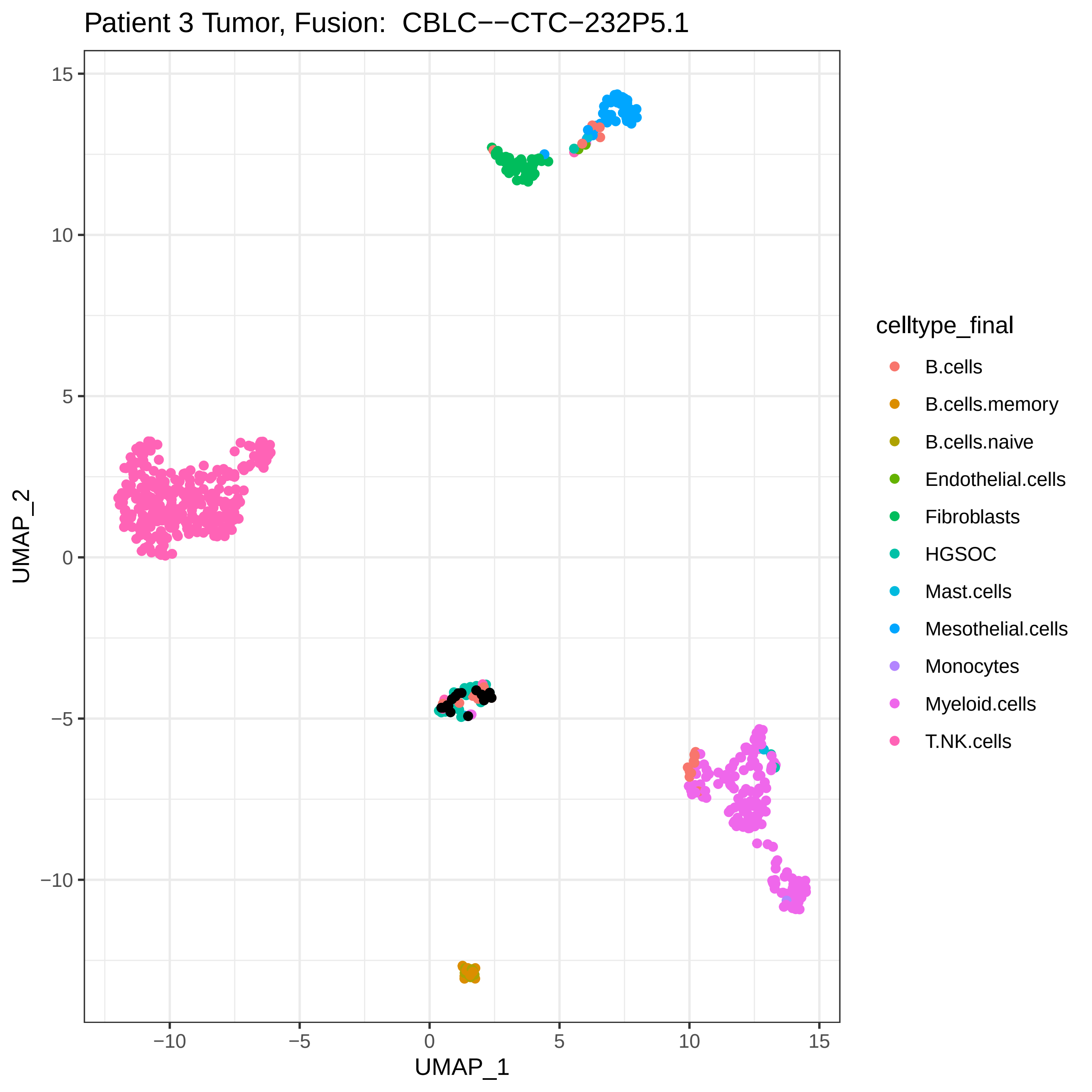
Patient 3 Tumor, Fusion: CBLC−−CTC−232P5.1
UMAP_2
UMAP_1
15
10
5
0
−5
−10
−10
−5
0
5
10
15
celltype_final
B.cells
B.cells.memory
B.cells.naive
Endothelial.cells
Fibroblasts
HGSOC
Mast.cells
Mesothelial.cells
Monocytes
Myeloid.cells
T.NK.cells

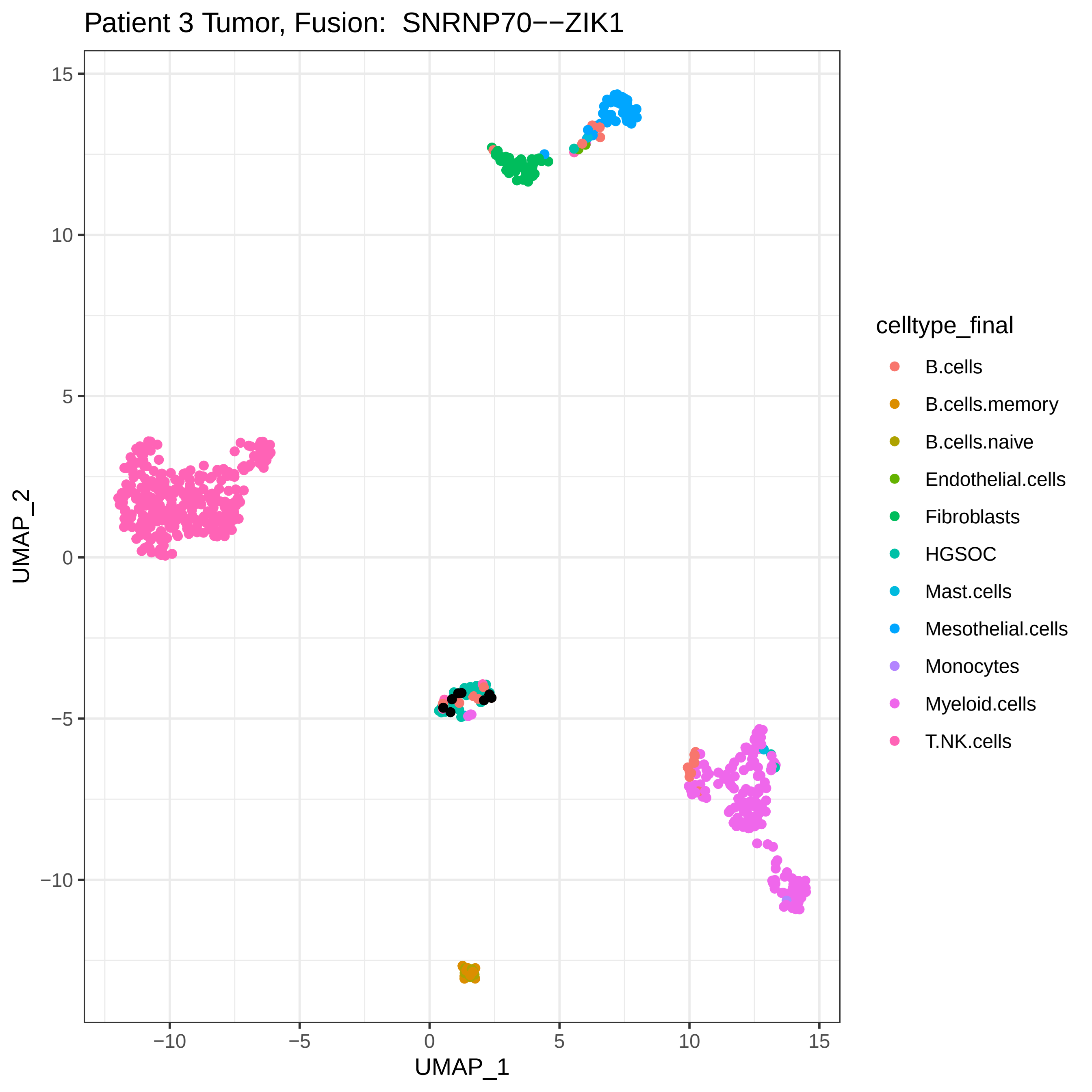

Patient 3 Tumor, Fusion: SNRNP70−−ZIK1
UMAP_2
UMAP_1
15
10
5
0
−5
−10
−10
−5
0
5
10
15
celltype_final
B.cells
B.cells.memory
B.cells.naive
Endothelial.cells
Fibroblasts
HGSOC
Mast.cells
Mesothelial.cells
Monocytes
Myeloid.cells
T.NK.cells
