# Supplementary material for: Accurate fusion transcript identification from long- and short-read isoform sequencing at bulk or single-cell resolution
Source: Genome Res. 2025 Apr;35(4):967–86. doi: 10.1101/gr.279200.124 (PMC12047241; doi:10.1101/gr.279200.124)
Supplement: Supplement 11 [file Supplemental_File_1.zip › CTAT-LR-fusion.v1.0.0/FusionFilter/FusionFilter.github.io/index.html]

FusionFilter Redirect to Wiki


FusionFilter documentation is found on the wiki here. Click the link \*only\* if not auto-redirected.
